# Data-driven control of complex networks

**DOI:** 10.1038/s41467-021-21554-0

**Published:** 2021-03-03

**Authors:** Giacomo Baggio, Danielle S. Bassett, Fabio Pasqualetti

**Affiliations:** 1 Department of Information Engineering, University of Padova, Padova, Italy; 2 Department of Bioengineering, University of Pennsylvania, Philadelphia, PA USA; 3 Department of Physics and Astronomy, University of Pennsylvania, Philadelphia, PA USA; 4 Department of Electrical and Systems Engineering, University of Pennsylvania, Philadelphia, PA USA; 5 Department of Neurology, University of Pennsylvania, Philadelphia, PA USA; 6 Department of Psychiatry, University of Pennsylvania, Philadelphia, PA USA; 7 Santa Fe Institute, Santa Fe, NM USA; 8 Department of Mechanical Engineering, University of California at Riverside, Riverside, CA USA

**Keywords:** Electrical and electronic engineering, Applied mathematics

## Abstract

Our ability to manipulate the behavior of complex networks depends on the design of efficient control algorithms and, critically, on the availability of an accurate and tractable model of the network dynamics. While the design of control algorithms for network systems has seen notable advances in the past few years, knowledge of the network dynamics is a ubiquitous assumption that is difficult to satisfy in practice. In this paper we overcome this limitation, and develop a data-driven framework to control a complex network optimally and without any knowledge of the network dynamics. Our optimal controls are constructed using a finite set of data, where the unknown network is stimulated with arbitrary and possibly random inputs. Although our controls are provably correct for networks with linear dynamics, we also characterize their performance against noisy data and in the presence of nonlinear dynamics, as they arise in power grid and brain networks.

## Abstract (web summary)

Controlling the behavior of a complex network usually requires a knowledge of the network dynamics. Baggio et al. propose a data-driven framework to control a complex dynamical network, effective for non-complete or random datasets, which is of relevance for power grids and neural networks.

## Introduction

With the development of sensing, processing, and storing capabilities of modern sensors, massive volumes of information-rich data are now rapidly expanding in many physical and engineering domains, ranging from robotics^1^ to biological^2,3^ and economic sciences^4^. Data are often dynamically generated by complex interconnected processes, and encode key information about the structure and operation of these networked phenomena. Examples include temporal recordings of functional activity in the human brain^5^, phasor measurements of currents and voltages in the power distribution grid^6^, and streams of traffic data in urban transportation networks^7^. When first-principle models are not conceivable, costly, or difficult to obtain, this unprecedented availability of the data offers a great opportunity for scientists and practitioners to better understand, predict, and, ultimately, control the behavior of real-world complex networks.

**Fig. 1:**
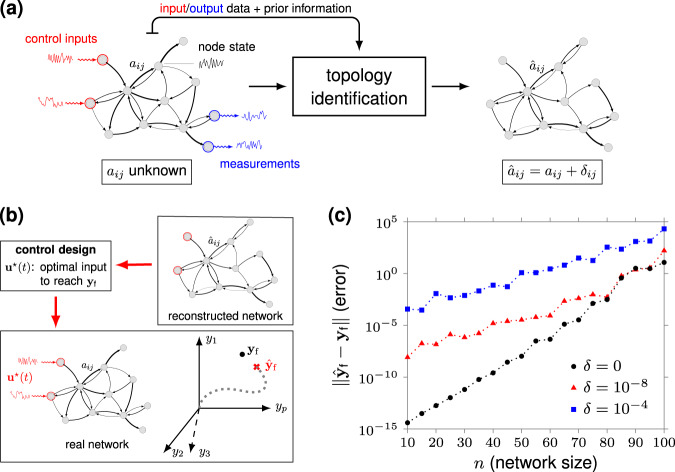
The effect of model uncertainty in the computation of optimal network controls. Panel (**a**) shows a schematic of a classic network identification procedure. The reconstructed network is affected by estimation errors *δ*_*ij*_. The symbol {*a*_*ij*_} denote the correct network weights, whereas $\{\hat{{a}}_{ij}\}$ the (incorrectly) reconstructed ones. Panel (**b**) illustrates the error in the final (output) state induced by an optimal control design based on the reconstructed network. The symbol **y**_f_ denote the desired final state of (a subset of) the network nodes, whereas ${\hat{{\bf{y}}}}_{{\rm{f}}}$ the one reached by the optimal input **u**^⋆^(*t*). In panel (**c**), we consider minimum-energy controls designed from exact and incorrectly reconstructed linear dynamical networks, and compute the resulting error in the final state as the network size *n* varies. We consider connected Erdös–Rényi networks with edge probability ${p}_{{\rm{edge}}}=\mathrm{ln}\,n/n+0.1$, ten randomly selected control nodes, control horizon *T* = 2*n*, and a randomly chosen final state ${{\bf{x}}}_{{\rm{f}}}\in {{\mathbb{R}}}^{n}$. We assume **y**_f_ = **x**_f_, i.e., the nodes that we want to control coincide with all network nodes. To mimic errors in the network reconstruction process, we add to each edge of the network a disturbance modeled as an i.i.d. random variable uniformly distributed in [ − *δ*, *δ*], *δ* > 0. Each curve represents the average of the (norm of the) error in the final state over 100 independent realizations of **x**_f_. To compute minimum-energy control inputs, we use the classic Gramian-based formula and standard LAPACK linear-algebra routines (see “Methods”). Notice that there is a nonzero error in the final state which grows with the size of the network even in the absence of uncertainty (*δ* = 0). This is due to numerical errors in the computation of the minimum-energy control which are a consequence of the ill-conditioning of the Gramian^9,20^.

Existing works on the controllability of complex networks have focused exclusively on a model-based setting^8–14^, although, in practice, constructing accurate models of large-scale networks is a challenging, often unfeasible, task^15–17^. In fact, errors in the network model (i.e., missing or extra links, incorrect link weights) are unavoidable, especially if the network is identified from data^18,19^ (see Fig. 1a). This uncertainty is particularly important for network controllability, since, as exemplified in Fig. 1b, c, the computation of model-based network controls tends to be unreliable and highly sensitive to model uncertainties, even for moderate size networks, if the network is controlled by few nodes^20,21^. It is therefore natural to ask whether network controls can be learned directly from data, and, if so, how well these data-driven control policies perform.

Data-driven control of dynamical systems has attracted increasing interest over the last few years, triggered by recent advances and successes in machine learning and artificial intelligence^22,23^. The classic (indirect) approach to learn controls from data is to use a sequential system identification and control design procedure. That is, one first identifies a model of the system from the available data and then computes the desired controls using the estimated model^24^. However, identification algorithms are sometimes inaccurate and time-consuming, and several direct data-driven methods have been proposed to bypass the identification step^25^. These include, among others, (model-free) reinforcement learning^26,27^, iterative learning control^28^, adaptive and self-tuning control^29^, and behavior-based methods^30,31^.

The above techniques differ in the data-generation procedure, class of system dynamics considered, and control objectives. In classic reinforcement learning settings, data are generated online and updated under the guidance of a policy evaluator or reward simulator, which in many applications is represented by an offline-trained (deep) neural network^32^. Iterative learning control is used to refine and optimize repetitive control tasks: data are recorded online during the execution of a task repeated multiple times, and employed to improve tracking accuracy from trial to trial. In adaptive control, the structure of the controller is fixed and a few control parameters are optimized using data collected on the fly. A widely known example is the auto-tuning of PID controllers^33^. Behavior-based techniques exploit a trajectory-based (or behavioral) representation of the system, and data that typically consist of a single, noiseless, and sufficiently long input–output system trajectory^31^. Each of the above data-driven approaches has its own limitations and merits, which strongly depend on the intended application area. However, a common feature of all these approaches is that they are tailored to or have been employed for closed-loop control tasks, such as stabilization or tracking, and not for finite-time point-to-point control tasks.

In this paper, we address the problem of learning from data point-to-point optimal controls for complex dynamical networks. Precisely, following recent literature on the controllability of complex networks^34,35^, we focus on control policies that optimally steer the state of (a subset of) network nodes from a given initial value to a desired final one within a finite-time horizon. To derive analytic, interpretable results that capture the role of the network structure, we consider networks governed by linear dynamics, quadratic cost functions, and data consisting of a set of control experiments recorded offline. Importantly, experimental data are not required to be optimal, and can even be generated through random control experiments. In this setting, we establish closed-form expressions of optimal data-driven control policies to reach the desired target state and, in the case of noiseless data, characterize the minimum number of experiments needed to exactly reconstruct optimal control inputs. Further, we introduce suboptimal yet computationally simple data-driven expressions and discuss certain numerical and computational advantages of using our data-driven approach when compared to classic model-based ones. Finally, we illustrate with different numerical studies how our framework can be applied to (i) induce prescribed patterns of synchronization in networks of oscillators, (ii) restore the correct operation of power-grid networks after a fault, and (iii) characterize the controllability properties of functional brain networks.

While the focus of this paper is on designing optimal control inputs, the expressions derived in this work could also serve as an alternative, computationally reliable, and efficient way of (i) analyzing the controllability properties of network systems and (ii) solving control-related network problems, such as the optimal selection of control and sensor nodes. In particular, as a by-product of our analysis, we show that (output) controllability can be assessed directly and simply from data. This constitutes an additional methodological contribution to the extensive literature on the model-based analysis of network controllability.

## Results

### Network dynamics and optimal point-to-point control

We consider networks governed by linear time-invariant dynamics

1 $${\bf{x}}(t+1)=	 \;{\bf{A}}{\bf{x}}(t)+{\bf{B}}{\bf{u}}(t),\\ {\bf{y}}(t)=	 \;{\bf{C}}{\bf{x}}(t),$$

where ${\bf{x}}(t)\in {{\mathbb{R}}}^{n}$, ${\bf{u}}(t)\in {{\mathbb{R}}}^{m}$, and ${\bf{y}}(t)\in {{\mathbb{R}}}^{p}$ denote, respectively, the state, input, and output of the network at time *t*. The matrix ${\bf{A}}\in {{\mathbb{R}}}^{n\times n}$ describes the (directed and weighted) adjacency matrix of the network, and the matrices ${\bf{B}}\in {{\mathbb{R}}}^{n\times m}$ and ${\bf{C}}\in {{\mathbb{R}}}^{p\times n}$, respectively, are typically chosen to single out prescribed sets of input and output nodes of the network.

In this work, we are interested in solving point-to-point control problems; that is, designing open-loop control policies that steer the network output **y**(*t*) from an initial value **y**(0) = **y**_0_ to a desired one **y**(*T*) = **y**_f_ in a finite number of steps *T*. If **y**_f_ is output controllable in *T* steps (a standing assumption in this paper; we refer to Supplementary Note 1 for more details), then the latter problem admits a solution and, in fact, there are many ways to accomplish such a control task. Here, we assume that the network is initially relaxed (**x**(0) = **0**), and we seek the control input ${{\bf{u}}}_{0:T-1}^{\star }={[{{\bf{u}}}^{\star }{(T-1)}^{{\mathsf{T}}}\cdots {{\bf{u}}}^{\star }{(0)}^{{\mathsf{T}}}]}^{{\mathsf{T}}}$ that drives the output of the network to **y**_f_ in *T* steps and, at the same time, minimizes a prescribed quadratic combination of the control effort and locality of the controlled trajectories.

Mathematically, we study and solve the following constrained minimization problem:

2 $${{\bf{u}}}_{0:T-1}^{\star }=\arg \mathop{\min }\limits_{{{\bf{u}}}_{0:T-1}} 	{{\bf{y}}}_{1:T-1}^{{\mathsf{T}}}\ {\bf{Q}}\ {{\bf{y}}}_{1:T-1}+{{\bf{u}}}_{0:T-1}^{{\mathsf{T}}}\ {\bf{R}}\ {{\bf{u}}}_{0:T-1}\\ \,	{\text{s.t.}}\,\,\,{\text{Eq.}}\,(1)\,\,{\text{and}}\,\,{{\bf{y}}}_{T}={{\bf{y}}}_{{\mathrm{f}}},$$

where **Q** ≽ **0** and **R** ≻ **0** are tunable (positive semidefinite and positive definite, respectively) matrices that penalize output deviation and input usage, respectively, ${{\bf{y}}}_{1:T-1}={[{\bf{y}}{(1)}^{{\mathsf{T}}}{\bf{y}}{(2)}^{{\mathsf{T}}}\cdots {\bf{y}}{(T-1)}^{{\mathsf{T}}}]}^{{\mathsf{T}}}$, and **y**_*T*_ = **y**(*T*). Problem (2) generalizes the classic (open-loop) linear–quadratic control framework by including the possibility of minimizing a linear function of the state (as opposed to the whole state) in addition to the control input. Further, we remark that increasing **R** in Eq. (2) leads to optimal control inputs that achieve the desired final state with increasingly smaller magnitudes. Similarly, the matrix **Q** in Eq. (2) weighs the norm of the output (state), so that increasing **Q** forces the optimization problem to generate inputs that limit the norm of the output (state), at the expenses of using a larger control input^36^. In particular, if **Q** = **0** and **R** = **I**, then ${{\bf{u}}}_{0:T-1}^{\star }$ coincides with the minimum-energy control to reach **y**_f_ in *T* steps^9,37^.

Equation (2) admits a closed-form solution whose computation requires the exact knowledge of the network matrix **A** and suffers from numerical instabilities (“Methods”). In the following section, we address this limitation by deriving model-free and reliable expressions of ${{\bf{u}}}_{0:T-1}^{\star }$ that solely rely on experimental data collected during the network operation.

### Learning optimal controls from non-optimal data

**Fig. 2:**
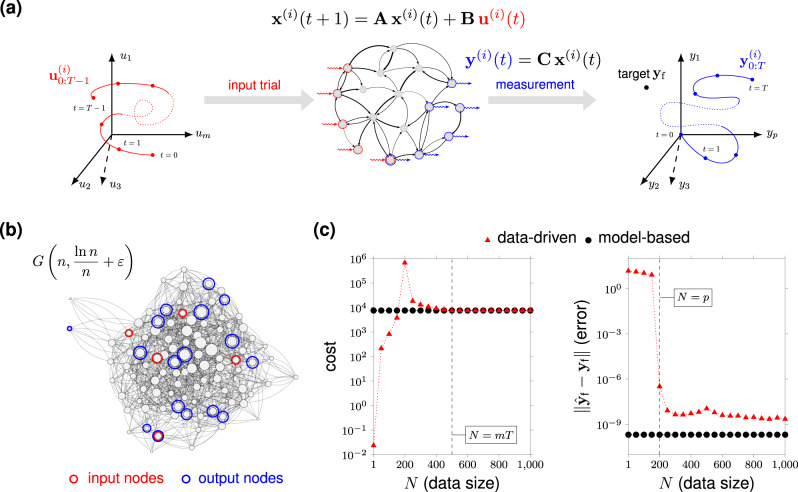
Experimental setup and optimal data-driven network controls. Panel (**a**) illustrates the data-collection process. With reference to the *i*th control experiment, a *T*-step input sequence ${{\bf{u}}}_{0:T}^{(i)}$ excites the network dynamics in Eq. (1), and the time samples of the resulting output trajectory ${{\bf{y}}}_{0:T}^{(i)}$ are recorded. The input trajectory ${{\bf{u}}}_{0:T}^{(i)}$ may be generated randomly, so that the final output ${{\bf{y}}}_{T}^{(i)}$ does not normally coincide with the desired target output **y**_f_. Red nodes denote the control or input nodes (forming matrix **B**) and the blue nodes denote the measured or output nodes (forming matrix **C**). Panel (**b**) shows a realization of the Erdös–Rényi graph model *G*(*n*, *p*_edge_) used in our examples, where *n* is the number of nodes, *p*_edge_ is the edge probability. We set the edge probability to ${p}_{\text{edge}}=\mathrm{ln}\,n/n+\varepsilon$, *ε* = 0.05, to ensure connectedness with high probability, and normalize the resulting adjacency matrix by $\sqrt{n}$. Panel (**c**) shows the value of the cost function (left) and the (norm of the) error in the final state (right) for the data-driven input (4) and the model-based control as a function of the number of data points. The symbol **y**_f_ denotes the desired final target and ${\hat{{\bf{y}}}}_{\text{f}}$ the output reached by the (model-based or data-driven) control input. We choose **Q** = **R** = **I**, *n* = 1000, *T* = 10, *m* = 50, and *p* = 200, and consider Erdös–Rényi networks as in panel (**b**). For additional details, see “Methods”.

We assume that the network matrix **A** is unknown and that *N* control experiments have been performed with the dynamical network in Eq. (1). The *i*th experiment consists of generating and applying the input sequence ${{\bf{u}}}_{0:T-1}^{(i)}$, and measuring the resulting output trajectory ${{\bf{y}}}_{0:T}^{(i)}$ (Fig. 2a). Here, as in ref. ^38^, we consider episodic experiments where the network state is reset to zero before running a new trial, and refer to Supplementary Note 5 for an extension to the nonepisodic setting and to the case of episodic experiments with nonzero initial state resets. We let **U**_0:*T*−1_, **Y**_1:*T*−1_, and **Y**_*T*_ denote the matrices containing, respectively, the experimental inputs, the output measurements in the time interval [1, *T* − 1], and the output measurements at time *T*. Namely,

3 $${{\bf{U}}}_{0:T-1}= 	\,\left[{{\bf{u}}}_{0:T-1}^{(1)}\,\,\cdots \,\,{{\bf{u}}}_{0:T-1}^{(N)}\right],\\ {{\bf{Y}}}_{1:T-1}= 	\,\left[{{\bf{y}}}_{1:T-1}^{(1)}\,\,\cdots \,\,{{\bf{y}}}_{1:T-1}^{(N)}\right],\\ {{\bf{Y}}}_{T}= 	\,\left[{{\bf{y}}}_{T}^{(1)}\,\,\cdots \,\,{{\bf{y}}}_{T}^{(N)}\right].$$

An important aspect of our analysis is that we do not require the input experiments to be optimal, in the sense of Eq. (2), nor do we investigate the problem of experiment design, i.e., generating data that are informative for our problem. In our setting, data are given, and these are generated from arbitrary, possibly random, or carefully chosen experiments.

By relying on the data matrices in Eq. (3), we derive the following data-driven candidate solution to the minimization problem in Eq. (2) (Supplementary Note 2):

4 $${\hat{{\bf{u}}}}_{0:T-1}={{\bf{U}}}_{0:T-1}({\bf{I}}-{{\bf{K}}}_{{{\bf{Y}}}_{T}}{({\bf{L}}{{\bf{K}}}_{{{\bf{Y}}}_{T}})}^{\dagger }{\bf{L}}){{\bf{Y}}}_{T}^{\dagger }\ {{\bf{y}}}_{{\rm{f}}},$$

where **L** is any matrix satisfying ${{\bf{L}}}^{{\mathsf{T}}}{\bf{L}}={{\bf{Y}}}_{1:T-1}^{{\mathsf{T}}}{\bf{Q}}{{\bf{Y}}}_{1:T-1}+{{\bf{U}}}_{0:T-1}^{{\mathsf{T}}}{\bf{R}}{{\bf{U}}}_{0:T-1}$, ${{\bf{K}}}_{{{\bf{Y}}}_{T}}$ denotes a matrix whose columns form a basis of the kernel of **Y**_*T*_, and the superscript symbol ⋅ ^†^ stands for the Moore–Penrose pseudoinverse operation^39^. We remark that the dependence of Eq. (4) on the (unknown) network parameters **A**, **B**, and **C** is implicit and encoded in the collected data **U**_0:*T*−1_, **Y**_1:*T*−1_, and **Y**_*T*_. Further, we stress that ${\hat{{\bf{u}}}}_{0:T-1}$ in Eq. (4) does not, in general, coincide with the optimal solution ${{\bf{u}}}_{0:T-1}^{\star }$ in Eq. (2). However, if enough linearly independent data are collected, then ${\hat{{\bf{u}}}}_{0:T-1}={{\bf{u}}}_{0:T-1}^{\star }$, as we illustrate next.

### Minimum number of the data to learn optimal controls and a data-based controllability condition

Finite data suffice to exactly reconstruct the optimal control input via the data-driven expression in Eq. (4). The minimum number of data ${N}_{\min }$ required to accomplish such a task depends both on the target **y**_f_ and the available data matrices, which are in turn implicitly dependent on the (unknown) parameters **A**, **B**, and **C**. However, it is possible to establish a simple upper bound on ${N}_{\min }$. Namely, if the input data matrix in Eq. (3) contains *mT* linearly independent experiments, that is, if **U**_0:*T*−1_ has full row rank, then ${N}_{\min }\le mT$ (Supplementary Note 3). We stress that linear independence of the control experiments is a mild condition that is normally satisfied when the experiments are generated randomly. Further, if the number of independent trials is smaller than *mT* but such that **y**_f_ belongs to the range space of **Y**_*T*_, then the data-driven control ${\hat{{\bf{u}}}}_{0:T-1}$ still correctly steers the network output to **y**_f_ in *T* steps, but with a cost that is typically larger than the optimal one (Supplementary Note 3). In this case, ${\hat{{\bf{u}}}}_{0:T-1}$ is a suboptimal solution to Eq. (2), which becomes optimal if the collected data are made of control experiments that are optimal as well. We stress that for a number *N* ≥ *p* of randomly chosen control experiments, any (output controllable) **y**_f_ normally belongs to the range space of **Y**_*T*_. In Fig. 2c, we illustrate the above observations for the class of Erdös–Rényi networks of Fig. 2b.

Finally, as a by-product of the above analysis, it follows that the (output) controllability of a network system can be checked directly and simply from the data. Specifically, if *N* ≥ *mT* linearly independent input experiments are collected with *T* ≥ *n*, then the system is output controllable if and only if the columns of the output data matrix **Y**_*T*_ span the entire space ${{\mathbb{R}}}^{p}$; that is, if and only if **Y**_*T*_ has full row rank (Supplementary Note 4). For *p* = *n*, the latter condition can be used to assess the classic controllability of a network system from data.

### Data-driven minimum-energy control

By letting **Q** = **0** and **R** = **I** in Eq. (4), we recover a data-driven expression for the *T*-step minimum-energy control to reach **y**_f_. We remark that the family of minimum-energy controls has been extensively employed to characterize the fundamental capabilities and limitations of controlling networks, e.g., see^9,11,14^. After some algebraic manipulations, the data-driven minimum-energy control input can be compactly rewritten as (Supplementary Note 5)

5 $${\hat{{\bf{u}}}}_{0:T-1}={({{\bf{Y}}}_{T}{{\bf{U}}}_{0:T-1}^{\dagger })}^{\dagger }\ {{\bf{y}}}_{{\rm{f}}}.$$

The latter expression relies on the final output measurements only (matrix **Y**_*T*_) and, thus, it does not exploit the full output data (matrix **Y**_1:*T*−1_). An alternative control expression is

6 $${\tilde{{\bf{u}}}}_{0:T-1}={{\bf{U}}}_{0:T-1}{{\bf{Y}}}_{T}^{\dagger }\ {{\bf{y}}}_{{\rm{f}}}.$$

This is a simple, suboptimal data-based control sequence that correctly steers the network to **y**_f_ in *T* steps, as long as **y**_f_ belongs to the range space of **Y**_*T*_ (a condition that is normally satisfied when *p* randomly generated data are available). Further, and more importantly, when the input data samples are drawn randomly and independently from a Gaussian distribution with zero mean and finite variance, Eq. (6) converges to the minimum-energy control in the limit of infinite data (Supplementary Note 6).

**Fig. 3:**
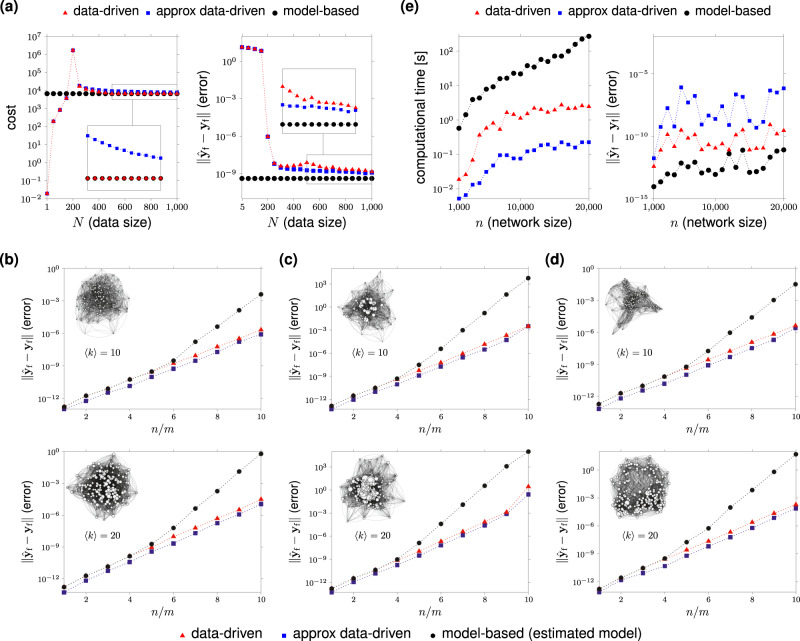
Performance of minimum-energy data-driven network controls. Panel (**a**) shows the value of the cost function (left) and the (norm of the) error in the final state (right) for the minimum-energy data-driven controls (5) and (6), and the model-based one as a function of the data size *N*. We consider Erdös–Rényi networks as in Fig. 2b with *ε* = 0.05, and parameters *n* = 1000, *T* = 10, *m* = 50, *p* = 200. In panels (**b**–**d**), we assume **C** = **I** and compare the error in the final state generated by the data-driven minimum-energy controls (5) and (6) and model-based expression for a fixed number of control nodes *m* = 100 and increasing dimension *n* ∈ [100, 1000]. The model-based control has been computed by first estimating matrices **A** and **B** from data according to the subspace-based technique in “Methods”, and then using the model-based control expression. In panel (**b**), we consider Erdös–Rényi networks with average degree 〈*k*〉 = 10 (top) and 〈*k*〉 = 20 (bottom). In panel (**c**), we consider Barabasi–Albert networks^71^ with the initial number of nodes *m*_0_ = 20 and average degree 〈*k*〉 = 10 (top) and 〈*k*〉 = 20 (bottom). In panel (**d**), we consider Watts–Strogatz networks^72^ with rewiring probability *p*_rew_ = 0.2 and average degree 〈*k*〉 = 10 (top) and 〈*k*〉 = 20 (bottom). In all plots, we use a control horizon *T* = 15 and a number of the data *N* = *mT* + 200. To limit the influence of eigenvalues in the computation of optimal controls across different network models, we normalize matrix **A** by its norm ∥**A**∥. The curves represent the average of over 100 realizations of networks, data, control nodes, and final states. Panel (**e**), left, compares the time needed to compute the optimal controls via data-driven and model-based strategies as a function of the network size, for one realization of the Erdös–Rényi model of Fig. 2b and data. Panel (**e**), right, shows the errors in the final state. We use the following parameters: *ε* = 0.05, *m* = ⌊*n*/100⌋, *p* = ⌊*n*/50⌋, *T* = 50, and *N* = *mT* + 100. For additional details, see “Methods”.

Figure 3a compares the performance (in terms of control effort and error in the final state) of the two data-driven expressions in Eqs. (5) and (6), and the model-based control as a function of the data size *N*. While the data-driven control in Eq. (5) becomes optimal for a finite number of data (precisely, for *N* = *mT* independent data), the approximate expression in Eq. (6) tends to the optimal control only asymptotically in the number of data (Fig. 3a, left). In both cases, the error in the final state goes to zero after collecting *N* = *p* data (Fig. 3a, right). For the approximate control in Eq. (6), we also establish upper bounds on the size of the dataset to get a prescribed deviation from the optimal control, in the case of Gaussian input data. Our nonasymptotic analysis indicates that this deviation is proportional to the worst-case control energy required to reach a unit-norm target. This, in turn, implies that networks that are easy to control require fewer trials to attain a prescribed approximation error (Supplementary Note 6).

### Numerical and computational benefits of data-driven controls

By relying on the same set of experimental data, in Fig. 3b–d, we compare the numerical accuracy, as measured by the error in the final state, of the data-driven controls in Eqs. (5) and (6) and the minimum-energy control computed via a standard two-step approach comprising a network identification step followed by model-based control design. First, we point out that if some nodes of the network are not accessible (**C** ≠ **I**) and no prior information about the network structure is available, then it is impossible to exactly reconstruct the network matrix **A** using (any number of) data^40^. In contrast, the computation of minimum-energy inputs is always feasible via our data-driven expression, provided that enough independent data are collected. We thus focus on the case in which the state of all nodes can be measured (i.e., **C** = **I**). We first consider Erdös–Rényi networks with a fixed number of control nodes *m* = 100 and increasing dimension *n* ∈ [100, 1000]. To reconstruct the network matrices **A** and **B**, we employ the subspace-based identification technique described in “Methods”. Although both the data-driven and the model-based controls yield a poor numerical accuracy for increasing values of *n*/*m* (which is due to the fact that the energy to control a network typically grows exponentially with the ratio *n*/*m*^9,11,20,21^) the model-based input exhibits a faster growth of the error when compared to the data-driven ones for sufficiently large values of *n*/*m* (Fig. 3b). We find similar tradeoffs for other random network models, namely scale-free (Barabási–Albert model) and small-world (Watts–Strogatz model), as illustrated in Figs. 3c and d, respectively. This poor performance of the standard approach is somehow expected because, independently of the network identification procedure, the standard two-step approach requires a number of operations larger than those required by the data-driven approach, resulting in a potentially higher sensitivity to round-off errors. Also, we empirically observe that the gain in numerical accuracy offered by data-driven control inputs is more significant for dense networks (see also Supplementary Fig. 1). Finally, it is worth noting that the approximate data-driven control given in Eq. (6), even though suboptimal, yields the best accuracy. This is particularly interesting since, for a finite number of the data, Eq. (6) does not have a model-based counterpart.

A further advantage in using data-driven controls over model-based ones arises when dealing with massive networks featuring a small fraction of input and output nodes. Specifically, in Fig. 3e we plot the time needed to numerically compute the data-driven and model-based controls as a function of the size of the network. We focus on Erdös–Rényi networks as in Fig. 2b of dimension *n* ≥ 1000 with ⌊*n*/100⌋ input and output nodes and a control horizon *T* = 50. The model-based control input requires the computation of the first *T* − 1 powers of **A** (“Methods”). The computation of the data-driven expressions in Eqs. (5) and (6) involves, instead, linear-algebraic operations on two matrices (**U**_0:*T*−1_ and **Y**_*T*_) that are typically smaller than **A** when *n* is very large (precisely, when *T* < *n*/*m* and *N* < *n*). Thus, the computation of the control input via the data-driven approach is normally faster than the classic model-based computation (Fig. 3e, left). In particular, the data-driven control given in Eq. (6), although suboptimal, yields the most favorable performance due to its particularly simple expression. Finally, we note that the error in the final state committed by the data-driven controls is always upper bounded by 10^−5^ and thus it has a negligible effect on the control accuracy (Fig. 3e, right).

### Data-driven controls with noisy data

The analysis so far has focused on noiseless data. A natural question is how the data-driven controls behave in the case of noisy data. If the noise is unknown but small in magnitude, then the established data-driven expressions will deviate slightly from the correct values (Supplementary Note 7). However, if some prior information on the noise is known, this information can be exploited to return more accurate control expressions. A particularly relevant case is when data are corrupted by additive i.i.d. noise with zero mean and known variance. Namely, the available data read as

7 $${{\bf{U}}}_{0:T-1}=	 \,{\bar{{\bf{U}}}}_{0:T-1}+{{\mathbf{\Delta }}}_{{\bf{U}}},\\ {{\bf{Y}}}_{1:T-1}= 	\,{\bar{{\bf{Y}}}}_{1:T-1}+{{\mathbf{\Delta }}}_{{\bf{Y}}},\\ {{\bf{Y}}}_{T}=	 \,{\bar{{\bf{Y}}}}_{T}+{{\mathbf{\Delta }}}_{{{\bf{Y}}}_{T}},$$

where ${\bar{{\bf{U}}}}_{0:T-1}$, ${\bar{{\bf{Y}}}}_{1:T-1}$, and ${\bar{{\bf{Y}}}}_{T}$ denote the ground truth values, and **Δ**_**U**_, **Δ**_**Y**_, and ${{\mathbf{\Delta }}}_{{{\bf{Y}}}_{T}}$ are random matrices with i.i.d. entries with zero mean and variance ${\sigma }_{{\bf{U}}}^{2}$, ${\sigma }_{{\bf{Y}}}^{2}$, and ${\sigma }_{{{\bf{Y}}}_{T}}^{2}$, respectively. In this setting, it can be shown that the data-driven control in Eq. (4) and the data-driven minimum-energy controls in Eqs. (5) and (6) are typically not consistent; that is, they do not converge to the true control inputs as the data size tends to infinity (see Supplementary Note 7 for a concrete example). However, by suitably modifying these expressions, it is possible to recover asymptotically correct data-driven formulas (Supplementary Note 7). The key idea is to add correction terms that compensate for the noise variance arising from the pseudoinverse operations. In particular, the asymptotically correct version of the data-driven controls in Eqs. (5) and (6) read, respectively, as

8 $${\hat{{\bf{u}}}}_{0:T-1}^{\,\text{(c)}\,}={({{\bf{Y}}}_{T}{{\bf{U}}}_{0:T-1}^{{\mathsf{T}}}{({{\bf{U}}}_{0:T-1}{{\bf{U}}}_{0:T-1}^{{\mathsf{T}}}-N{\sigma }_{{\bf{U}}}^{2}{\bf{I}})}^{\dagger })}^{\dagger }{{\bf{y}}}_{{\rm{f}}},$$

9 $${\tilde{{\bf{u}}}}_{0:T-1}^{\,\text{(c)}\,}={{\bf{U}}}_{0:T-1}{{\bf{Y}}}_{T}^{{\mathsf{T}}}{({{\bf{Y}}}_{T}{{\bf{Y}}}_{T}^{{\mathsf{T}}}-N{\sigma }_{{{\bf{Y}}}_{T}}^{2}{\bf{I}})}^{\dagger }\ {{\bf{y}}}_{{\rm{f}}},$$

where we used the fact that ${{\bf{X}}}^{\dagger }={{\bf{X}}}^{{\mathsf{T}}}{({\bf{X}}{{\bf{X}}}^{{\mathsf{T}}})}^{\dagger }$ for any matrix **X**^39^, and $N{\sigma }_{{\bf{U}}}^{2}{\bf{I}}$ and $N{\sigma }_{{{\bf{Y}}}_{T}}^{2}{\bf{I}}$ represent the noise-dependent correction terms. Note, in particular, that if the noise corrupts the output data **Y**_*T*_ only, then Eq. (8) coincides with the original data-driven control in Eq. (5), so that no correction is needed. Similarly, if the noise corrupts the input data **U**_*T*_ only, then Eq. (9) coincides with the data-driven control in Eq. (6).

### Data-driven pattern control of synchronized activity in Kuramoto networks

The problem of inducing desired patterns of synchronized activity in networks of oscillators has several applications in many natural and technological networks^41,42^. For instance, in the clinical treatment of neurological disorders^43–46^ and in the administration and dispatch of power in distribution networks^47–49^. For these reasons, several methods have been investigated in the literature for the control of synchronized patterns of activity^46,49–51^. Here, we show how the data-driven framework proposed in this paper can be employed to provide a solution to this problem.

**Fig. 4:**
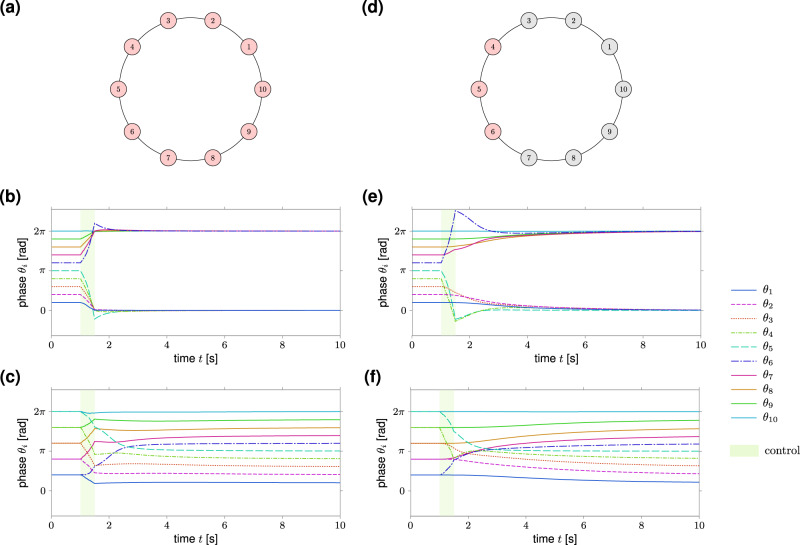
Data-driven control of synchronized patterns in a ring of Kuramoto oscillators. We consider a ring network of *n* = 10 Kuramoto oscillators for two different configurations of control nodes (red nodes), namely *m* = 10 (**a**) and *m* = 3 (**d**). For both configurations, we apply the data-driven control (Eq. (4) of the main text) to steer the phases from the splay state $\{\bar{\theta }_{1,i}(t)\}$ to the synchronous state (**b**, **e**) and from the splay state $\{\bar{\theta }_{2,i}(t)\}$ to $\{\bar{\theta }_{1,i}(t)\}$ (**c**, **f**). The green region denotes the application of the control. We choose as parameters *T* = 50 samples of the discretized dynamics (corresponding to a control horizon of 0.5 s), **Q** = 5**I**, **R** = **I**, and *N* = 1000 data obtained by perturbing the initial equilibrium with i.i.d. Gaussian inputs with zero mean and standard deviation 0.1.

To this end, we consider a simple yet insightful example, that is, a ring network of *n* Kuramoto oscillators. The dynamics of the phases of the oscillators are

10 $${\dot{\theta }}_{i}(t)={\omega }_{i}+\sin ({\theta }_{i-1}(t)-{\theta }_{i}(t))+\sin ({\theta }_{i+1}(t)-{\theta }_{i}(t)),\,\,\,i=1,\ldots ,\,n,$$

where *ω*_*i*_ is the natural frequency of the *i*th oscillator and the index *i* is periodic ${\rm{mod}}\,n$. We consider the case where *ω*_*i*_ = *ω* for all *i*. In this case, the network always has a stable synchronous state given by ${\bar{\theta }}_{i}(t)=\omega t$ for all *i*. However, as *n* grows other stable equilibria arise, namely^52^:

11 $${\bar{\theta }}_{q,i}(t)=\omega t+\frac{2\pi qi}{n}+c,\,\,i=1,\ldots ,\,n,$$

where *c* is an arbitrary constant and *q* is the winding number which takes integer values. These equilibria correspond to phases linearly spaced on the unit circle and are commonly referred to as splay states. To simulate the dynamics in Eq. (10) and generate the data, we discretize the Kuramoto dynamics via the forward Euler method with discretization step 0.01. In Fig. 4, we consider a network of *n* = 10 oscillators with *ω* = 0. In the top plots, we assume that we have access to all nodes of the network (*m* = 10) and apply an external control input to steer the network: (i) from the splay state $\{\bar{{\theta }}_{1,i}(t)\}$ to the synchronous state $\{\bar{\theta }_{i}(t)\}$ (Fig. 4b), and (ii) from the splay state $\{\bar{\theta }_{2,i}(t)\}$ to the splay state $\{\bar{\theta }_{1,i}(t)\}$ (Fig. 4c). The control input has been computed using the data-driven expression given in Eq. (4) with *T* = 50 samples (corresponding to a control horizon of 0.5 s), and parameters **Q** = 5**I**, **R** = **I**. Further, we subtract to each output data sample in matrix **Y**_1:*T*−1_ the value of the final equilibrium point, so that choosing a sufficiently large **Q** favor trajectories with a small deviation from the equilibrium. We remark that the choice of **Q** is particularly important when applying our expressions to nonlinear networks. In this case, choosing a large **Q** often improves the applicability and effectiveness of our methods, since a nonlinear system approximately behaves as a linear one in a sufficiently small neighborhood of equilibrium (see also Supplementary Fig. 2). Data have been generated through *N* = 1000 control experiments obtained by perturbing the initial equilibrium with i.i.d. Gaussian external inputs with zero mean and standard deviation 0.1. In the right plots of Fig. 4, we repeat the same experiments using *m* = 3 control nodes (red nodes in Fig. 4d). In both scenarios, the control input does not exactly drive the network to the desired target state (because of the nonlinearity of the dynamics) but to a state close to it. Nevertheless, the final state falls within the basin of attraction of the desired target equilibrium so that the network reaches asymptotically the desired pattern of synchronization. Finally, we point out that the above-described procedure can, in principle, be applied to more complex (possibly random) network topologies. In such cases, however, it is typically more challenging to determine the initial and final equilibrium configurations that specify the considered point-to-point control problem^53^.

### Data-driven fault recovery in power-grid networks

**Fig. 5:**
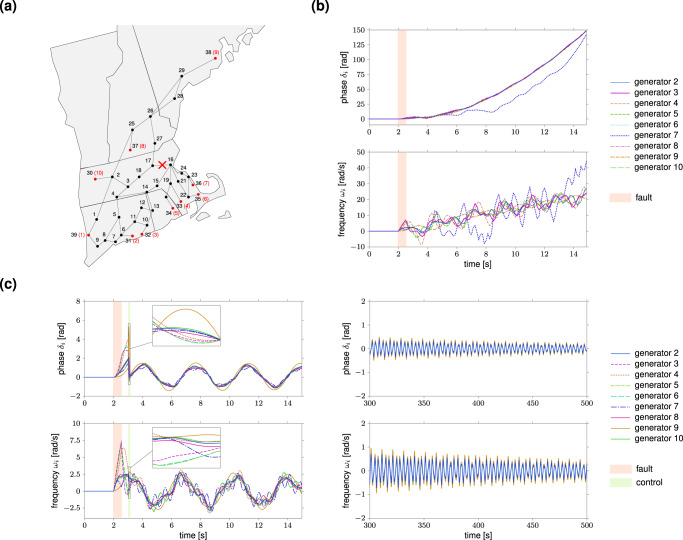
Data-driven fault recovery in the New England power-grid network. Panel (**a**) depicts the 39-node New England power-grid network (see ref. ^70^, Appendix A). The black nodes {1, …, 29} represent load nodes, while the red nodes {30, …, 39} are power generators. The generators are labeled according to the numbers in the red brackets. The red cross denotes the location of the fault. Panel (**b**) plots the behavior of the phases and frequencies of generators {2, …, 10} after the occurrence of the fault. The onset time of the fault is *t* = 2 s and the fault duration is 0.5 s (red area in the plots). At time *t* = 2.5 s the fault is cleared. The phase and frequency of generator 1 (not shown) are fixed to a constant (see “Methods”). The left plots of the panel (**c**) show the behavior of the phases and frequencies of generators {2, …, 10} after the application of the data-driven control input (4). The duration of the control action is 0.1 s (green area in the plots) which corresponds to a control horizon *T* = 400 for the discretized network dynamics with sampling period *T*_*s*_ = 2.5 × 10^−4^ s. For the computation of the control input, we employ *N* = 4000 experimental data collected offline by perturbing the state of the generators locally around its steady-state value (see “Methods”). We use weighting matrices **R** = **I** and **Q** = *ε***I**, with *ε* = 0.01. The insets illustrate the behavior of the phases and frequencies during the application of the control. The right plots of the panel (**c**) show the asymptotic behavior of the phases and frequencies of generators {2, …, 10} after the application of the data-driven control (4).

We address the problem of restoring the normal operation of a power-grid network after the occurrence of a fault that desynchronizes part of the grid. If not mitigated in a timely manner, such desynchronization instabilities may trigger cascading failures that can ultimately cause major blackouts and grid disruptions^54–56^. In our case study, we consider a line fault in the New England power-grid network comprising 39 nodes (29 load nodes and 10 generator nodes), as depicted in Fig. 5a, and we compute an optimal point-to-point control from data to recover the correct operation of the grid. A similar problem is solved in ref. ^55^ using a more sophisticated control strategy which requires knowledge of the network dynamics. As in refs. ^54,55^, we assume that the phase *δ*_*i*_ and the (angular) frequency *ω*_*i*_ of each generator *i* obey the swing equation dynamics with the parameters given in ref. ^54^ (except for generator 1 whose phase and frequency are fixed to a constant, cf. “Methods”). Initially, each generator operates at a locally stable steady-state condition determined by the power-flow equations. At time *t* = 2 s, a three-phase fault occurs in the transmission line connecting nodes 16 and 17. After 0.5 s, the fault is cleared; however, the generators have lost synchrony and deviate from their steady-state values (Fig. 5b). To recover the normal behavior of the grid, 0.5 s after the clearance of the fault, we apply a short, optimal control input to the frequency of the generators to steer the state (phase and frequency) of the generators back to its steady-state value. The input is computed from data via Eq. (4) using *N* = 4000 input/state experiments collected by locally perturbing the state of the generators around its normal operation point using the real, nonlinear swing dynamics (see also “Methods”). We consider data sampled with period *T*_*s*_ = 2.5 × 10^−4^ s, and set the control horizon to *T* = 400 time samples (corresponding to 0.1 s), **R** = **I**, and **Q** = *ε***I** with *ε* = 0.01 to enforce locality of the controlled trajectories. As shown in Fig. 5c, the data-driven input drives the state of the generators to a point close enough to the starting synchronous solution (left, inset) so as to asymptotically recover the correct operation of the grid (right). We remark that, because of the nonlinearity of the dynamics, the data-driven control input is not able to exactly steer the state the network back to the original synchronous state, but to a point close to it. The latter point, however, falls within the basin of attraction of the synchronous solution. Thus, the control input is able to correctly steer the network to the desired synchronous state, although not in finite time. Notably, as previously discussed, the computation of the control input requires only pre-collected data, is numerically efficient, and optimal (for the linearized dynamics). More generally, this numerical study shows that the data-driven strategy in Eq. (4) could represent a simple, viable, and computationally efficient approach to control complex nonlinear networks around an operating point.

### Controlling functional brain networks via fMRI snapshots

**Fig. 6:**
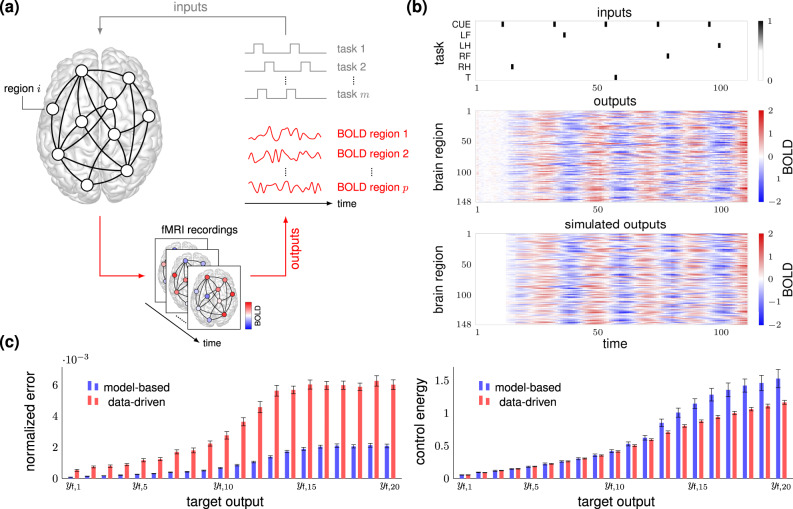
Data-driven control of functional brain networks. Panel (**a**) provides a schematic of the experimental setup. A set of external stimuli represented by *m* different task commands induce brain activity. Functional magnetic resonance (fMRI) blood oxygen level-dependent (BOLD) signals are measured and recorded at different times and converted into *p* time series, one for each brain region. The top and center heatmaps of the panel (**b**) show the inputs and outputs, respectively, for the first 110 measurements of one subject of the HCP dataset. The inputs are divided into *m* = 6 channels corresponding to different task conditions, i.e., CUE (a visual cue preceding the occurrence of other task conditions), LF (squeeze left toe), LH (tap left fingers), RF (squeeze right toe), RH (tap right finger), and T (move tongue). As in ref. ^58^, each input is a binary 0–1 signal taking the value 1 when the corresponding task condition is issued and 0 otherwise. The outputs represent the BOLD signals of the *p* = 148 brain regions obtained from and enumerated according to the Destrieux 2009 atlas^59^. The bottom heatmap of the panel (**b**) displays the simulated outputs obtained by exciting the approximate low-dimensional linear model of ref. ^58^ with the input sequence of the top plot. In panel (**c**), we compare the performance of the data-driven and model-based strategy, assuming that the dynamics obey the above-mentioned approximate linear model. We set the control horizon to *T* = 100 and generate the data matrices by sliding a time window of size *T* across the data samples. The target state **y**_f,*i*_ is the eigenvector associated with the *i*-th eigenvalue of the empirical Gramian matrix ${\hat{{\boldsymbol{{\cal{W}}}}}}_{T}={\hat{{\boldsymbol{{\cal{C}}}}}}_{T}^{{\mathsf{T}}}{\hat{{\boldsymbol{{\cal{C}}}}}}_{T}$, where ${\hat{{\boldsymbol{{\cal{C}}}}}}_{T}={{\bf{Y}}}_{T}{{\bf{U}}}_{0:T-1}^{\dagger }$. The left plot shows the error to reach the targets ${\{{{\bf{y}}}_{\text{f},i}\}}_{i = 1}^{20}$ using the data-driven minimum-energy input in Eq. (5) and the model-based one. The right plot shows the norm of the two inputs. The colored bars denote the mean over 100 unrelated subjects and the error bars are the 95% confidence intervals around the mean.

We investigate the problem of generating prescribed patterns of activity in functional brain networks directly from task-based functional magnetic resonance imaging (task-fMRI) time series. Specifically, we examine a dataset of task-based fMRI experiments related to motor activity extracted from the Human Connectome Project (HCP)^57^ (see Fig. 6a). In these experiments, participants are presented with visual cues that ask them to execute specific motor tasks; namely, tap their left or right fingers, squeeze their left or right toes, and move their tongue. We consider a set of *m* = 6 input channels associated with different task-related stimuli; that is, the motor tasks’ stimuli and the visual cue preceding them. As in ref. ^58^, we encode the input signals as binary time series taking the value of 1 when the corresponding task-related stimulus occurs and 0 otherwise. The output signals consist of minimally pre-processed blood oxygen-level-dependent (BOLD) time series associated with the fMRI measurements at different regions of the brain (see also “Methods”). In our numerical study, we parcellated the brain into *p* = 148 brain regions (74 regions per hemisphere) according to the Destrieux 2009 atlas^59^. Further, as a baseline for comparison, we approximate the dynamics of the functional network with a low-dimensional (*n* = 20) linear model computed via the approach described in ref. ^58^, which has been shown to accurately capture the underlying network dynamics. In fact, although it is widely acknowledged that brain dynamics are nonlinear, linear models can provide a reasonable approximation of the actual nonlinear neural trajectories in certain operating conditions^60,61^.

In Fig. 6b, we plot the inputs (top) and outputs (center) of one subject for the first sequence of five motor tasks. The bottom plot of the same figure shows the outputs obtained by approximating the network dynamics with the above-mentioned linear model. In Fig. 6c, we compare the performance of the minimum-energy data-driven control in Eq. (5) with the model-based one, assuming that the network obeys the dynamics of the approximate linear model. We choose a control horizon *T* = 100, form the data matrices in Eq. (3) by sliding a window of fixed size *T* over the available fMRI data, and consider a set of 20 orthogonal targets ${\{{{\bf{y}}}_{\text{f},i}\}}_{i = 1}^{20}$ corresponding to eigenvectors of the estimated *T*-step controllability Gramian (see “Methods” for further details). The top plot of Fig. 6c reports the error (normalized by the output dimension) in the final state of the two strategies, while the bottom plot shows the corresponding control energy (that is, the norm of the control input). In the plots, the targets are ordered from the most (**y**_f,1_) to the least (**y**_f,20_) controllable. The data-driven and the model-based inputs exhibit an almost identical behavior with reference to the most controllable targets. As we shift towards the least controllable targets, the data-driven strategy yields larger errors but, at the same time, requires less energy to be implemented, thus being potentially more feasible in practice. Importantly, since the underlying brain dynamics are not known, errors in the final state are computed using the identified linear dynamical model. It is thus expected that data-driven inputs yield larger errors in the final state than model-based inputs, although these errors may not correspond to control inaccuracies when applying the data-driven inputs to the actual brain dynamics. Ultimately, our numerical study suggests that the data-driven framework could represent a viable alternative to the classic model-based approach^12,46,62^ to infer controllability properties of brain networks, and (by suitably modulating the reconstructed inputs) enforce desired functional configurations in a non-invasive manner and without requiring real-time measurements.

## Discussion

In this paper, we present a framework to control complex dynamical networks from data generated by non-optimal (and possibly random) experiments. We show that optimal point-to-point controls to reach the desired target state, including the widely used minimum-energy control input, can be determined exactly from data. We provide closed-form and approximate data-based expressions of these control inputs and characterize the minimum number of samples needed to compute them. Further, we show by means of numerical simulations that data-driven inputs present some numerical advantages with respect to classic model-based approaches, and can be used to analyze and manipulate the controllability properties of real networks.

The data-driven expressions derived in this paper are not only theoretically intriguing and practically relevant but they may also provide an alternative set of tools to investigate how different network properties, such as dimension, heterogeneity, and structure, affect controllability. These questions, which are currently being asked in a model-based setting, may find an easier answer in a data-driven framework due to the simplified mathematical expressions of optimal controls. More generally, our framework and results suggest that many network control problems may be solved by simply relying on experimental data, thus promoting a new, exciting, and practical line of research in the field of complex networks. Because of the abundance of data in modern applications and the computationally appealing properties of data-driven controls, we expect that this new line of research will benefit a broad range of research communities, spanning from engineering to biology, which employs control-theoretic methods and tools to comprehend and manipulate complex networked phenomena.

Some limitations of this study should also be acknowledged and discussed. First, in our work we consider networks governed by linear dynamics. On the one hand, this is a restrictive assumption since many real-world networks are inherently nonlinear. On the other hand, linear models are used successfully to approximate the behavior of nonlinear dynamical networks around desired operating points and capture more explicitly the impact of the network topology. Second, in our numerical studies, we employed routines that are commonly used in engineering and scientific computation. Using higher precision routines can alleviate and possibly alter our numerical results. However, since we use the same routines to compare model-based and data-driven methods, we believe that the comparisons in the paper remain qualitatively valid (although possibly with different values) even when using routines with higher precision. Third, in many cases, a closed-loop control strategy is preferable to a point-to-point one, especially if the control objective is to stabilize an equilibrium when external disturbances corrupt the dynamics. However, we stress that point-to-point controls, in addition to being able to steer the network to arbitrary configurations, are extensively used to characterize the fundamental control properties and limitations in networks of dynamical nodes. For instance, the expressions we provide for point-to-point control can also lead to novel, data-based methods to study the energetic limitations of controlling complex networks^9^, select sensors and actuators for optimized estimation and control^63^, and design optimized network structures^64^. Notably, model-based solutions to these control-related problems have been fruitfully applied to shed light on the behavior and operation of real (nonlinear) networks^12,60,61,65^. Finally, although we provide data-driven expressions that compensate for the effect of noise in the limit of infinite data, we do not provide nonasymptotic guarantees on the reconstruction error. Overcoming these limitations represent a compelling direction of future work, which can strengthen the relevance and applicability of our data-driven control framework, and ultimately lead to viable control methods for complex networks.

## Methods

### Model-based expressions of optimal controls

The model-based solution to the problem in Eq. (2) can be written in a batch form as

12 $${{\bf{u}}}_{0:T-1}^{\star }=({\bf{I}}-{{\bf{K}}}_{{{{{\cal{C}}}}}_{T}}{({\bf{M}}{{\bf{K}}}_{{{\boldsymbol{{\cal{C}}}}}_{T}})}^{\dagger }{\bf{M}}){{\boldsymbol{{\cal{C}}}}}_{T}^{\dagger }{{\bf{y}}}_{{\rm{f}}},$$

where ${{\boldsymbol{{\cal{C}}}}}_{T}=[{\bf{C}}{\bf{B}}\,{\bf{C}}{\bf{A}}{\bf{B}}\,\cdots \,{\bf{C}}{{\bf{A}}}^{T-1}{\bf{B}}]$ is the *T*-step output controllability matrix of the dynamical network in Eq. (1), ${{\bf{K}}}_{{{\boldsymbol{{\cal{C}}}}}_{T}}$ denotes a basis of the kernel of ${{\boldsymbol{{\cal{C}}}}}_{T}$, and **M** is any matrix satisfying ${{\bf{M}}}^{{\mathsf{T}}}{\bf{M}}={{\boldsymbol{{\cal{H}}}}}_{T}^{{\mathsf{T}}}{\bf{Q}}{{\boldsymbol{{\cal{H}}}}}_{T}+{\bf{R}}$, with

13 $${{\boldsymbol{{\cal{H}}}}}_{T}=\left[\begin{array}{lllll}{\bf{0}}&\cdots \ &\cdots \ &{\bf{0}}&{\bf{C}}{\bf{B}}\\ \vdots &\cdots \ &{\bf{0}}&{\bf{C}}{\bf{B}}&{\bf{C}}{\bf{A}}{\bf{B}}\\ {\vdots} &{{\kern-2.3pt{.}}\kern2.3pt{\raise4pt\hbox{${.}$}}\kern2.3pt{\raise8pt\hbox{${.}$}}}&{{\kern-2.3pt{.}}\kern2.3pt{\raise4pt\hbox{${.}$}}\kern2.3pt{\raise8pt\hbox{${.}$}}}&{{\kern-2.3pt{.}}\kern2.3pt{\raise4pt\hbox{${.}$}}\kern2.3pt{\raise8pt\hbox{${.}$}}}&{\vdots} \\ {\bf{0}}&{\bf{C}}{\bf{B}}&{\bf{C}}{\bf{A}}{\bf{B}}&\cdots \ &{\bf{C}}{{\bf{A}}}^{T-2}{\bf{B}}\end{array}\right],$$

and 0 entries denoting *p* × *m* zero matrices. If **Q** = **0** and **R** = **I** (minimum-energy control input), Eq. (12) simplifies to ${{\bf{u}}}_{0:T-1}^{\star }={{\boldsymbol{{\cal{C}}}}}_{T}^{\dagger }{{\bf{y}}}_{{\rm{f}}}$. Alternatively, if the network is output controllable, the minimum-energy input can be compactly written as

14 $${{\bf{u}}}^{\star }(t)={{\bf{B}}}^{{\mathsf{T}}}{{\bf{A}}}^{T-t-1}{{\bf{C}}}^{{\mathsf{T}}}{{\boldsymbol{{\cal{W}}}}}_{T}^{-1}{{\bf{y}}}_{{\rm{f}}},\quad t=0,1,2,\ldots ,\,T-1.$$

where ${{\boldsymbol{{\cal{W}}}}}_{T}$ denotes the *T*-step output controllability Gramian of the dynamical network in Eq. (1)

15 $${{\boldsymbol{{\cal{W}}}}}_{T}={{\boldsymbol{{\cal{C}}}}}_{T}{{\boldsymbol{{\cal{C}}}}}_{T}^{{\mathsf{T}}}=\mathop{\sum }\limits_{t=0}^{T-1}{\bf{C}}{{\bf{A}}}^{t}{\bf{B}}{{\bf{B}}}^{{\mathsf{T}}}{({{\bf{A}}}^{{\mathsf{T}}})}^{t}{{\bf{C}}}^{{\mathsf{T}}},$$

which is invertible if and only if the network is output controllable. Equation (14) is the classic (Gramian-based) expression of the minimum-energy control input^37^. It is well-known that this expression is numerically unstable, even for moderate size systems^20^.

### Subspace-based system identification

Given the data matrices **U**_0:*T*−1_ and **Y**_*T*_ as defined in Eq. (3) and assuming that **C** = **I**, a simple deterministic subspace-based procedure^66, Ch. 6]^ to estimate the matrices **A** and **B** from the available data consist of the following two steps:

1. Compute an estimate of the *T*-step controllability matrix of the network as the solution to the minimization problem
  16 $${\hat{{\boldsymbol{{\cal{C}}}}}}_{T}=\arg \mathop{\min }\limits_{{{\boldsymbol{{\cal{C}}}}}_{T}}{\left\Vert {{\bf{Y}}}_{T}-{{\boldsymbol{{\cal{C}}}}}_{T}{{\bf{U}}}_{0:T-1}\right\Vert }_{\,\text{F}\,}^{2},$$
  where ∥ ⋅ ∥_F_ denotes the Frobenius norm of a matrix. The solution to the problem in Eq. (16) has the form ${\hat{{\boldsymbol{{\cal{C}}}}}}_{T}={{\bf{Y}}}_{T}{{\bf{U}}}_{0:T-1}^{\dagger }$.
2. In view of the definition of the controllability matrix, obtain an estimate of the matrix **B** by extracting the first *m* columns of ${\hat{{\boldsymbol{{\cal{C}}}}}}_{T}$. Namely, $\hat{{\bf{B}}}={[{\hat{{\boldsymbol{{\cal{C}}}}}}_{T,}]}_{:,1:m}$, where [**X**]_:,*i*:*j*_ indicates the sub-matrix of **X** obtained from keeping the entries from the *i*th to *j*th columns and all of its rows. An estimate of matrix **A** can be obtained as the solution to the least-squares problem
  17 $$\hat{{\bf{A}}}=\arg \mathop{\min }\limits_{{\bf{A}}}{\left\Vert {[{\hat{{\boldsymbol{{\cal{C}}}}}}_{T}]}_{:,m+1:mT}-{\bf{A}}\ {[{\hat{{\boldsymbol{{\cal{C}}}}}}_{T}]}_{:,1:(T-1)m}\right\Vert }_{\,\text{F}\,}^{2},$$
  which yields the matrix $\hat{{\bf{A}}}={[{\hat{{\boldsymbol{{\cal{C}}}}}}_{T}]}_{:,m+1:mT}{[{\hat{{\boldsymbol{{\cal{C}}}}}}_{T}]}_{:,1:(T-1)m}^{\dagger }$.

If the data are noiseless, the system is controllable in *T* − 1 steps, and **U**_0:*T*−1_ has full row rank, then this procedure provably returns correct estimates of **A** and **B**^66^.

### Power-grid network dynamics, parameters, and data generation

The short-term electromechanical behavior of generators {2, …, 10} of the New England power-grid network are modeled by the swing equations^67^:

18 $${\dot{\delta }}_{i}= 	\;{\omega }_{i},\\ \frac{{H}_{i}}{\pi {f}_{b}}{\dot{\omega }}_{i}=	 \;-\!{D}_{i}{\omega }_{i}+{P}_{\text{m}i}-{G}_{ii}{E}_{i}^{2}+\mathop{\sum }\limits_{j=1,j\ne i}^{10}{E}_{i}{E}_{j}({G}_{ij}\cos ({\delta }_{i}-{\delta }_{j})+{B}_{ij}\sin ({\delta }_{i}-{\delta }_{j})).$$

where *δ*_*i*_ is the angular position or phase of the rotor in generator *i* with respect to generator 1, and where *ω*_*i*_ is the deviation of the rotor speed or frequency in generator *i* relative to the nominal angular frequency 2*πf*_*b*_. Generator 1 is assumed to be connected to an infinite bus and has constant phase and frequency. The parameters *H*_*i*_ and *D*_*i*_ are the inertia constant and damping coefficient, respectively, of generator *i*. The parameter *G*_*ii*_ is the internal conductance of generator *i*, and *G*_*ij*_ + i*B*_*ij*_ (where i is the imaginary unit) is the transfer impedance between generators *i* and *j*. The parameter *P*_m*i*_ denotes the mechanical input power of generator *i* and *E*_*i*_ denotes the internal voltage of generator *i*. The values of parameters *f*_*b*_, *H*_*i*_, *D*_*i*_, *G*_*ij*_, *B*_*ij*_, and *P*_m*i*_ in the non-faulty and faulty configuration are taken from ref. ^54^, while the voltages *E*_*i*_ and initial conditions (*δ*_*i*_(0), *ω*_*i*_(0) = 0) are fixed using a power-flow computation. In our numerical study, we discretize the dynamics in Eq. (18) using a forward Euler method with sampling time *T*_*s*_ = 2.5 × 10^−4^ s. Data are generated by applying a Gaussian i.i.d. perturbation with zero mean and variance 0.01 to each frequency *ω*_*i*_ of the swing dynamics in Eq. (18). The initial condition of each experiment is computed by adding a Gaussian i.i.d. perturbation with zero mean and variance 0.01 to the steady-state values of *δ*_*i*_ and *ω*_*i*_ of the swing dynamics in Eq. (18).

### Task-fMRI dataset, pre-processing pipeline, and identification setup

The motor task-fMRI data used in our numerical study are extracted from the HCP S1200 release^57,68^. The details for data acquisition and experiment design can be found in ref. ^68^. The BOLD measurements have been pre-processed according to the minimal pipeline described in ref. ^69^, and, as in ref. ^58^, filtered with a band-pass filter to attenuate the frequencies outside the 0.06–0.12 Hz band. Specifically, we use an order 50 FIR-type filter using Matlab^®^equiripple method, so as to achieve a 20 dB attenuation outside the passband. The initial stop and pass frequencies considered were *f*_*s*,1_ = 0.04 Hz, *f*_*p*,1_ = 0.06 Hz, and the final stop and pass frequencies were *f*_*p*,2_ = 0.12 Hz and *f*_*s*,2_ = 0.15 Hz, respectively. Further, as common practice, the effect of the physiological signals (cardiac, respiratory, and head motion signals) is removed from the BOLD measurements by means of standard regression procedure^58^. The data matrices in Eq. (3) are generated via a sliding window of fixed length *T* = 100 with initial time in the interval [−90, 10]. We assume that the inputs and states are zero for times less than or equal to 10, i.e., the instant at which the first task condition is issued. We approximate the input–output dynamics with a linear model with state dimension *n* = 20 computed using input–output data in the interval [0, 150] and the subspace-based identification procedure detailed in ref. ^58^. In particular, we use (Hankel) output data matrices with columns consisting of *s* = 3 output samples and a regularization term *γ***I**, *γ* = 5, in the regression procedure for the estimation of matrix **B**. When the estimated network matrix **A** has unstable eigenvalues, we stabilize **A** by diving it by *ρ*(**A**) + 0.01, where *ρ*(**A**) denotes the spectral radius of **A**.

### Additional computational and experimental details

All numerical simulations have been performed via standard linear-algebra LAPACK routines available as built-in functions in Matlab^®^ R2019b, running on a 2.6 GHz Intel Core i5 processor with 8 GB of RAM. In particular, for the computation of pseudoinverses, we use the singular value decomposition method (command pinv in Matlab^®^) with a threshold of 10^−8^. In the numerical simulations of Figs. 2 and 3, if not otherwise stated, the entries of the final state **y**_f_ and those of the input data matrix **U**_0:*T*−1_ are standard normal i.i.d. variables, the input/output nodes are randomly selected with the only constraint that the resulting system is output controllable, and the curves represent the average over 500 independent realizations of networks, data, input/output nodes, and final states. We ensure output controllability by choosing networks that are connected and by choosing sets of input/output nodes that yield the smallest singular value of the resulting output controllability matrix no smaller than 10^−10^.

## Supplementary information

Supplementary Information

Peer Review File

## Data Availability

The New England power-grid interconnection scheme can be found in Appendix A of the reference textbook ref. ^70^ and the grid parameters in the faulty and non-faulty configurations are described in ref. ^54^. The HCP data used in our study are part of the 1200 Subjects Release (S1200) and are publicly available on the ConnectomeDB database (https://db.humanconnectome.org). These data are also available in the public GitHub repository: https://github.com/baggiogi/data_driven_control.

## Code availability

The code used in this study is freely available in the public GitHub repository: https://github.com/baggiogi/data_driven_control.
