# Supplementary material for: Data-driven control of complex networks
Source: Nat Commun. 2021 Mar 3;12:1429. doi: 10.1038/s41467-021-21554-0 (PMC7930026; doi:10.1038/s41467-021-21554-0)
Supplement: Supplementary file 1 — Supplementary Information [file 41467_2021_21554_MOESM1_ESM.pdf]

Supplementary Information
Data-Driven Control of Complex Networks

Giacomo Baggio[1]

[1]*Department of Information Engineering,*

University of Padova, Padova, Italy

Danielle S. Bassett[2,3,4,5,6,7]

[2]*Department of Bioengineering, University of Pennsylvania, Philadelphia, USA*

[3]*Department of Physics & Astronomy, Electrical,*

University of Pennsylvania, Philadelphia, USA

[4]*Department of Electrical & Systems Engineering,*

University of Pennsylvania, Philadelphia, USA

[5]*Department of Neurology, University of Pennsylvania, Philadelphia, USA*

[6]*Department of Psychiatry, University of Pennsylvania, Philadelphia, USA*

[7]*Santa Fe Institute, Santa Fe, USA*

Fabio Pasqualetti[8]

[8]*Department of Mechanical Engineering,*

University of California at Riverside, Riverside, USA

CONTENTS

Supplementary Note 1: Output controllability of linear (network) systems 3

Supplementary Note 2: Optimal data-driven controls for arbitrary $\mathbf{Q} \succeq \mathbf{0}$ and $\mathbf{R} \succ \mathbf{0}$ 4

Supplementary Note 3: Minimum number of data to reconstruct $\mathbf{u}^{\star}_{0:T-1}$ 5

Supplementary Note 4: A data-based controllability condition 6

Supplementary Note 5: Extension to general experimental settings 6

Supplementary Note 6: Formulas for $\mathbf{Q} = \mathbf{0}$ and $\mathbf{R} = \mathbf{I}$ (minimum-energy input) 7

Supplementary Note 7: Approximate data-driven minimum-energy controls 9

Supplementary Note 8: Data-driven optimal control inputs with noisy data 11

Supplementary References 15

Supplementary Figures 16

SUPPLEMENTARY NOTE 1: OUTPUT CONTROLLABILITY OF LINEAR (NETWORK) SYSTEMS

Consider the linear time-invariant system

$$\begin{aligned} \mathbf{x}(t+1) &= \mathbf{A}\mathbf{x}(t) + \mathbf{B}\mathbf{u}(t), \\ \mathbf{y}(t) &= \mathbf{C}\mathbf{x}(t), \end{aligned} \tag{1}$$

where $\mathbf{x}(t) \in \mathbb{R}^n$, $\mathbf{u}(t) \in \mathbb{R}^m$, and $\mathbf{y}(t) \in \mathbb{R}^p$ denote, respectively, the state, input, and output of the system at time t.

Definition 1 (Output controllable vectors). *An output vector $\mathbf{y}_\mathrm{f} \in \mathbb{R}^p$ is said to be output controllable in T steps if there exists a control sequence $\mathbf{u}(0), \mathbf{u}(1), \ldots, \mathbf{u}(T-1)$ that drives the output of* (1) *to $\mathbf{y}(T) = \mathbf{y}_\mathrm{f}$ assuming that $\mathbf{x}(0) = \mathbf{0}$. Further, $\mathbf{y}_\mathrm{f} \in \mathbb{R}^p$ is said to be output controllable if there exists a finite time $T > 0$ such that $\mathbf{y}_\mathrm{f}$ is output controllable in T steps.*

Definition 2 (Output controllability [1]). *The system* (1) *is said to be output controllable in T steps if every output vector $\mathbf{y}_\mathrm{f} \in \mathbb{R}^p$ is output controllable in T steps. Further,* (1) *is said to be output controllable if every output vector $\mathbf{y}_\mathrm{f} \in \mathbb{R}^p$ is output controllable.*

A few remarks are in order (see also Ref. [1]):

1. If $\mathbf{y}_\mathrm{f}$ is output controllabile in T steps, then it is so for any number $\bar{T} \geq T$ of steps. Similarly, if the system (1) is output controllable in $\bar{T}$ steps, then it is so for any number $\bar{T} \geq T$ of steps. Further, if the number of steps satisfies $T \geq n$, output controllability in T steps is equivalent to output controllability. We point out that it is possible for a vector or system to be output controllable in a finite number $\bar{T}$ of steps but not output controllable in $0 < T < \bar{T}$ steps. This a distinguishing feature of discrete-time systems, which does not have a counterpart in continuous time;

2. if (1) is output controllable (in T steps), then it is output controllable (in T steps) for any choice of the initial state, i.e., for any $\mathbf{x}(0) \in \mathbb{R}^n$;

3. output controllability can be assessed by computing the rank of the output controllability matrix of (1), $\boldsymbol{\mathcal{C}} = \begin{bmatrix}\mathbf{CB} & \mathbf{CAB} & \cdots & \mathbf{CA}^{n-1}\mathbf{B}\end{bmatrix}$. Namely, the system (1) is output controllable if and only if

$$\operatorname{rank}(\boldsymbol{\mathcal{C}}) = p\,; \tag{2}$$

4. if $\mathbf{C} = \mathbf{I}$ then output controllability reduces to the classic notion of controllability, and condition (2) to the classic Kalman's rank controllability condition [2]. Further, for full row rank $\mathbf{C}$'s, it follows from (2) that classic controllability implies output controllability, while the converse is not true in general. Thus, output controllability is typically less restrictive than classic (full) controllability.

Finally, we point out that if the system (1) describes the dynamics of a network with adjacency matrix $\mathbf{A}$ and the output matrix $\mathbf{C}$ is chosen to single out a subset of nodes of the network, then output controllability has been also termed target controllability in a number of recent works [3, 4]. It is worth remarking that, if self-loops are allowed and the network is connected, then controllability is generically satisfied for almost all choices of the network weights, independently of the location of the control nodes [5–7]. Thus, the definition of output controllability, which is less stringent than classic controllability, is mild and, in fact, generically satisfied for a large class of network systems.

SUPPLEMENTARY NOTE 2: OPTIMAL DATA-DRIVEN CONTROLS FOR ARBITRARY $\mathbf{Q} \succeq \mathbf{0}$ AND $\mathbf{R} \succ \mathbf{0}$

Consider the data matrices $\mathbf{U}_{0:T-1}$, $\mathbf{Y}_{1:T-1}$, $\mathbf{Y}_T$ as defined in Eq. (4) of the main text. By linearity of the system, a linear combination of the input experiments (columns of $\mathbf{U}_{0:T-1}$) yields an output that is a linear combination (with the same coefficients) of the corresponding output data; that is, for any vector $\boldsymbol{\alpha} \in \mathbb{R}^N$, the input $\mathbf{u}_{0:T} = \mathbf{U}_{0:T}\boldsymbol{\alpha}$ generates the outputs $\mathbf{y}_{1:T-1} = \mathbf{Y}_{1:T-1}\boldsymbol{\alpha}$ and $\mathbf{y}_T = \mathbf{Y}_T\boldsymbol{\alpha}$. Assume that there exists a vector $\boldsymbol{\alpha}^\star$ such that

$$\mathbf{u}^\star_{0:T-1} = \mathbf{U}_{0:T-1}\boldsymbol{\alpha}^\star \tag{3}$$

is the optimal (w.r.t. the cost function in Eq. (2) of the main text) control reaching $\mathbf{y}_\mathrm{f}$ in T steps. Then, $\boldsymbol{\alpha}^\star$ satisfies

$$\begin{aligned} \boldsymbol{\alpha}^\star = \arg\min_{\boldsymbol{\alpha}} \quad & \|\mathbf{L}\boldsymbol{\alpha}\|_2^2 \\ \text{s.t. } \; & \mathbf{y}_\mathrm{f} = \mathbf{Y}_T\boldsymbol{\alpha}. \end{aligned} \tag{4}$$

where $\mathbf{L}$ is any matrix satisfying $\mathbf{L}^\top\mathbf{L} = \mathbf{Y}^\mathsf{T}_{1:T-1}\,\mathbf{Q}\,\mathbf{Y}_{1:T-1} + \mathbf{U}^\mathsf{T}_{0:T-1}\,\mathbf{R}\,\mathbf{U}_{0:T-1}$[1] and $\|\cdot\|_2^2$ denotes the 2-norm of a vector. From the constraint $\mathbf{y}_\mathrm{f} = \mathbf{Y}_T\boldsymbol{\alpha}$, the optimal $\boldsymbol{\alpha}^*$ has the

[1] Notice that such a matrix $\mathbf{L}$ always exists since $\mathbf{Q} \succeq \mathbf{0}$ and $\mathbf{R} \succ \mathbf{0}$ imply that $\mathbf{Y}^\mathsf{T}_{1:T-1}\,\mathbf{Q}\,\mathbf{Y}_{1:T-1} + \mathbf{U}^\mathsf{T}_{0:T-1}\,\mathbf{R}\,\mathbf{U}_{0:T-1} \succeq \mathbf{0}$.

form $\boldsymbol{\alpha}^\star = \mathbf{Y}_T^\dagger \mathbf{y}_\mathrm{f} + \mathbf{K}_{\mathbf{Y}_T}\mathbf{w}^\star$, with $\mathbf{w}^\star$ being any vector such that

$$\mathbf{w}^\star = \arg\min_{\mathbf{w}} \quad \left\| \mathbf{L}(\mathbf{Y}_T^\dagger \mathbf{y}_\mathrm{f} + \mathbf{K}_{\mathbf{Y}_T}\mathbf{w}) \right\|_2^2, \tag{5}$$

where $\mathbf{K}_{\mathbf{Y}_T}$ denotes a matrix whose columns form a basis of the kernel of $\mathbf{Y}_T$. The solutions to the latter problem are of the form $\mathbf{w}^\star = -(\mathbf{L}\mathbf{K}_{\mathbf{Y}_T})^\dagger \mathbf{L}\mathbf{Y}_T^\dagger \mathbf{y}_\mathrm{f} + \mathbf{v}$, where $\mathbf{v} \in \mathrm{Ker}(\mathbf{L}\mathbf{K}_{\mathbf{Y}_T})$. Taking $\mathbf{v} = \mathbf{0}$ yields $\boldsymbol{\alpha}^\star = (\mathbf{I} - \mathbf{K}_{\mathbf{Y}_T}(\mathbf{L}\mathbf{K}_{\mathbf{Y}_T})^\dagger \mathbf{L})\mathbf{Y}_T^\dagger \mathbf{y}_\mathrm{f}$, so that the optimal control $\mathbf{u}^\star_{0:T-1}$ to reach $\mathbf{y}_\mathrm{f}$ in T steps (that is, (3)) can be written as

$$\mathbf{u}^\star_{0:T-1} = \mathbf{U}_{0:T-1}\boldsymbol{\alpha}^\star = \mathbf{U}_{0:T-1}(\mathbf{I} - \mathbf{K}_{\mathbf{Y}_T}(\mathbf{L}\mathbf{K}_{\mathbf{Y}_T})^\dagger \mathbf{L})\mathbf{Y}_T^\dagger \mathbf{y}_\mathrm{f}. \tag{6}$$

Finally, observe that any other choice of $\mathbf{v} \in \mathrm{Ker}(\mathbf{L}\mathbf{K}_{\mathbf{Y}_T})$ does not alter the above expression. Indeed, $\mathbf{L}\mathbf{K}_{\mathbf{Y}_T}\mathbf{v} = \mathbf{0}$ implies $\mathbf{U}_{0:T-1}\mathbf{K}_{\mathbf{Y}_T}\mathbf{v} = \mathbf{0}$ since $\mathbf{R} \succ 0$.

SUPPLEMENTARY NOTE 3: MINIMUM NUMBER OF DATA TO RECONSTRUCT $\mathbf{u}^\star_{0:T-1}$

If the columns of $\mathbf{U}_{0:T-1}$ span the space of all possible input sequences $\mathbb{R}^{mT}$, that is $\mathbf{U}_{0:T-1}$ is full row rank, then there always exists a vector $\boldsymbol{\alpha}^\star$ satisfying $\mathbf{u}^\star_{0:T-1} = \mathbf{U}_{0:T-1}\boldsymbol{\alpha}^\star$, for any T-steps output controllable target $\mathbf{y}_\mathrm{f}$. Hence, mT linearly independent input data are sufficient to reconstruct the optimal control via (6). Thus, the minimum number of data to reconstruct the optimal control $\mathbf{u}^\star_{0:T-1}$ satisfies $N_{\min} \leq mT$ if the control experiments are linearly independent (possibly, random). Further, we observe that if $\mathbf{y}_\mathrm{f}$ belongs to the range space of $\mathbf{Y}_T$, i.e., $\mathbf{y}_\mathrm{f} \in \mathrm{Im}(\mathbf{Y}_T)$, then the vector $\boldsymbol{\alpha}^\star$ in (4) satisfies the constraint $\mathbf{y}_\mathrm{f} = \mathbf{Y}_T\boldsymbol{\alpha}^\star$, so that the resulting control (6), although in general suboptimal, still correctly steers the output to the desired target $\mathbf{y}_\mathrm{f}$.[2] We remark that, for a generic (T-steps output controllable) target $\mathbf{y}_\mathrm{f}$, p randomly generated control experiments normally suffice for $\mathbf{y}_\mathrm{f} \in \mathrm{Im}(\mathbf{Y}_T)$. In fact, the output measurements $\mathbf{y}_T^{(i)} = \boldsymbol{\mathcal{C}}_T\mathbf{u}^{(i)}_{0:T-1}$ are linearly independent with probability one, if the input sequences $\mathbf{u}^{(i)}_{0:T-1}$ are generated randomly. This follows from the fact that the kernel of the T-steps output controllability matrix $\boldsymbol{\mathcal{C}}_T \in \mathbb{R}^{p\times mT}$ has zero (Lebesgue) measure in $\mathbb{R}^{mT}$ being a $k < mT$ dimensional linear subspace of $\mathbb{R}^{mT}$.

[2] Clearly, if $\mathbf{y}_\mathrm{f} \in \mathrm{Im}(\mathbf{Y}_T)$ and $\mathbf{U}_{0:T-1}$ comprises optimal experiments, then (6) yields the optimal control input, since any linear combination of optimal control inputs is still an optimal control input.

SUPPLEMENTARY NOTE 4: A DATA-BASED CONTROLLABILITY CONDITION

Building on the results of the previous section (Supplementary Note 3), if $\mathbf{Y}_T$ has full row rank then any output $\mathbf{y}_\mathrm{f} \in \mathbb{R}^p$ can be reached in T steps using an input sequence which is a linear combination of the available input data. This implies that the system is output controllable in T steps if $\mathbf{Y}_T$ has full row rank. If $N \geq mT$ linearly independent control experiments are available, then the converse is also true because every possible input sequence $\mathbf{u}_{0:T-1} \in \mathbb{R}^{mT}$ can be reconstructed from data as a linear combination of the columns of $\mathbf{U}_{0:T-1}$. Finally, we observe that for $T \geq n$, T-steps output controllability is equivalent to output controllability (cf. Supplementary Note 1). Thus, if $N \geq mT$ linearly independent control experiments are collected with $T \geq n$, then the system is output controllable if and only if $\mathbf{Y}_T$ has full row rank.

SUPPLEMENTARY NOTE 5: EXTENSION TO GENERAL EXPERIMENTAL SETTINGS

When the initial state of the network is different from $\mathbf{x}(0) = \mathbf{0}$ across the experiments, then the optimal control input can be still reconstructed from data, provided that the initial state of each experiment can be measured. In this case, data may consist of a single uninterrupted input/output trajectory of the system, where different control experiments correspond to different segments of length T of this trajectory.

Let $\mathbf{X}_0 = \begin{bmatrix} \mathbf{x}_0^{(1)} & \mathbf{x}_0^{(2)} & \cdots & \mathbf{x}_0^{(N)} \end{bmatrix} \in \mathbb{R}^{n \times N}$ denote the matrix whose columns consist of the initial states of all control experiments, and $\mathbf{K}_{\mathbf{X}_0}$ denote a basis of $\mathrm{Ker}(\mathbf{X}_0)$. Note that an input sequence $\mathbf{u}_{0:T-1}$ expressed as a linear combination of the columns of $\mathbf{U}_{0:T-1}\mathbf{K}_{\mathbf{X}_0}$ yields an output trajectory $\mathbf{y}_{1:T}$ which is a linear combination (with the same coefficients) of the columns of $\mathbf{Y}_{1:T}\mathbf{K}_{\mathbf{X}_0}$. Indeed, for any $\boldsymbol{\alpha} \in \mathbb{R}^d$, $d = \dim \mathrm{Ker}(\mathbf{X}_0)$, because of linearity of the system, we can rewrite the output data $\mathbf{Y}_T$ as a sum of a term depending only on $\mathbf{X}_0$ (free response) and a term depending only on $\mathbf{U}_{0:T-1}$ (forced response), so that

$$\mathbf{Y}_{1:T}\mathbf{K}_{\mathbf{X}_0}\boldsymbol{\alpha} = (\mathbf{G}\mathbf{X}_0 + \mathbf{H}\mathbf{U}_{0:T-1})\mathbf{K}_{\mathbf{X}_0}\boldsymbol{\alpha} = \mathbf{H}\mathbf{U}_{0:T-1}\mathbf{K}_{\mathbf{X}_0}\boldsymbol{\alpha}, \tag{7}$$

where $\mathbf{G}$, $\mathbf{H}$ are matrices of appropriate dimensions that depend on network matrices $\mathbf{A}$, $\mathbf{B}$,

C. Thus, assuming that there exists a vector $\boldsymbol{\alpha}^\star$ such that $\mathbf{u}^\star_{0:T-1} = \mathbf{U}_{0:T-1}\mathbf{K}_{\mathbf{X}_0}\boldsymbol{\alpha}^\star$ is the optimal control reaching $\mathbf{y}_\mathrm{f}$ in T steps, it holds that

$$\begin{aligned}\boldsymbol{\alpha}^\star = \arg\min_{\boldsymbol{\alpha}} \quad & \|\mathbf{L}\mathbf{K}_{\mathbf{X}_0}\boldsymbol{\alpha}\|_2^2 \\ \text{s.t. } & \mathbf{y}_\mathrm{f} = \mathbf{Y}_T\mathbf{K}_{\mathbf{X}_0}\boldsymbol{\alpha}.\end{aligned} \tag{8}$$

Hence, along the same lines of Supplementary Note 2, the optimal control input $\mathbf{u}^\star_{0:T-1}$ to reach $\mathbf{y}_\mathrm{f}$ in T steps is given by

$$\mathbf{u}^\star_{0:T-1} = \mathbf{U}_{0:T-1}\mathbf{K}_{\mathbf{X}_0}(\mathbf{I} - \mathbf{K}_{\mathbf{Y}_T\mathbf{K}_{\mathbf{X}_0}}(\mathbf{L}\mathbf{K}_{\mathbf{Y}_T\mathbf{K}_{\mathbf{X}_0}})^\dagger\mathbf{L})(\mathbf{Y}_T\mathbf{K}_{\mathbf{X}_0})^\dagger\mathbf{y}_\mathrm{f}, \tag{9}$$

where $\mathbf{K}_{\mathbf{Y}_T\mathbf{K}_{\mathbf{X}_0}}$ is a matrix whose columns form a basis of $\mathrm{Ker}(\mathbf{Y}_T\mathbf{K}_{\mathbf{X}_0})$. Notice that, if $\mathbf{U}_{0:T-1}\mathbf{K}_{\mathbf{X}_0}$ is full row rank, then there always exists a vector $\boldsymbol{\alpha}^\star$ such that $\mathbf{u}^\star_{0:T-1} = \mathbf{U}_{0:T-1}\mathbf{K}_{\mathbf{X}_0}\boldsymbol{\alpha}^\star$, for any target $\mathbf{y}_\mathrm{f}$. A sufficient (yet not necessary) condition for $\mathbf{U}_{0:T-1}\mathbf{K}_{\mathbf{X}_0}$ to be full row rank is that $[\mathbf{U}^\mathsf{T}_{0:T-1}\ \mathbf{X}^\mathsf{T}_0]^\mathsf{T}$ is full row rank.[3] This in turn implies that (at most) $mT + n$ linearly independent experiments (w.r.t. to inputs and initial states) suffice to reconstruct the optimal control via (9).

SUPPLEMENTARY NOTE 6: FORMULAS FOR $\mathbf{Q} = \mathbf{0}$ AND $\mathbf{R} = \mathbf{I}$ (MINIMUM-ENERGY INPUT)

For $\mathbf{Q} = \mathbf{0}$ and $\mathbf{R} = \mathbf{I}$, we can take $\mathbf{L} = \mathbf{Z}\mathbf{U}_{0:T-1}$, where $\mathbf{Z} \in \mathbb{R}^{mT\times mT}$ is an arbitrary orthogonal matrix, and the data-driven expression in (6) becomes

$$\mathbf{u}^\star_{0:T-1} = (\mathbf{I} - \mathbf{U}_{0:T-1}\mathbf{K}_{\mathbf{Y}_T}(\mathbf{U}_{0:T-1}\mathbf{K}_{\mathbf{Y}_T})^\dagger)\mathbf{U}_{0:T-1}\mathbf{Y}_T^\dagger\mathbf{y}_\mathrm{f}. \tag{10}$$

(10) equals the minimum-energy control input to reach $\mathbf{y}_\mathrm{f}$ in T steps [2], if mT linearly independent input experiments are collected. The expression in (10) can be further simplified by exploiting the following instrumental result.

Lemma 1. *Let* $\mathbf{A} \in \mathbb{R}^{r\times n}$, $\mathbf{B} \in \mathbb{R}^{q\times n}$. *If* $\mathrm{Ker}(\mathbf{A}) \subseteq \mathrm{Ker}(\mathbf{B})$, *then* $(\mathbf{I} - \mathbf{A}\mathbf{K}_\mathbf{B}(\mathbf{A}\mathbf{K}_\mathbf{B})^\dagger)\mathbf{A}\mathbf{B}^\dagger = (\mathbf{B}\mathbf{A}^\dagger)^\dagger$, *where* $\mathbf{K}_\mathbf{B}$ *is a matrix whose columns are a basis of* $\mathrm{Ker}(\mathbf{B})$.

[3] Indeed, if $[\mathbf{U}^\mathsf{T}_{0:T-1}\ \mathbf{X}^\mathsf{T}_0]^\mathsf{T}$ is full row rank, for all $\mathbf{u} \in \mathbb{R}^{mT}$ there exists $\boldsymbol{\gamma} \in \mathrm{Ker}(\mathbf{X}_0)$ such that $[\mathbf{u}^\mathsf{T}\ \mathbf{0}^\mathsf{T}]^\mathsf{T} = [\mathbf{U}^\mathsf{T}_{0:T-1}\ \mathbf{X}^\mathsf{T}_0]^\mathsf{T}\boldsymbol{\gamma}$, which implies that $\mathbf{U}_{0:T-1}\mathbf{K}_{\mathbf{X}_0}$ must be of full row rank.

Proof. We show that $(\mathbf{I} - \mathbf{A}\mathbf{K_B}(\mathbf{A}\mathbf{K_B})^\dagger)\mathbf{A}\mathbf{B}^\dagger$ satisfies the four conditions [8] defining the Moore–Penrose pseudoinverse of $\mathbf{B}\mathbf{A}^\dagger$. To this aim, let $\mathbf{\Pi} := \mathbf{I} - \mathbf{A}\mathbf{K_B}(\mathbf{A}\mathbf{K_B})^\dagger$. By noticing that $\mathbf{\Pi} = \mathbf{\Pi}^\mathsf{T}$ is the orthogonal projection onto $\mathrm{Ker}((\mathbf{A}\mathbf{K_B})^\mathsf{T})$, we have

$$(\mathbf{A}\mathbf{K_B})^\mathsf{T}\mathbf{\Pi} = \mathbf{0} \implies \mathbf{\Pi}\mathbf{A}\mathbf{K_B} = \mathbf{0} \implies \mathbf{\Pi}\mathbf{A}\mathbf{B}^\dagger\mathbf{B} = \mathbf{\Pi}\mathbf{A}. \tag{11}$$

By assumption $\mathrm{Ker}(\mathbf{A}) \subseteq \mathrm{Ker}(\mathbf{B})$, which implies

$$\mathbf{B}(\mathbf{I} - \mathbf{A}^\dagger\mathbf{A}) = \mathbf{0} \implies \mathbf{B}\mathbf{A}^\dagger\mathbf{A} = \mathbf{B}, \tag{12}$$

since $\mathbf{I} - \mathbf{A}^\dagger\mathbf{A}$ is the orthogonal projection onto $\mathrm{Ker}(\mathbf{A})$. Further, since $\mathbf{B}\mathbf{K_B} = 0$, we have

$$\mathbf{B}\mathbf{A}^\dagger(\mathbf{I} - \mathbf{\Pi}) = \mathbf{B}\mathbf{A}^\dagger\mathbf{A}\mathbf{K_B}(\mathbf{A}\mathbf{K_B})^\dagger \overset{(12)}{=} \mathbf{B}\mathbf{K_B}(\mathbf{A}\mathbf{K_B})^\dagger = \mathbf{0}. \tag{13}$$

Finally, since $\mathbf{I} - \mathbf{A}\mathbf{A}^\dagger$ equals the orthogonal projection onto $\mathrm{Ker}(\mathbf{A}^\mathsf{T})$, and $\mathbf{I} - \mathbf{\Pi} = \mathbf{A}\mathbf{K_B}(\mathbf{A}\mathbf{K_B})^\dagger$ equals the orthogonal projection onto $\mathrm{Im}(\mathbf{A}\mathbf{K_B}) \subseteq \mathrm{Im}(\mathbf{A}) \perp \mathrm{Ker}(\mathbf{A}^\mathsf{T})$, where $\mathrm{Im}(\cdot)$ denotes the image or column space of a matrix, we have

$$(\mathbf{I} - \mathbf{\Pi})(\mathbf{I} - \mathbf{A}\mathbf{A}^\dagger) = [(\mathbf{I} - \mathbf{\Pi})(\mathbf{I} - \mathbf{A}\mathbf{A}^\dagger)]^\mathsf{T} = \mathbf{0} \implies \mathbf{A}\mathbf{A}^\dagger\mathbf{\Pi} = \mathbf{\Pi}\mathbf{A}\mathbf{A}^\dagger, \tag{14}$$

where the last implication follows because $\mathbf{I} - \mathbf{\Pi}$ and $\mathbf{I} - \mathbf{A}\mathbf{A}^\dagger$ are symmetric. To conclude, we show that $\mathbf{\Pi}\mathbf{A}\mathbf{B}^\dagger = (\mathbf{B}\mathbf{A}^\dagger)^\dagger$ by proving the four Moore–Penrose conditions [8]:

1. $\mathbf{\Pi}\mathbf{A}\mathbf{B}^\dagger\mathbf{B}\mathbf{A}^\dagger\mathbf{\Pi}\mathbf{A}\mathbf{B}^\dagger \overset{(11)}{=} \mathbf{\Pi}\mathbf{A}\mathbf{A}^\dagger\mathbf{\Pi}\mathbf{A}\mathbf{B}^\dagger \overset{(14)}{=} \mathbf{\Pi}^2\mathbf{A}\mathbf{A}^\dagger\mathbf{A}\mathbf{B}^\dagger = \mathbf{\Pi}\mathbf{A}\mathbf{B}^\dagger$;
2. $\mathbf{B}\mathbf{A}^\dagger\mathbf{\Pi}\mathbf{A}\mathbf{B}^\dagger\mathbf{B}\mathbf{A}^\dagger \overset{(11)}{=} \mathbf{B}\mathbf{A}^\dagger\mathbf{\Pi}\mathbf{A}\mathbf{A}^\dagger = \mathbf{B}\mathbf{A}^\dagger\mathbf{A}\mathbf{A}^\dagger - \mathbf{B}\mathbf{A}^\dagger(\mathbf{I} - \mathbf{\Pi})\mathbf{A}\mathbf{A}^\dagger \overset{(13)}{=} \mathbf{B}\mathbf{A}^\dagger$;
3. $\mathbf{B}\mathbf{A}^\dagger\mathbf{\Pi}\mathbf{A}\mathbf{B}^\dagger = \mathbf{B}\mathbf{A}^\dagger\mathbf{A}\mathbf{B}^\dagger - \mathbf{B}\mathbf{A}^\dagger(\mathbf{I} - \mathbf{\Pi})\mathbf{A}\mathbf{B}^\dagger \overset{(12),(13)}{=} \mathbf{B}\mathbf{B}^\dagger = (\mathbf{B}\mathbf{B}^\dagger)^\mathsf{T}$;
4. $\mathbf{\Pi}\mathbf{A}\mathbf{B}^\dagger\mathbf{B}\mathbf{A}^\dagger \overset{(11)}{=} \mathbf{\Pi}\mathbf{A}\mathbf{A}^\dagger \overset{(14)}{=} \mathbf{A}\mathbf{A}^\dagger\mathbf{\Pi} = (\mathbf{\Pi}\mathbf{A}\mathbf{A}^\dagger)^\mathsf{T}$.

This concludes the proof. □

Since $\mathbf{Y}_T = \boldsymbol{\mathcal{C}}_T\mathbf{U}_{0:T-1}$, where $\boldsymbol{\mathcal{C}}_T = [\mathbf{C}\mathbf{B}\ \mathbf{C}\mathbf{A}\mathbf{B}\ \cdots\ \mathbf{C}\mathbf{A}^{T-1}\mathbf{B}]$ is the T-steps output controllability matrix of the network, it holds that $\mathrm{Ker}(\mathbf{U}_{0:T-1}) \subseteq \mathrm{Ker}(\mathbf{Y}_T)$. Thus, by Lemma 1, (9) can be compactly rewritten as

$$\mathbf{u}^\star_{0:T-1} = (\mathbf{Y}_T\mathbf{U}^\dagger_{0:T-1})^\dagger\mathbf{y}_\mathrm{f}. \tag{15}$$

When the optimal input can be reconstructed from the available data, (15) could also be derived by "direct" estimation of the output controllability matrix $\boldsymbol{\mathcal{C}}_T$. However, we remark that, based on Lemma 1, the data-driven expressions in (9) and (15) are equivalent even when the optimal input cannot be reconstructed from the available data.

SUPPLEMENTARY NOTE 7: APPROXIMATE DATA-DRIVEN MINIMUM-ENERGY CONTROLS

Consider the data-driven control input

$$\tilde{\mathbf{u}}_{0:T-1} = \mathbf{U}_{0:T-1}\mathbf{Y}_T^{\dagger}\mathbf{y}_{\mathrm{f}}. \tag{16}$$

Notice that $\tilde{\mathbf{u}}_{0:T-1}$ correctly steers the network to $\mathbf{y}_{\mathrm{f}}$ in T steps, as long as $\mathbf{y}_{\mathrm{f}} \in \mathrm{Im}(\mathbf{Y}_T)$. Indeed, if $\mathbf{y}_{\mathrm{f}} \in \mathrm{Im}(\mathbf{Y}_T)$, then $\bar{\boldsymbol{\alpha}} := \mathbf{Y}_T^{\dagger}\mathbf{y}_{\mathrm{f}}$ satisfies $\mathbf{Y}_T\bar{\boldsymbol{\alpha}} = \mathbf{y}_{\mathrm{f}}$, so that $\tilde{\mathbf{u}}_{0:T-1} = \mathbf{U}_{0:T-1}\bar{\boldsymbol{\alpha}} = \mathbf{U}_{0:T-1}\mathbf{Y}_T^{\dagger}\mathbf{y}_{\mathrm{f}}$ drives the network to $\mathbf{y}_{\mathrm{f}}$. Following the arguments in Supplementary Note 2, we remark that p random input experiments normally suffice for $\mathbf{y}_{\mathrm{f}} \in \mathrm{Im}(\mathbf{Y}_T)$. Although $\tilde{\mathbf{u}}_{0:T-1}$ does not typically coincide with the minimum-energy control, when the input experiments are generated randomly and independently from a Gaussian distribution, $\tilde{\mathbf{u}}_{0:T-1}$ approaches the minimum-energy control as the number of experiments grows, as we show next. To this end, we need the following standard result in non-asymptotic random matrix theory, e.g., see [9, Corollary 5.35 and Lemma 5.36]. Given a matrix $\mathbf{X} \in \mathbb{R}^{n\times m}$, $\sigma_{\min}(\mathbf{A})$, $\sigma_{\max}(\mathbf{A})$, and $\kappa(\mathbf{A}) := \sigma_{\max}(\mathbf{A})/\sigma_{\min}(\mathbf{A})$ denote the largest, smallest (non-zero) singular value, and the condition number of $\mathbf{A}$, respectively.

Lemma 2. *Let $\mathbf{X} \in \mathbb{R}^{N\times q}$ have i.i.d. normally distributed entries. Then, with probability at least $1-\delta$*

$$\left\| \frac{1}{N}\mathbf{X}^{\mathsf{T}}\mathbf{X} - \mathbf{I} \right\|_2 \leq 3\max(\eta, \eta^2),$$

$$1-\eta \leq \sigma_{\min}\left(\frac{1}{\sqrt{N}}\mathbf{X}\right) \leq \sigma_{\max}\left(\frac{1}{\sqrt{N}}\mathbf{X}\right) \leq 1+\eta,$$

where $\eta := \sqrt{q/N} + \sqrt{2\ln(1/\delta)/N}$ and $\|\mathbf{X}\|_2 = \sigma_{\max}(\mathbf{X})$.

Theorem 3. *Assume that the network is output controllable, $\mathbf{U}_{0:T-1}$ has full row rank, and the entries of $\mathbf{U}_{0:T-1}$ are i.i.d. Gaussian random variables with zero mean and finite variance $\sigma^2 \neq 0$. Then, with probability at least $1-\delta$*

$$\|\mathbf{u}^{\star}_{0:T-1} - \tilde{\mathbf{u}}_{0:T-1}\|_2 \leq \frac{3\max(\eta,\eta^2)}{\sigma_{\min}(\boldsymbol{\mathcal{C}}_T)}\left(1 + \frac{1+\eta}{1-\eta}\kappa^2(\boldsymbol{\mathcal{C}}_T)\right)\|\mathbf{y}_{\mathrm{f}}\|_2\,, \tag{17}$$

where $\mathbf{u}^{\star}_{0:T-1}$ is the minimum-energy control input driving the network to $\mathbf{y}_{\mathrm{f}}$, the matrix $\boldsymbol{\mathcal{C}}_T = [\mathbf{CB}\ \mathbf{CAB}\ \cdots\ \mathbf{CA}^{T-1}\mathbf{B}]$ is the T-steps output controllability matrix of the network, and $\eta := \sqrt{mT/N} + \sqrt{2\ln(1/\delta)/N}$. In particular, as $N \to \infty$,

$$\tilde{\mathbf{u}}_{0:T-1} \xrightarrow{\text{a.s.}} \mathbf{u}^{\star}_{0:T-1}, \tag{18}$$

where $\xrightarrow{a.s.}$ *stands for almost sure convergence.*

Proof. Since $\mathbf{Y}_T = \boldsymbol{\mathcal{C}}_T \mathbf{U}_{0:T-1}$, the data-driven input in (16) can be written as

$$\tilde{\mathbf{u}}_{0:T-1} = \mathbf{U}_{0:T-1}(\boldsymbol{\mathcal{C}}_T \mathbf{U}_{0:T-1})^{\dagger}\mathbf{y}_{\mathrm{f}} = \frac{1}{\sigma^2 N}\mathbf{U}_{0:T-1}\mathbf{U}_{0:T-1}^{\mathsf{T}}\boldsymbol{\mathcal{C}}_T^{\mathsf{T}}\left(\frac{1}{\sigma^2 N}\boldsymbol{\mathcal{C}}_T\mathbf{U}_{0:T-1}\mathbf{U}_{0:T-1}^{\mathsf{T}}\boldsymbol{\mathcal{C}}_T^{\mathsf{T}}\right)^{-1}\mathbf{y}_{\mathrm{f}}, \tag{19}$$

where we used that $\mathbf{X}^{\dagger} = \mathbf{X}^{\mathsf{T}}(\mathbf{X}\mathbf{X}^{\mathsf{T}})^{-1}$, if $\mathbf{X}$ has full row rank, e.g., see [8]. By using the above expression and the fact that $\mathbf{u}_{0:T-1}^{\star} = \boldsymbol{\mathcal{C}}_T^{\dagger}\mathbf{y}_{\mathrm{f}}$ [2], we have

$$\mathbf{e} := \mathbf{u}_{0:T-1}^{\star} - \tilde{\mathbf{u}}_{0:T-1} = \left[\boldsymbol{\mathcal{C}}_T^{\dagger} - \frac{1}{\sigma^2 N}\mathbf{U}_{0:T-1}\mathbf{U}_{0:T-1}^{\mathsf{T}}\boldsymbol{\mathcal{C}}_T^{\mathsf{T}}\left(\frac{1}{\sigma^2 N}\boldsymbol{\mathcal{C}}_T\mathbf{U}_{0:T-1}\mathbf{U}_{0:T-1}^{\mathsf{T}}\boldsymbol{\mathcal{C}}_T^{\mathsf{T}}\right)^{-1}\right]\mathbf{y}_{\mathrm{f}}. \tag{20}$$

By defining the matrix $\mathbf{V}_{0:T-1} := \mathbf{U}_{0:T-1}\mathbf{U}_{0:T-1}^{\mathsf{T}} - \sigma^2 N\mathbf{I}$, the latter equation can be written as

$$\begin{aligned}
\mathbf{e} &= \left[\boldsymbol{\mathcal{C}}_T^{\dagger} - \frac{1}{\sigma^2 N}(\mathbf{V}_{0:T-1} + \sigma^2 N\mathbf{I})\boldsymbol{\mathcal{C}}_T^{\mathsf{T}}\left(\frac{1}{\sigma^2 N}\boldsymbol{\mathcal{C}}_T\mathbf{V}_{0:T-1}\boldsymbol{\mathcal{C}}_T^{\mathsf{T}} + \boldsymbol{\mathcal{C}}_T\boldsymbol{\mathcal{C}}_T^{\mathsf{T}}\right)^{-1}\right]\mathbf{y}_{\mathrm{f}} \\
&= \left[\boldsymbol{\mathcal{C}}_T^{\dagger} - \frac{1}{\sigma^2 N}(\mathbf{V}_{0:T-1} + \sigma^2 N\mathbf{I})\boldsymbol{\mathcal{C}}_T^{\mathsf{T}}\left((\boldsymbol{\mathcal{C}}_T\boldsymbol{\mathcal{C}}_T^{\mathsf{T}})^{-1} - \left(\frac{1}{\sigma^2 N}\boldsymbol{\mathcal{C}}_T\mathbf{V}_{0:T-1}\boldsymbol{\mathcal{C}}_T^{\mathsf{T}} + \boldsymbol{\mathcal{C}}_T\boldsymbol{\mathcal{C}}_T^{\mathsf{T}}\right)^{-1}\cdot\right.\right. \\
&\quad \left.\left.\cdot\ \frac{1}{\sigma^2 N}\boldsymbol{\mathcal{C}}_T\mathbf{V}_{0:T-1}\boldsymbol{\mathcal{C}}_T^{\mathsf{T}}(\boldsymbol{\mathcal{C}}_T\boldsymbol{\mathcal{C}}_T^{\mathsf{T}})^{-1}\right)\right]\mathbf{y}_{\mathrm{f}} \\
&= \left[-\mathbf{I} + \frac{1}{\sigma^2 N}\mathbf{U}_{0:T-1}\mathbf{U}_{0:T-1}^{\mathsf{T}}\boldsymbol{\mathcal{C}}_T^{\mathsf{T}}\left(\frac{1}{\sigma^2 N}\boldsymbol{\mathcal{C}}_T\mathbf{U}_{0:T-1}\mathbf{U}_{0:T-1}^{\mathsf{T}}\boldsymbol{\mathcal{C}}_T^{\mathsf{T}}\right)^{-1}\boldsymbol{\mathcal{C}}_T\right]\frac{1}{\sigma^2 N}\mathbf{V}_{0:T-1}\boldsymbol{\mathcal{C}}_T^{\dagger}\mathbf{y}_{\mathrm{f}},
\end{aligned} \tag{21}$$

where in the second step we used the matrix identity $(\mathbf{X}+\mathbf{Y})^{-1} = \mathbf{Y}^{-1} - (\mathbf{X}+\mathbf{Y})^{-1}\mathbf{X}\mathbf{Y}^{-1}$, which holds for square matrices $\mathbf{X}$, $\mathbf{Y}$ with $\mathbf{Y}$ and $\mathbf{X}+\mathbf{Y}$ being non-singular (e.g., see [10, p. 151]), and in the last step the identity $\boldsymbol{\mathcal{C}}_T^{\dagger} = \boldsymbol{\mathcal{C}}_T^{\mathsf{T}}(\boldsymbol{\mathcal{C}}_T\boldsymbol{\mathcal{C}}_T^{\mathsf{T}})^{-1}$ which follows from the fact that $\boldsymbol{\mathcal{C}}_T$ has full row rank because the network is output controllable by assumption. Thus, from (21), the triangle inequality and the sub-multiplicativity of the 2-norm:

$$\begin{aligned}
\|\mathbf{e}\|_2 &\leq \left(1 + \left\|\frac{1}{\sigma^2 N}\mathbf{U}_{0:T-1}\mathbf{U}_{0:T-1}^{\mathsf{T}}\boldsymbol{\mathcal{C}}_T^{\mathsf{T}}\left(\frac{1}{\sigma^2 N}\boldsymbol{\mathcal{C}}_T\mathbf{U}_{0:T-1}\mathbf{U}_{0:T-1}^{\mathsf{T}}\boldsymbol{\mathcal{C}}_T^{\mathsf{T}}\right)^{-1}\boldsymbol{\mathcal{C}}_T\right\|_2\right)\left\|\frac{1}{\sigma^2 N}\mathbf{V}_{0:T-1}\boldsymbol{\mathcal{C}}_T^{\dagger}\mathbf{y}_{\mathrm{f}}\right\|_2 \\
&\leq \left(1 + \left\|\frac{1}{\sigma^2 N}\mathbf{U}_{0:T-1}\mathbf{U}_{0:T-1}^{\mathsf{T}}\right\|_2\|\boldsymbol{\mathcal{C}}_T\|_2^2\left\|\left(\frac{1}{\sigma^2 N}\boldsymbol{\mathcal{C}}_T\mathbf{U}_{0:T-1}\mathbf{U}_{0:T-1}^{\mathsf{T}}\boldsymbol{\mathcal{C}}_T^{\mathsf{T}}\right)^{-1}\right\|_2\right)\cdot \\
&\quad \cdot \left\|\frac{1}{\sigma^2 N}\mathbf{V}_{0:T-1}\right\|_2\left\|\boldsymbol{\mathcal{C}}_T^{\dagger}\right\|_2\|\mathbf{y}_{\mathrm{f}}\|_2 \\
&\leq \left(1 + \frac{\sigma_{\max}\left(\frac{1}{\sqrt{\sigma^2 N}}\mathbf{U}_{0:T-1}\right)\sigma_{\max}^2(\boldsymbol{\mathcal{C}}_T)}{\sigma_{\min}\left(\frac{1}{\sqrt{\sigma^2 N}}\mathbf{U}_{0:T-1}\right)\sigma_{\min}^2(\boldsymbol{\mathcal{C}}_T)}\right)\frac{\left\|\frac{1}{\sigma^2 N}\mathbf{V}_{0:T-1}\right\|_2}{\sigma_{\min}(\boldsymbol{\mathcal{C}}_T)}\|\mathbf{y}_{\mathrm{f}}\|_2,
\end{aligned} \tag{22}$$

where in the last step we used that $\sigma_{\min}(\mathbf{X}\mathbf{Y}) \geq \sigma_{\min}(\mathbf{X})\sigma_{\min}(\mathbf{Y})$[4] and $\|\mathbf{X}^{\dagger}\|_2 = \sigma_{\min}^{-1}(\mathbf{X})$, for matrices $\mathbf{X}$, $\mathbf{Y}$ of full row rank. The result now follows from (22), by invoking Lemma 2. $\square$

From the non-asymptotic bound in (17) of Theorem 4, for a fixed number N of i.i.d. Gaussian data, the larger $\sigma_{\min}(\boldsymbol{\mathcal{C}}_T)$ is, the closer the data-driven input in (16) to the minimum-energy one is. Since $\sigma_{\min}^{-1}(\boldsymbol{\mathcal{C}}_T)$ equals the worst-case control energy required to reach a unit-norm target [11], it follows that networks that are "easy" to control (i.e., networks featuring a large $\sigma_{\min}(\boldsymbol{\mathcal{C}}_T)$) yield the most favorable approximation performance. In other words, the more "excitable" [12] the network dynamics (i.e., the larger $\sigma_{\min}(\boldsymbol{\mathcal{C}}_T)$) are, the lower the approximation error is.

SUPPLEMENTARY NOTE 8: DATA-DRIVEN OPTIMAL CONTROL INPUTS WITH NOISY DATA

Data corrupted by small noise

Consider the minimum-energy data-driven expressions (15), (16), and assume that the data matrices $\mathbf{U}_{0:T-1}$, $\mathbf{Y}_T$ have full (row) rank. Since the Moore–Penrose pseudoinverse of a full (row or column) rank matrix $\mathbf{X}$ is a continuous function of the entries of $\mathbf{X}$ (in the set of matrices preserving the rank of $\mathbf{X}$) [8, Ch. 6], it follows that (15) and (16) are continuous functions of the data matrices around their true values. Thus, small perturbations of the entries of $\mathbf{U}_{0:T-1}$, $\mathbf{Y}_T$, yield a small deviation of the data-driven expressions (15) and (16) from their correct values. A similar argument applies to the optimal data-driven control (6), provided that the singular values of the pseudoinverse of $\mathbf{L}\mathbf{K}_{\mathbf{Y}_T}$ (which is not typically of full rank) are truncated by small constant $\varepsilon > 0$ to preserve the rank of $\mathbf{L}\mathbf{K}_{\mathbf{Y}_T}$ when small perturbations are applied to the data matrices $\mathbf{Y}_{1:T-1}$ and $\mathbf{Y}_T$.

[4] Indeed, if $\mathbf{X}$, $\mathbf{Y}$ have full row rank, it holds $\sigma_{\min}(\mathbf{X}\mathbf{Y}) = \lambda_{\min}(\mathbf{X}\mathbf{Y}\mathbf{Y}^{\mathsf{T}}\mathbf{X}^{\mathsf{T}})^{1/2} \geq \lambda_{\min}(\mathbf{Y}\mathbf{Y}^{\mathsf{T}})^{1/2}\lambda_{\min}(\mathbf{X}\mathbf{X}^{\mathsf{T}})^{1/2} = \sigma_{\min}(\mathbf{X})\sigma_{\min}(\mathbf{Y})$, where $\lambda_{\min}(\cdot)$ denotes the smallest eigenvalue of a symmetric matrix and we used that $\mathbf{P} \succeq \lambda_{\min}(\mathbf{P})\mathbf{I}$ if $\mathbf{P} \succeq \mathbf{0}$.

Data corrupted by i.i.d. noise with zero mean and known variance

We assume that the data matrices $\mathbf{U}_{0:T-1}$, $\mathbf{Y}_{1:T-1}$, $\mathbf{Y}_T$ are corrupted by i.i.d. noise with zero mean and finite variance. Namely, we consider the following dataset

$$\begin{aligned}\mathbf{U}_{0:T-1} &= \bar{\mathbf{U}}_{0:T-1} + \mathbf{\Delta}_{\mathbf{U}},\\ \mathbf{Y}_{1:T-1} &= \bar{\mathbf{Y}}_{1:T-1} + \mathbf{\Delta}_{\mathbf{Y}},\\ \mathbf{Y}_T &= \bar{\mathbf{Y}}_T + \mathbf{\Delta}_{\mathbf{Y}_T},\end{aligned} \tag{23}$$

where $\bar{\mathbf{U}}_{0:T-1}$, $\bar{\mathbf{Y}}_{1:T-1}$, and $\bar{\mathbf{Y}}_T$ denote the ground truth values, whereas $\mathbf{\Delta}_{\mathbf{U}}$, $\mathbf{\Delta}_{\mathbf{Y}}$, and $\mathbf{\Delta}_{\mathbf{Y}_T}$ are independent random matrices with i.i.d. entries with zero mean and variance $\sigma^2_{\mathbf{U}}$, $\sigma^2_{\mathbf{Y}}$, and $\sigma^2_{\mathbf{Y}_T}$, respectively.

The data-driven controls in (6), (10), (15), and (16) computed from the noisy data in (23) are typically biased and do not converge to the true control input as the data size N grows to infinity. For a concrete example of the latter fact, consider the approximate data-driven control in (16), the scalar ($p = n = m = 1$) system $x(t+1) = ax(t) + u(t)$, $y(t) = x(t)$, and a unitary control horizon ($T = 1$). In this simple scenario, (16) simplifies to

$$\hat{u}_0 = \frac{\sum_{i=1}^N u_1^{(i)} y_1^{(i)}}{\sum_{i=1}^N (y_1^{(i)})^2} = \frac{\sum_{i=1}^N (\bar{u}_1^{(i)} + \delta_{\mathbf{U}}^{(i)})(\bar{y}_1^{(i)} + \delta_{\mathbf{Y}_1}^{(i)})}{\sum_{i=1}^N (\bar{y}_1^{(i)} + \delta_{\mathbf{Y}_1}^{(i)})^2}, \tag{24}$$

where $\bar{\mathbf{U}}_0 = [\bar{u}_0^{(1)} \cdots \bar{u}_0^{(1)}]$, $\bar{\mathbf{Y}}_1 = [\bar{y}_1^{(1)} \cdots \bar{y}_1^{(N)}]$, and $\mathbf{\Delta}_{\mathbf{U}} = [\delta_{\mathbf{U}}^{(1)} \cdots \delta_{\mathbf{U}}^{(N)}]$, $\mathbf{\Delta}_{\mathbf{Y}_1} = [\delta_{\mathbf{Y}_1}^{(1)} \cdots \delta_{\mathbf{Y}_1}^{(N)}]$ denote the true data and noise samples, respectively. By the Strong Law of Large Numbers [13] and the assumption on the noise, as $N \to \infty$, it follows that

$$\hat{u}_0 = \frac{\frac{1}{N}\sum_{i=1}^N (\bar{u}_1^{(i)} + \delta_{\mathbf{U}}^{(i)})(\bar{y}_1^{(i)} + \delta_{\mathbf{Y}_1}^{(i)})}{\frac{1}{N}\sum_{i=1}^N (\bar{y}_1^{(i)} + \delta_{\mathbf{Y}_1}^{(i)})^2} \xrightarrow{\text{a.s.}} \frac{\frac{1}{N}\sum_{i=1}^N \bar{u}_1^{(i)}\bar{y}_1^{(i)}}{\frac{1}{N}\sum_{i=1}^N (\bar{y}_1^{(i)})^2 + \sigma^2_{\mathbf{Y}_1}}. \tag{25}$$

Because of the variance term $\sigma^2_{\mathbf{Y}_1}$ in the denominator, $\hat{u}_0$ does not converge to the noiseless control input. To remedy this situation, as an alternative to "denoising" data, one could modify the data-driven expressions in order to compensate for the variance of the noise, as we detail next.

We first consider the data-driven control in (6) and rewrite it as

$$\begin{aligned}\hat{\mathbf{u}}_{0:T-1} &= \mathbf{U}_{0:T-1}\left(\mathbf{I} - \mathbf{K}_{\mathbf{Y}_T}\left(\mathbf{L}\mathbf{K}_{\mathbf{Y}_T}\right)^{\dagger}\mathbf{L}\right)\mathbf{Y}_T^{\dagger}\mathbf{y}_{\mathrm{f}}\\ &= \mathbf{U}_{0:T-1}\left(\mathbf{I} - \mathbf{\Pi}_{\mathbf{Y}_T}\mathbf{L}^{\mathsf{T}}\left(\mathbf{L}\mathbf{\Pi}_{\mathbf{Y}_T}\mathbf{L}^{\mathsf{T}}\right)^{\dagger}\mathbf{L}\right)\mathbf{Y}_T^{\mathsf{T}}(\mathbf{Y}_T\mathbf{Y}_T^{\mathsf{T}})^{\dagger}\mathbf{y}_{\mathrm{f}},\end{aligned} \tag{26}$$

where $\mathbf{\Pi}_{\mathbf{Y}_T} = \mathbf{K}_{\mathbf{Y}_T}\mathbf{K}_{\mathbf{Y}_T}^\mathsf{T} = \mathbf{I} - \mathbf{Y}_T^\dagger\mathbf{Y}_T = \mathbf{I} - \mathbf{Y}_T^\mathsf{T}(\mathbf{Y}_T\mathbf{Y}_T^\mathsf{T})^\dagger\mathbf{Y}_T$ denote the orthogonal projection onto $\mathrm{Ker}(\mathbf{Y}_T)$ and we used that $\mathbf{X}^\dagger = \mathbf{X}^\mathsf{T}(\mathbf{X}\mathbf{X}^\mathsf{T})^\dagger$, for any matrix $\mathbf{X}$, e.g., see [8]. Next, we consider the following "corrected" version of (26)

$$\hat{\mathbf{u}}_{0:T-1}^{(\mathrm{c})} = \mathbf{U}_{0:T-1}\left(\mathbf{I} - \tilde{\mathbf{\Pi}}_{\mathbf{Y}_T}\mathbf{L}^\mathsf{T}\left(\mathbf{L}\tilde{\mathbf{\Pi}}_{\mathbf{Y}_T}\mathbf{L}^\mathsf{T} - \begin{bmatrix} N\sigma_{\mathbf{Y}}^2\mathbf{Q} & 0 \\ 0 & N\sigma_{\mathbf{U}}^2\mathbf{R}\end{bmatrix}\right)_\varepsilon^\dagger \mathbf{L}\right)\mathbf{Y}_T^\mathsf{T}(\mathbf{Y}_T\mathbf{Y}_T^\mathsf{T} - N\sigma_{\mathbf{Y}_T}^2\mathbf{I})^\dagger\mathbf{y}_\mathrm{f}, \tag{27}$$

where $\tilde{\mathbf{\Pi}}_{Y_T} := \mathbf{I} - \mathbf{Y}_T^\mathsf{T}(\mathbf{Y}_T\mathbf{Y}_T^\mathsf{T} - N\sigma_{\mathbf{Y}_T}^2\mathbf{I})^\dagger\mathbf{Y}_T$, $\mathbf{L}$ is the particular square root

$$\mathbf{L} := \begin{bmatrix}\mathbf{Q}^{1/2}\mathbf{Y}_{1:T-1} \\ \mathbf{R}^{1/2}\mathbf{U}_{0:T-1}\end{bmatrix}, \tag{28}$$

and $\mathbf{X}_\varepsilon^\dagger$ denotes the Moore–Penrose pseudoinverse of $\mathbf{X}$ that treats as zero the singular values of $\mathbf{X}$ that are smaller than $\varepsilon > 0$.

Theorem 4. *Consider the noisy dataset as in* (23) *and assume that* $\bar{\mathbf{U}}_{0:T-1}$ *has full row rank. For* $\varepsilon > 0$ *sufficiently small and* $N \to \infty$*, the control sequence in* (27) *converges almost surely to the optimal control input; that is,*

$$\hat{\mathbf{u}}_{0:T-1}^{(\mathrm{c})} \xrightarrow{\text{a.s.}} \mathbf{u}_{0:T-1}^\star. \tag{29}$$

Proof. After some algebraic manipulations, (27) can be written as

$$\begin{aligned}
\hat{\mathbf{u}}_{0:T-1}^{(\mathrm{c})} &= \mathbf{U}_{0:T-1}\left(\mathbf{I} - \tilde{\mathbf{\Pi}}_{\mathbf{Y}_T}\mathbf{L}^\mathsf{T}\left(\mathbf{L}\tilde{\mathbf{\Pi}}_{\mathbf{Y}_T}\mathbf{L}^\mathsf{T} - \begin{bmatrix} N\sigma_{\mathbf{Y}}^2\mathbf{Q} & 0 \\ 0 & N\sigma_{\mathbf{U}}^2\mathbf{R}\end{bmatrix}\right)_\varepsilon^\dagger \mathbf{L}\right)\mathbf{Y}_T^\mathsf{T}(\mathbf{Y}_T\mathbf{Y}_T^\mathsf{T} - N\sigma_{\mathbf{Y}_T}^2\mathbf{I})^\dagger\mathbf{y}_\mathrm{f} \\
&= \mathbf{U}_{0:T-1}\mathbf{Y}_T^\mathsf{T}(\mathbf{Y}_T\mathbf{Y}_T^\mathsf{T} - N\sigma_{\mathbf{Y}_T}^2\mathbf{I})^\dagger\mathbf{y}_\mathrm{f} - \mathbf{U}_{0:T-1}\tilde{\mathbf{\Pi}}_{\mathbf{Y}_T}\mathbf{L}^\mathsf{T}\cdot \\
&\quad\cdot\left(\mathbf{L}\tilde{\mathbf{\Pi}}_{\mathbf{Y}_T}\mathbf{L}^\mathsf{T} - \begin{bmatrix} N\sigma_{\mathbf{Y}}^2\mathbf{Q} & 0 \\ 0 & N\sigma_{\mathbf{U}}^2\mathbf{R}\end{bmatrix}\right)_\varepsilon^\dagger \mathbf{L}\mathbf{Y}_T^\mathsf{T}(\mathbf{Y}_T\mathbf{Y}_T^\mathsf{T} - N\sigma_{\mathbf{Y}_T}^2\mathbf{I})^\dagger\mathbf{y}_\mathrm{f} \\
&= \mathbf{P}_2(\mathbf{P}_1 - \sigma_{\mathbf{Y}_T}^2\mathbf{I})^\dagger\mathbf{y}_\mathrm{f} - \big(\mathbf{P}_3 - \mathbf{P}_2(\mathbf{P}_1 - \sigma_{\mathbf{Y}_T}^2\mathbf{I})^\dagger\mathbf{P}_4\big)\cdot \\
&\quad\cdot\left(-\mathbf{P}_4^\mathsf{T}(\mathbf{P}_1 - \sigma_{\mathbf{Y}_T}^2\mathbf{I})^\dagger\mathbf{P}_4 + \mathbf{P}_5 - \begin{bmatrix} \sigma_{\mathbf{Y}}^2\mathbf{Q} & 0 \\ 0 & \sigma_{\mathbf{U}}^2\mathbf{R}\end{bmatrix}\right)_\varepsilon^\dagger \mathbf{P}_4^\mathsf{T}(\mathbf{P}_1 - \sigma_{\mathbf{Y}_T}^2\mathbf{I})^\dagger\mathbf{y}_\mathrm{f}
\end{aligned} \tag{30}$$

where $\mathbf{P}_1 := \frac{1}{N}\mathbf{Y}_T\mathbf{Y}_T^\mathsf{T}$, $\mathbf{P}_2 := \frac{1}{N}\mathbf{U}_{0:T-1}\mathbf{Y}_T^\mathsf{T}$, $\mathbf{P}_3 := \frac{1}{N}\mathbf{U}_{0:T-1}\mathbf{L}^\mathsf{T}$, $\mathbf{P}_4 := \frac{1}{N}\mathbf{Y}_T\mathbf{L}^\mathsf{T}$, and $\mathbf{P}_5 := \frac{1}{N}\mathbf{L}\mathbf{L}^\mathsf{T}$. By the Strong Law of Large Numbers [13] and the assumption on the noise, as

$N \to \infty$, it follows that

$$
\begin{aligned}
\mathbf{P}_1 &= \frac{1}{N}\mathbf{Y}_T\mathbf{Y}_T^\top \xrightarrow{\text{a.s.}} \frac{1}{N}\bar{\mathbf{Y}}_T\bar{\mathbf{Y}}_T^\top + \sigma_{\mathbf{Y}_T}^2\mathbf{I} =: \bar{\mathbf{P}}_1,\\
\mathbf{P}_2 &= \frac{1}{N}\mathbf{U}_{0:T-1}\mathbf{Y}_T^\top \xrightarrow{\text{a.s.}} \frac{1}{N}\bar{\mathbf{U}}_{0:T-1}\bar{\mathbf{Y}}_T^\top =: \bar{\mathbf{P}}_2,\\
\mathbf{P}_3 &= \frac{1}{N}\mathbf{U}_{0:T-1}\mathbf{L}^\top \xrightarrow{\text{a.s.}} \frac{1}{N}\bar{\mathbf{U}}_{0:T-1}\begin{bmatrix}\bar{\mathbf{Y}}_{1:T-1}^\top\mathbf{Q}^{1/2} & \bar{\mathbf{U}}_{0:T-1}^\top\mathbf{R}^{1/2}\end{bmatrix} =: \bar{\mathbf{P}}_3,\\
\mathbf{P}_4 &= \frac{1}{N}\mathbf{Y}_T\mathbf{L}^\top \xrightarrow{\text{a.s.}} \frac{1}{N}\bar{\mathbf{Y}}_T\begin{bmatrix}\bar{\mathbf{Y}}_{1:T-1}^\top\mathbf{Q}^{1/2} & \bar{\mathbf{U}}_{0:T-1}^\top\mathbf{R}^{1/2}\end{bmatrix} =: \bar{\mathbf{P}}_4,\\
\mathbf{P}_5 &= \frac{1}{N}\mathbf{L}\mathbf{L}^\top \xrightarrow{\text{a.s.}} \begin{bmatrix}\frac{1}{N}\mathbf{Q}^{1/2}\bar{\mathbf{Y}}_{1:T-1}\bar{\mathbf{Y}}_{1:T-1}^\top\mathbf{Q}^{1/2}+\sigma_{\mathbf{Y}}^2\mathbf{Q} & \frac{1}{N}\mathbf{Q}^{1/2}\bar{\mathbf{Y}}_{1:T-1}\bar{\mathbf{U}}_{0:T-1}^\top\mathbf{R}^{1/2}\\ \frac{1}{N}\mathbf{R}^{1/2}\bar{\mathbf{U}}_{0:T-1}\bar{\mathbf{Y}}_{1:T-1}^\top\mathbf{Q}^{1/2} & \frac{1}{N}\mathbf{R}^{1/2}\bar{\mathbf{U}}_{0:T-1}\bar{\mathbf{U}}_{0:T-1}^\top\mathbf{R}^{1/2}+\sigma_{\mathbf{U}}^2\mathbf{R}\end{bmatrix} =: \bar{\mathbf{P}}_5.
\end{aligned}
\tag{31}
$$

Notice that $\hat{\mathbf{u}}_{0:T-1}^{(c)}$ is a continuous function of $\mathbf{P}_i$ around $\mathbf{P}_i = \bar{\mathbf{P}}_i$ for $i = 1, \ldots, 5$ and $\varepsilon > 0$ sufficiently small. In light of this fact, (29) follows by using (31) and the Continuous Mapping Theorem [13, Theorem 2.3]. □

Following the same argument as above, it is possible to establish asymptotically correct data-driven expressions of minimum-energy controls ($\mathbf{Q} = \mathbf{0}$, $\mathbf{R} = \mathbf{I}$). Specifically, the corrected version of (10) reads as

$$\hat{\mathbf{u}}_{0:T-1}^{(c)} = (\mathbf{I} - \mathbf{U}_{0:T-1}\tilde{\mathbf{\Pi}}_{\mathbf{Y}_T}(\mathbf{U}_{0:T-1}\tilde{\mathbf{\Pi}}_{\mathbf{Y}_T}\mathbf{U}_{0:T-1}^\top - N\sigma_{\mathbf{U}}^2\mathbf{I})_{\varepsilon}^{\dagger})\mathbf{U}_{0:T-1}(\mathbf{Y}_T\mathbf{Y}_T^\top - N\sigma_{\mathbf{Y}_T}^2\mathbf{I})^{\dagger}\mathbf{y}_{\mathrm{f}}, \tag{32}$$

whereas the corrected version of the compact data-driven control in (15) is

$$\hat{\mathbf{u}}_{0:T-1}^{(c)} = (\mathbf{Y}_T\mathbf{U}_{0:T-1}^\top(\mathbf{U}_{0:T-1}\mathbf{U}_{0:T-1}^\top - N\sigma_{\mathbf{U}}^2\mathbf{I})^{\dagger})^{\dagger}\mathbf{y}_{\mathrm{f}}. \tag{33}$$

Finally, the corrected approximate minimum-energy control in (16) reads as

$$\tilde{\mathbf{u}}_{0:T-1}^{(c)} = \mathbf{U}_{0:T-1}(\mathbf{Y}_T\mathbf{Y}_T^\top - N\sigma_{\mathbf{Y}_T}^2\mathbf{I})^{\dagger}\mathbf{y}_{\mathrm{f}}. \tag{34}$$

It is worth noting that, while (32) requires correction terms for both input and output noises, (33) and (34) include correction terms only for one source of noise (input noise in (33) and output noise in (34)). In particular, if the noise corrupts output data only, (33) coincides with the original data-driven control in (15).

SUPPLEMENTARY REFERENCES

[1] Sarachik, P. E. & Kreindler, E. Controllability and observability of linear discrete-time systems. *International Journal of Control* **1**, 419–432 (1965).

[2] Kailath, T. *Linear Systems* (Prentice-Hall, 1980).

[3] Gao, J., Liu, Y.-Y., D'Souza, R. M. & Barabási, A. L. Target control of complex networks. *Nature communications* **5**, 5415 (2014).

[4] Klickstein, I., Shirin, A. & Sorrentino, F. Energy scaling of targeted optimal control of complex networks. *Nature communications* **8**, 15145 (2017).

[5] Reinschke, K. J. *Multivariable Control: A Graph-Theoretic Approach* (Springer, 1988).

[6] Cowan, N. J., Chastain, E. J., Vilhena, D. A., Freudenberg, J. S. & Bergstrom, C. T. Nodal dynamics, not degree distributions, determine the structural controllability of complex networks. *PLoS ONE* **7**, e38398 (2012).

[7] Menara, T., Bassett, D. S. & Pasqualetti, F. Structural controllability of symmetric networks. *IEEE Transactions on Automatic Control* **64**, 3740–3747 (2019).

[8] Ben-Israel, A. & Greville, T. N. E. *Generalized inverses: theory and applications*, vol. 15 of *CMS Books in Mathematics* (Springer-Verlag New York, 2003), 2nd edn.

[9] Vershynin, R. *Introduction to the non-asymptotic analysis of random matrices*, 210–268 (Cambridge University Press, 2012).

[10] Searle, S. R. *Matrix algebra useful for statistics* (John Wiley & Sons, 1982).

[11] Pasqualetti, F., Zampieri, S. & Bullo, F. Controllability metrics, limitations and algorithms for complex networks. *IEEE Transactions on Control of Network Systems* **1**, 40–52 (2014).

[12] Dean, S., Mania, H., Matni, N., Recht, B. & Tu, S. On the sample complexity of the linear quadratic regulator. *Foundations of Computational Mathematics* 1–47 (2019).

[13] Van der Vaart, A. W. *Asymptotic statistics*, vol. 3 of *Cambridge Series in Statistical and Probabilistic Mathematics* (Cambridge University Press, 2000).

SUPPLEMENTARY FIGURES

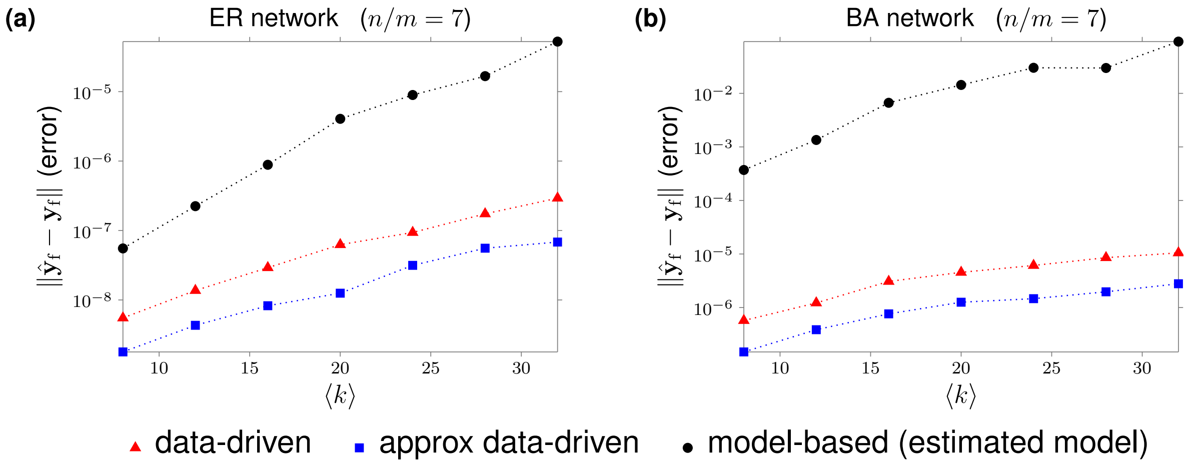

SUPPLEMENTARY FIGURE 1. **Effect of average degree on numerical accuracy.** We compare the error in the final state ($\mathbf{C} = \mathbf{I}$) generated by the data-driven minimum-energy control inputs (Equations (5) and (6) of the main text) and model-based expression as a function of the average degree $\langle k \rangle$ for Erdös–Rényi (panel **(a)**) and Barabási–Albert networks (panel **(b)**). We choose $n = 700$ and $m = 100$. The remaining parameters are as in Fig. 3**(c)**-**(d)** of the main text. All curves represent the average over 100 realizations of networks, data, control nodes, and final states.

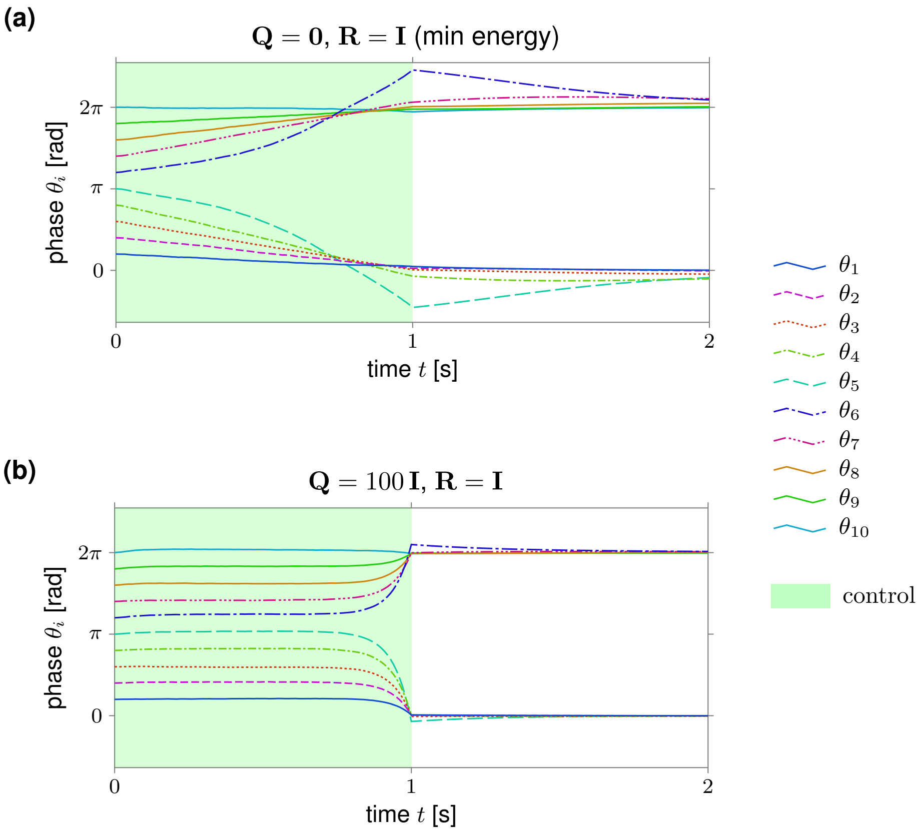

SUPPLEMENTARY FIGURE 2. **Role of Q in the control of synchronized patterns in Kuramoto networks.** We consider a ring network of $n = 10$ Kuramoto oscillators where all nodes are controlled ($m = 10$) as in the Kuramoto example of the main text. We apply the data-driven control in Eq. (4) of the main text to steer the phases from the synchronous state to the splay state $\{\bar{\theta}_{1,i}(t)\}$. In panel **(a)** we choose $\mathbf{Q} = \mathbf{0}$, $\mathbf{R} = \mathbf{I}$. In panel **(b)** we choose $\mathbf{Q} = 100\,\mathbf{I}$, $\mathbf{R} = \mathbf{I}$. The green region denotes the application of the control. We choose $T = 100$ samples of the discretize dynamics (corresponding to a control horizon of 1s), $N = 2000$ data obtained by perturbing the initial equilibrium with i.i.d. Gaussian inputs with zero mean and standard deviation 0.1. The green region denotes the application of the control. Note that the controlled trajectories of panel **(b)** are much closer to the starting equilibrium than those of panel **(a)** resulting in a much smaller error in the final state and a faster convergence to the desired splay state.
