# Supplementary material for: Data-driven control of complex networks
Source: Nat Commun. 2021 Mar 3;12:1429. doi: 10.1038/s41467-021-21554-0 (PMC7930026; doi:10.1038/s41467-021-21554-0)
Supplement: Supplementary file 2 — Peer Review File [file 41467_2021_21554_MOESM2_ESM.pdf]

Reviewer #1 (Remarks to the Author):

I have reviewed the manuscript entitled "Data-Driven Control of Complex Networks." This paper studies a very interesting and important question: how to control a complex network based on the control input and state data? Overall, the article is well written. Yet, I do have several concerns and comments on the current form of the manuscript. I am quite open to looking at a revised version if the author can address the issues discussed below:

1. In the abstract, the authors mentioned that the current issue of realistic control is "when the network topology is large and, possibly, time-varying." (1) Many existing papers about network control studied networks with several thousand or even millions of nodes. In this paper, the authors studied networks with a hundred nodes. (2) In the model, the authors only study the time-invariant dynamics. It seems that large size and time-varying is not related to this paper. A small suggestion: it is strange to say, "topology is large."
2. The authors studied the data-driven control, but the authors use a linear system. It is very hard to find data about real-world systems that follow linear dynamics. Of course, I saw that the authors apply this approach to brain dynamics. I am not convinced that the linear system can model the brain dynamics.
3. Section A of the Results is about point-to-point control. But I did not find its definition.
4. In Eq. (2) the authors give the optimization problem. Why the authors have to solve this problem? It seems that the authors would like to minimize energy (u) and y. The form is similar to the linear-quadratic control but different. I am not familiar with the notations. It will be great if the authors define what y_{1:T-1} is. How to choose Q and R in real systems?
5. In Eq. (4), the authors solve the optimization and give the input signal \hat{u}. What are \hat{u} and u? Which one will satisfy Eq. (1), or both?
6. It seems that Eq. (4) is independent with network A, controller B, and outputs C.

Why do these important parameters not affect the control signals?

More importantly, in this research, the authors did not discuss the controllability of the networks. In other words, in some conditions, the networks are uncontrollable. Then, how can one design the signals? We also know that the network topology (heterogeneity and density), selections of driver nodes, and output nodes are all important to networks' controllability. It will be great if the authors show how do these factors determine the results.

7. The authors numerically checked the minimum data. How does the minimum data depend on the parameters we mentioned above, and where (the direction) you want to control?
8. In the "numerical and computational benefits of data-driven controls," the authors use ER networks with n nodes and m control nodes, where m is about n/10 or n/100. We know that the minimal number of driver nodes controlling an ER network with linear dynamics follows exp{-<k>/2}. In other words, if the network is too sparse, n/10 nodes may not be able to control the network. Furthermore, the controllability also depends on how you select the driver nodes. I suggest the author will discuss these issues more.

Reviewer #2 (Remarks to the Author):

As we know, to solve the empirical problem of network science, identifying the network structure and dynamics details is a premise work. However, errors and limitations are unavoidable in this process, and obviously can influence the subsequent studies. Focus on the network controllability, this manuscript tries to overcome this problem by learning control strategy directly from data. Through simulations, the authors showed that their data-driven method are more accurate and computationally more efficient than model-based ones. This indicates that many real control problems might be solved directly and simply from the experimental data. I think that this is an important step for the research of network controllability, and therefore I recommends publication.

Besides, I have two questions. First, we know that real networks often have scale-free or small-world effects. Does this data-driven method work well for such networks? Second, the authors give some figures to show the effects of the system size on the performance of their method, such as Fig.1 (c), Fig.2.(c), and Fig.3. In statistical physics, such a size-dependent property is often studied as finite-size scaling, which can be used to reveal or predict the property of the infinite system. My question is that can some scaling behaviors be extracted from these figures. We know that the real network often has large size. Can these results be used to predict the performance of a much large system?

Reviewer #3 (Remarks to the Author):

This paper solves the discrete time, finite-time, open loop, linear-quadratic optimal control problem using a set of input-output data rather than the system matrices (A,B,C) directly.
While there is much work on learning optimal controls for the discrete time LQR problem (which is solved in the infinite-time and feedback control framework) I was unable to find any previous work that addressed the model-free, finite-time and open loop case, so the main result of this paper appears to be novel.

Determining controls, in particular optimal controls, that are based on data rather than detailed information about the underlying, possibly extremely complicated, network system, is a topic that should be of interest to a general audience.

Nonetheless, I have the following concerns that should be addressed.

I recommend, for the general audience, to be specific about the necessary and sufficient conditions for a discrete time linear system, (A,B,C) and T, to be output controllable, rather than just reference the book by Kailath, as this assumption is integral in the application of the remaining results. The requirements on T is important and is an important difference from the continuous time case where controllability does not depend on the final time T.

Fig. 1C, edge weight uncertainty is zero, but there is still an error present in the final state, why is this? shouldn't it be exact?

What is the difference between u^* and \hat{u} in Eq. (2) and Eq. (4), isn't Eq. (4) suppose to be the solution of Eq. (2)? Reading on I see that \hat{u} is the 'data-driven' input while u^* is the true optimal solution. This should be stated earlier.

When deriving the Minimum-Energy Control Input, in section IV in the SI, it is stated that for Q = O and R = I one can replace L = I. But from section I in the SI I believe it should be that L = U_{0:T-1}^T U_{0:T-1} where the input data could be constructed such that L = I but for arbitrary inputs I do not believe this is the case.

In Eq. (6) in the main text, an approximate data-driven input for the Minimum Energy problem is presented, but it reappears as an exact equality in Eq. (14) in the SI. Could the authors please explain this discrepancy?

In the application to the New England power grid, I have a few questions:
(1) Is the data collected from the linearized model or the original continuous nonlinear model?
(2) Is the simulation with the control input done with the linearized discrete model? Or is the discrete time control input applied the linearized model?
I ask these questions because linearizations are only valid close to the point about which the linearization is applied and I do not think this subtlety is addressed in the discussion about this application.

Comments by Referee # 1

[R1: 1] "I have reviewed the manuscript entitled "Data-Driven Control of Complex Networks." This paper studies a very interesting and important question: how to control a complex network based on the control input and state data? Overall, the article is well written. Yet, I do have several concerns and comments on the current form of the manuscript. I am quite open to looking at a revised version if the author can address the issues discussed below:"

We thank the reviewer for carefully reading our paper, for the their appreciation of our work, and for their very valuable feedback. We have made a major effort to improve our paper following the reviewer's constructive comments and suggestions, as detailed in the point-by-point responses below.

[R1: 2] "1. In the abstract, the authors mentioned that the current issue of realistic control is "when the network topology is large and, possibly, time-varying." (1) Many existing papers about network control studied networks with several thousand or even millions of nodes. In this paper, the authors studied networks with a hundred nodes."

The reviewer raises an important point, which pertains to the curse of dimensionality in studying minimum-energy (and, more generally, quadratically-optimal) control problems.

First, in the revised manuscript we have replaced our numerical studies of Figures 2(b) and 3(a) with new ones using a larger network dimension (namely, $n = 1000$). However, we point out that in these problems the "critical" parameter is not solely the dimension of the network n, but rather the ratio between n and the number of control nodes m (or, if we are interested in controlling the states of a subset of output nodes of the network, the ratio between the number of output and control nodes p/m). In other words, the numerical performance of optimal controls is approximately the same for networks generated via the same model and featuring the same ratio n/m. As n/m grows (for instance, when the number of control nodes is fixed, independent of n, and the dimension of the network increases), the energy to control a network typically grows exponentially resulting in a drop in the numerical performance of optimal controls, as demonstrated in a series of papers, e.g., see Refs. [1–4]. Equivalently, the smallest eigenvalues of the controllability Gramian decay exponentially with n/m, leading to numerical instabilities and inaccuracies when attempting to compute minimum-energy controls for large networks with few control nodes (in some cases, the computation of the controllability Gramian becomes problematic for networks with only tens of nodes). Some sentences in the abstract and in the introduction of the first version of the paper (including the one mentioned by the reviewer) were inaccurate; we have now clarified these sentences in the revised version of the manuscript. Further, in Section II-B.3, we have clarified that the key parameter regulating the numerical performance of optimal controls is the ratio n/m (see footnote 3).

Second, for the same reasons as above, in our paper we decided to use example networks with a not-too-large ratio n/m or p/m, so as to be able to accurately compare data-driven and model-based minimum-energy and quadratically-optimal controls, and to validate our expressions. For larger ratios, since the exponential growth of the energy of optimal controls is independent of the method used to compute such inputs, data-driven expressions also suffer from numerical instabilities. Yet, we observed that the numerical performance of the data-driven formulas deteriorates less rapidly compared to the model-based one when the network is reconstructed from data, as we now show by means of a new numerical study. In Fig. R1(a), we compare the error in the final state generated by data-driven and model-based minimum-energy controls for Erdös–Rényi networks with two different average degrees as a function of the ratio n/m. The model-based control is computed using a network estimated from data via the subspace-based identification scheme described in Methods, whereas the data-driven control is computed using the exact and approximate formulas of the main text (Equations (5) and (6), respectively). We choose the target state x_{f} and control nodes randomly (with the only constraint that the resulting network is controllable). Although both the

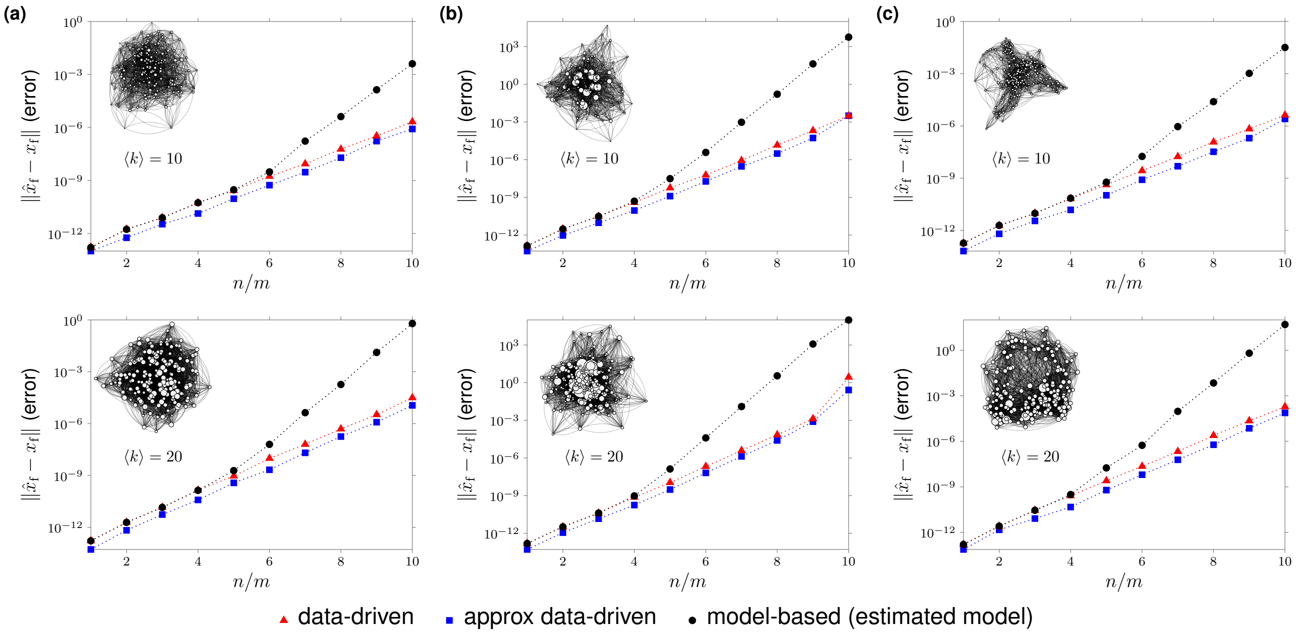

Figure R1: **Numerical accuracy of data-driven and model-based controls.** We compare the error in the final state generated by the data-driven control inputs (Equations (5) and (6) of the main text) and the model-based minimum-energy input for a fixed number of control nodes $m = 100$ and increasing dimension $n \in [100, 1000]$. The model-based control has been computed by first estimating matrices A and B from data according to the subspace-based method described in Methods, and then applying the model-based control expression. In Panel **(a)**, we consider an Erdös–Rényi network with average degree $\langle k \rangle = 10$ (top) and $\langle k \rangle = 20$ (bottom). In Panel **(b)**, we consider a Barabási–Albert network [5] with initial number of nodes $m_0 = 20$ and average degree $\langle k \rangle = 10$ (top) and $\langle k \rangle = 20$ (bottom). In Panel **(c)**, we consider a Watts–Strogatz network [6] with probability of rewiring $p_{\text{rew}} = 0.2$ and average degree $\langle k \rangle = 10$ (top) and $\langle k \rangle = 20$ (bottom). In all plots we use a control horizon $T = 15$ and a number of data $N = mT + 200$ generated using i.i.d. normal inputs. To limit the influence of the eigenvalues of A in the computation of optimal controls across different network models, we normalize the adjacency matrix A by its norm $\|A\|$. The m control nodes and the final state x_f are randomly generated, with the only constraint that the resulting system is controllable (see Methods for further details). The curves represent the average over 100 realizations of networks, data, control nodes, and final states.

data-driven and the model-based controls yield a poor numerical accuracy for increasing values of n/m[1], the model-based input exhibits a faster growth of the error when compared to the data-driven ones. We found similar tradeoffs for other random network models. In particular, in Fig. R1(b) and Fig. R1(c), we repeat the same numerical experiment as above for Barabási–Albert (scale-free) and Watts–Strogatz (small-world) networks with two different values of the average degree. In addition, as shown more clearly in Fig. R2, we empirically observed that the gain in numerical accuracy offered by data-driven control inputs is more significant for dense networks (independently of their degree heterogeneity).[2] In the revised version of the paper, we have replaced the former Fig. 3(b) with Fig. R1, which we believe is more complete and insightful, and we have modified accordingly some parts of Section II-B.3. Further, we have included Fig. R2 in the Supplement.

[1] We numerically found that both expressions become unreliable for ratios n/m approximately larger than 10.

[2] This may be due to the fact that the magnitude of the numerical errors in the network reconstruction process (which are then propagated in the computation of the control) are likely to be proportional to the number of to-be-identified edges.

Finally, our numerical study also suggests that the approximate data-driven control formula, although suboptimal, typically yields the best performance. This fact indicates another potential advantage in using a data-driven approach: the latter may generate suboptimal, yet accurate, solutions which have no known model-based counterpart. We have mentioned this fact in the revised version of the paper (lines 205-208).

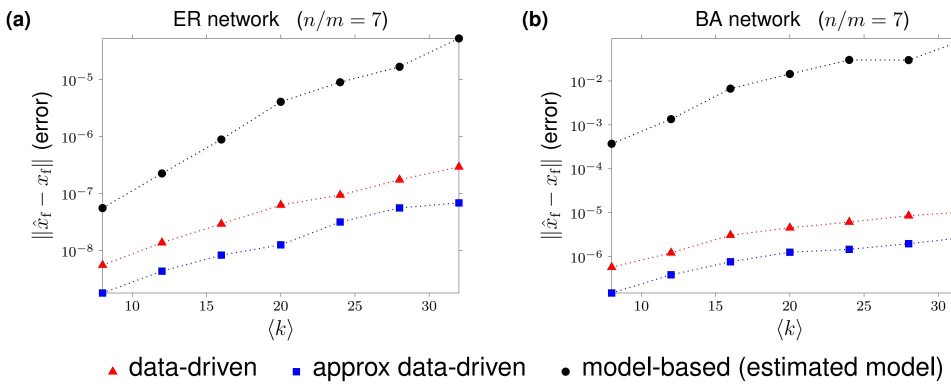

Figure R2: **Effect of average degree on numerical accuracy.** We compare the error in the final state generated by the data-driven minimum-energy control inputs (Equations (5) and (6) of the main text) and model-based expression as a function of the average degree $\langle k \rangle$ for Erdös–Rényi (panel **(a)**) and Barabási–Albert networks (panel **(b)**). We choose $n = 700$ and $m = 100$. The remaining parameters are as in Fig. R1. All curves represent the average over 100 realizations of networks, data, control nodes, and final states.

[R1: 3] "(2) In the model, the authors only study the time-invariant dynamics. It seems that large size and time-varying is not related to this paper."

The reviewer is correct that in this paper we do not address issues arising with time-varying dynamics. The point that we wanted to make is that data-driven approaches are typically more flexible than model-based ones, since they can tolerate time-varying dynamics provided that experimental data is continuously generated and that the changes are sufficiently slow (for instance, due to the aging of some components in technological networks). However, we agree with the reviewer that mentioning "time-varying" in the abstract and introduction may be inaccurate. Thus, we have decided to remove such reference.

[R1: 4] "A small suggestion: it is strange to say, "topology is large.""

Thanks. We have fixed this. We have now replaced "topology" with "dimension".

[R1: 5] "2. The authors studied the data-driven control, but the authors use a linear system. It is very hard to find data about real-world systems that follow linear dynamics."

The reviewer is correct that our theory of data-driven controls is developed for networks with linear dynamics. However, we stress that:

(a) there are many real-world systems whose dynamics are inherently linear (e.g., electric and mechanical networks [7, Ch. 6]; see also [4, 8] for explicit examples focusing on the controllability of such networks) or can be effectively modeled by linear differential equations (e.g., standard models of opinion dynamics [9, 10]). More generally, the dynamics of most technological systems are *designed*, whenever

possible, to be as linear as possible (at least, within the regime of interest) since this simplifies their operation and control (as evidenced by the extensive literature on linear control systems). Thus, we believe that our theory of data-driven control for linear systems is not only necessary as a first step to develop a comprehensive theory of data-driven control for nonlinear systems, but also relevant for several practical applications.

(b) our techniques are often applicable even when data is generated by nonlinear systems, as we demonstrate in the paper for our power network and brain examples (where data is generated using the underlying nonlinear dynamics). This is not surprising, since linear control methods are often used also for systems with nonlinear dynamics (in certain regimes). Thus, while we focus on linear dynamics to derive our theoretical results and guarantees, our methods remain of theoretical and practical interest.

In the revised manuscript (Section II-C.1), we have added an additional example to validate our methods on a network with nonlinear dynamics. Specifically, we apply our data-driven formulas to induce desired patterns of synchronization in a network of phase oscillators. The latter problem has several applications in neuroscience [11] and power network analysis [12] (see also Refs. [13, 14] and the references therein).

We consider a ring network of n Kuramoto oscillators. The dynamics of the phases of the oscillators are

$$\dot{\theta}_i(t) = \omega_i + \sin(\theta_{i-1}(t) - \theta_i(t)) + \sin(\theta_{i+1}(t) - \theta_i(t)), \quad i = 1, \dots, n, \tag{1}$$

where ω_i is the natural frequency of the i-th oscillator and the index i is periodic mod n. We consider the case where $\omega_i = \omega$ for all i. In this case, the network always has a stable synchronous state given by $\bar{\theta}_i(t) = \omega t$ for all i. However, as n grows other stable equilibra arise, namely [15]:

$$\bar{\theta}_{q,i}(t) = \omega t + \frac{2\pi q i}{n} + c, \quad i = 1, \dots, n, \tag{2}$$

where c is an arbitrary constant and q is the winding number which takes integer values. These equilibria correspond to phases linearly spaced on the unit circle and are commonly referred to as splay states. To simulate the dynamics in Eq. (1) and generate the data, we discretize the Kuramoto dynamics via the forward Euler method with discretization step 0.01. In Fig. R3, we consider a network of $n = 10$ oscillators with $\omega = 0$. In the top plots, we assume that we have access to all nodes of the network ($m = 10$) and apply an external control input to steer the nework: (i) from the splay state $\{\bar{\theta}_{1,i}(t)\}$ to the synchronous state $\{\bar{\theta}_i(t)\}$ (Fig. R3(b)), and (ii) from the splay state $\{\bar{\theta}_{2,i}(t)\}$ to the splay state $\{\bar{\theta}_{1,i}(t)\}$ (Fig. R3(c)). The control input has been computed using the data-driven expression in Eq. (4) of the main text with parameters $T = 50$ samples (corresponding to a control horizon of 0.5s), $Q = 5$, $R = 1$. Data have been generated through $N = 1000$ control experiments obtained by perturbing the initial equilibrium with i.i.d. Gaussian external inputs with zero mean and standard deviation 0.1. In the right plots of Fig. R3, we repeat the same experiments using $m = 3$ control nodes (red nodes in Fig. R3(d)). In both scenarios, the control input does not exactly drive the network to the desired target state (because of the nonlinearity of the dynamics) but to a state close to it. Nevertheless, the final state falls within the basin of attraction of the desired target equilibrium so that the network reaches asymptotically the desired pattern of synchronization.

(c) even when our data-driven control expressions are not applicable, they could still be useful to shed light on the controllability properties of nonlinear networks. In particular, our formulas can be employed to give (quantitative, data-based) insights on the easiest/most difficult control directions (and, more generally, on the control energy “landscape” of the network) or to inform the selection of control nodes (for instance, by computing the norm of the minimum-energy control input for different choices of the set of input nodes). We remark that tools from linear control theory have been fruitfully

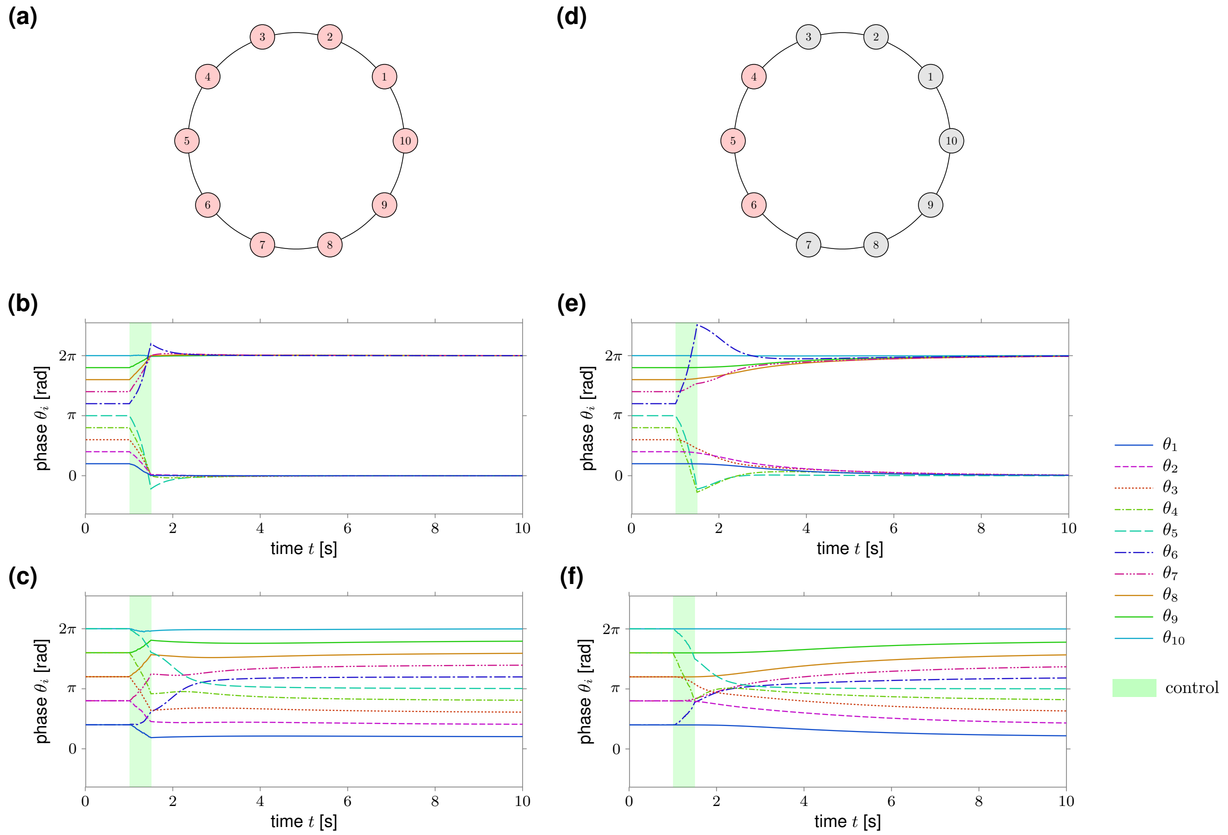

Figure R3: **Data-driven control of synchronized patterns in a ring of Kuramoto oscillators.** We consider a ring network of $n = 10$ Kuramoto oscillators for two different configurations of control nodes (red nodes), namely $m = 10$ (panel **(a)**) and $m = 3$ (panel **(d)**). For both configurations, we apply the data-driven control (4) to steer the phases from the splay state $\{\bar{\theta}_{1,i}(t)\}$ to the synchronous state (panels **(b)** and **(e)**) and from the splay state $\{\bar{\theta}_{2,i}(t)\}$ to $\{\bar{\theta}_{1,i}(t)\}$ (panels **(c)** and **(f)**). The green region denotes the application of the control. We choose as parameters $T = 50$ samples of the discretize dynamics (corresponding to a control horizon of 0.5s), $Q = 5$, $R = 1$, and $N = 1000$ data obtained by perturbing the initial equilibrium with i.i.d. Gaussian inputs with zero mean and a standard deviation 0.1.

applied to shed light on these and other control-related problems in several real nonlinear networks (most notably biological networks, e.g., see Refs. [16, 17]). Thus, our theory offers an alternative, data-driven perspective for the solution to these problems. We have included these observations in the Introduction and Discussion of the revised version of the paper.

[R1: 6] "Of course, I saw that the authors apply this approach to brain dynamics. I am not convinced that the linear system can model the brain dynamics."

The reviewer is correct that brain dynamics are likely nonlinear, and that a linear model can only provide an approximation in certain operating conditions. However, this discrepancy does not prevent the use of linear controls, which, as shown in our study (and in fact in a recent line of literature, e.g., see Refs. [16, 18, 19]), can still provide a reasonable approximation of the actual trajectories. In fact, linear control techniques can often offer satisfactory performance even when applied to nonlinear systems, at least in certain operating

regimes (see, for instance, *gain scheduling* [20]). We have added this important caveat in the revised manuscript (see footnote 6). The validity of our linear data-driven techniques for nonlinear systems is also confirmed in our second example, where the underlying system exhibits nonlinear power systems dynamics, and in the additional example that we have included in the revised manuscript.

[R1: 7] "3. Section A of the Results is about point-to-point control. But I did not find its definition."

Thank you. We have added the definition of "point-to-point control" in Section II-A of the revised manuscript (lines 80-82). Loosely speaking, point-to-point control refers to the problem of finding a control input that steers the system from an initial state to a final state in finite time.

[R1: 8] "4. In Eq. (2) the authors give the optimization problem. Why the authors have to solve this problem? It seems that the authors would like to minimize energy (u) and y. The form is similar to the linear-quadratic control but different."

The reviewer is correct that Eq. (2) is a general version of the linear-quadratic control problem. In particular, if $C = I$ in Eq. (1), then the problem posed in Eq. (2) reduces to the classic (finite time) linear-quadratic (point-to-point) control problem. The current formulation extends the classic linear-quadratic control framework by including the possibility of minimizing a linear function of the state (as opposed to the whole state) in addition to the control input. Notice that all the results in the paper are valid also for the case of $C = I$, and that the current linear-quadratic formulation includes the minimum-energy control problem as a special case, which is obtained by choosing $Q = 0$. Thus, our current formulation generalizes several studies on the design of control inputs for network systems. In the revised manuscript, we have clarified the relations between our formulation and the classic linear-quadratic control problem (lines 94-96).

[R1: 9] "I am not familiar with the notations. It will be great if the authors define what $y_{1:T-1}$ is."

Thank you. The vector $y_{1:T-1}$ denotes the stacked vector of the measurements from time $t = 1$ up to time $t = T - 1$. We have clarified the notation in the revised manuscript (lines 93-94).

[R1: 10] "How to choose Q and R in real systems?"

The matrices Q and R allow the designer to tune the control objective and are selected based on the task at hand. As done in linear-quadratic control [21, Ch. 9], the matrix R imposes a cost on the energy of the input: the larger the R, the larger the cost of using large inputs. Thus, increasing R leads to optimal control inputs that achieve the desired final state with increasingly smaller inputs. When R approaches infinity (equivalently, when $Q = 0$), the optimization problem returns the minimum-energy input to reach the desired final state. Similarly, the matrix Q weighs the norm of the output (state). Increasing Q forces the optimization problem to generate inputs that limit the norm of the output (state), at the expenses of using a larger control input. Increasing Q to infinity (equivalently, decreasing R to zero) leads to the *cheap control problem*, which minimizes the output deviation without consideration of the input energy. We have clarified this point in the revised manuscript (lines 96-102 and footnote 2). There are several "empirical" ways to tune the matrices Q and R. A simple and reasonable choice is given by *Bryson's rule* [21, Ch. 9.5]: Select Q and R diagonal with

$$Q_{ii} = \frac{1}{\text{maximum acceptable value of } y_i^2}, \quad i = 1, \ldots, p, \tag{3}$$

$$R_{ii} = \frac{1}{\text{maximum acceptable value of } u_i^2}, \quad i = 1, \ldots, m. \tag{4}$$

This rule scales the variables that appear in the cost of problem posed in Eq. (2) so that the maximum acceptable value for each variable is 1. This is especially important when the units used for the different components of the input and output make the values for these variables numerically very different from each other.

Finally, we point out that the choice of Q is particularly important when our data-driven expressions are applied to nonlinear dynamical networks. In particular, Q can be tuned to favor trajectories with a small deviation from an equilibrium, thus improving the applicability and effectiveness of our methods, since a nonlinear system approximately behaves as a linear one in a sufficiently small neighborhood of an equilibrium.[3] In Fig. R4 we illustrate this fact for the Kuramoto example described in our reply to comment [R1: 6]. Specifically, we apply the data-driven control input to steer the network from the synchronous state to the splay state $\{\bar{\theta}_{1,i}(t)\}$. In Fig. R4(a) we choose $Q = 0$, $R = I$ (minimum-energy control), whereas in Fig. R4(b) $Q = 100I$, $R = I$. The controlled trajectories of Fig. R4(b) are much closer to the starting equilibrium than those of Fig. R4(b), resulting in a much smaller error in the final state and a faster convergence to the desired splay state. We have included this observation in the revised manuscript (lines 285-289) and added Fig. R4 to the Supplement.

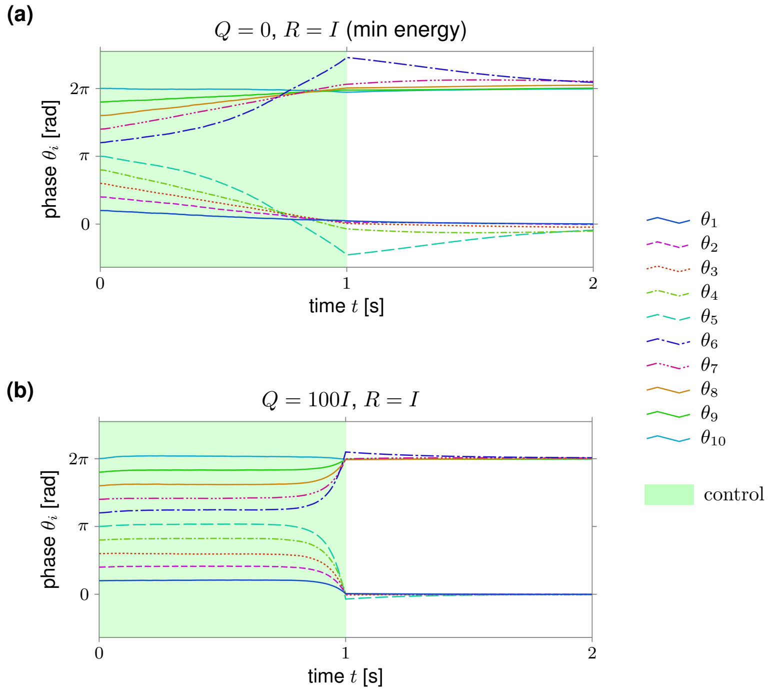

Figure R4: **Role of Q in the control of synchronized patterns in Kuramoto networks.** We consider a ring network of $n = 10$ Kuramoto oscillators where all nodes are controlled ($m = 10$). We apply the data-driven control (4) to steer the phases from the synchronous state to the splay state $\{\bar{\theta}_{1,i}(t)\}$. In panel **(a)** we choose $Q = 0$, $R = I$. In panel **(b)** we choose $Q = 100I$, $R = I$. The green region denotes the application of the control. We choose $T = 100$ samples of the discretize dynamics (corresponding to a control horizon of 1s), $N = 1000$ data obtained by perturbing the initial equilibrium with i.i.d. Gaussian inputs with zero mean and standard deviation 0.1.

[3] When the starting equilibrium is different from the origin, the following shifted version of the output data matrix can be used $\tilde{Y}_{1:T-1} = Y_{1:T-1} - \mathbf{1}_{(T-2)\times N} \otimes y_{\mathrm{eq}}$, where y_{eq} denotes the vector of output variables at the starting equilibrium, $\mathbf{1}_{\ell\times q}$ is an $\ell \times q$ matrix of ones, and $\otimes$ stands for the Kronecker product.

[R1: 11] "5. In Eq. (4), the authors solve the optimization and give the input signal $\hat{u}$. What are $\hat{u}$ and u? Which one will satisfy Eq. (1), or both?"

The symbol u is used to denote the input to the system given in Eq. (1). The problem we consider is to find an optimal value of u, namely $u^\star$, that solves the optimization problem given in Eq. (2). Our data-driven algorithm provides a *candidate* solution $\hat{u}$, which coincides with $u^\star$ when the conditions discussed in Section II-B.1 (that is, when the input data matrix $U_{0:T-1}$ is of full row rank; a condition that is typically satisfied when we collect at least mT randomly generated input experiments). We have clarified this fact in the revised manuscript (lines 122 and 129-131).

[R1: 12] "6. It seems that Eq. (4) is independent with network A, controller B, and outputs C. Why do these important parameters not affect the control signals?"

The reviewer is correct that the data-driven expression in Eq. (4) does not explicitly depend on the model parameters A, B, and C. However, the dependence of Eq. (4) on these parameters is implicit and encoded in the collected data $U_{0:T-1}$, $Y_{1:T-1}$, and Y_T. Thus, even though A, B, and C do not appear in Eq. (4), they do affect the control signals via the data matrices $U_{0:T-1}$, $Y_{1:T-1}$, and Y_T. Notice also that the matrices A, B, and C cannot be used directly to design the control input, since they are assumed to be unknown. We have emphasized this (implicit) dependency in the revised manuscript (lines 127-129).

[R1: 13] "More importantly, in this research, the authors did not discuss the controllability of the networks. In other words, in some conditions, the networks are uncontrollable. Then, how can one design the signals?"

We thank the reviewer for this comment, which allows us to clarify an important point.

First, we stress that in the paper we make the assumption that the desired target y_f is output controllable in T steps (lines 82-83 of the revised manuscript), that is, that a control input exists to drive the output y to the desired value y_f in T steps. A system is said to be output controllable (in T steps) if every target $y_f \in \mathbb{R}^p$ is output controllable (in T steps, respectively). The condition of network controllability implies output controllability. Yet, the converse is not true, which means that our assumption of output controllability is less restrictive than the common assumption of network controllability. Further, since network controllability is a structural property that is generically satisfied for sufficiently dense (connected) networks[4], so is our definition of output controllability. In other words, our assumption of output controllability is mild, generically satisfied, and required for the feasibility of the optimization problem given in Eq. (2). In the Supplement of the revised manuscript we have dedicated a new section (Section I) to a discussion of our output controllability assumption and its relation to classic (full) controllability.

Second, we point out that, using our framework, it is possible to check (output) controllability *directly from data*. Specifically, assuming that the control horizon satisfies $T \geq n$, then the network is output controllable if Y_T is of full (row) rank. The latter sufficient condition becomes also necessary when the (row) rank of the input data matrix $U_{0:T-1}$ is full[5]. These results follow from the fact that the columns of matrix Y_T span the space of outputs that can be reached in $T \geq n$ steps using a linear combination of the columns of $U_{0:T-1}$ as inputs. In the revised version of the paper, we have included the above data-based controllability criterion (Section II-B.1 of the main text and Section IV of the Supplement).

[R1: 14] "We also know that the network topology (heterogeneity and density), selections of driver nodes, and output nodes are all important to networks' controllability. It will be great if the authors show how do these factors determine the results."

[4] More precisely, if self-loops are allowed, then connectivity suffices for the network to be controllable for almost all choices of the weights, independently of the choice of control nodes [22–24].

[5] We remark that $U_{0:T-1}$ is typically of full row rank when the input experiments are chosen randomly.

The reviewer is correct that the properties of the network and the selection of driver nodes affect both the theoretical and practical controllability of a network, and so also the results of our paper.

From a theoretical viewpoint, following our reply to [R1: 13], we note that (output) controllability is generically satisfied for connected networks provided that self-loops are allowed. Thus, loosely speaking, our output controllability assumption is always satisfied in practice, and our data-driven results work for almost all (connected) networks, independently of their structure and the location of input/output nodes.

From a practical viewpoint, assuming that we known exactly the network structure (for the model-based expression) and we have collected enough data (for the data-driven expression), then the data-driven and model-based expressions share similar properties as both correspond to the unique solution to the linear-quadratic optimization problem given in Eq. (2). In this case, as it happens for the minimum-energy control problem ($R = I$, $Q = 0$), the solutions to the problem given in Eq. (2) will have different values depending on the properties of the network and the location of input/output nodes. In the simpler minimum-energy case, it is known that symmetric networks with a small number of control nodes feature a control energy that grows exponentially with the size of the network [1, 3], and their control energy can be substantially reduced by decreasing the number of output nodes [25]. However, to the best of our knowledge, a complete description of the relations between the properties of the network, the location of input/output nodes, and the properties of the associated optimal controls are currently lacking, even in the model-based scenario. On the one hand, we think that our data-driven framework may turn out to be helpful in uncovering some of these relations, and we now mention this fact in the Discussion of the revised manuscript. On the other hand, we believe that the analysis of these relations goes far beyond the scope of our paper (which is that of showing that some network control problems can be solved efficiently by relying on data only).

On a related note, we mention that the density of the network seems to play a key role in the numerical accuracy gap between the data-driven and model-based solutions when the network is reconstructed from data, as discussed in our reply to [R1: 2]. Specifically, the denser the network, the higher the gain in numerical accuracy offered by the data-driven solution. We found this relation to hold for both homogenous and heterogenous networks.

[R1: 15] "7. The authors numerically checked the minimum data. How does the minimum data depend on the parameters we mentioned above, and where (the direction) you want to control?"

This is a good point, thanks. The minimum data $N_{\min}$ required to solve the problem given in Eq. (2) of the main text depends both on the direction y_{f} and the available data matrices (which are in turn implicitly dependent on the model parameters A, B, and C). An extreme case is when the data contain a single input experiment which is optimal to reach the target y_{f}; in this case $N_{\min} = 1$. However, it is possible to establish a general upper bound on $N_{\min}$ independent of y_{f} under some mild conditions on the data. Namely, if $U_{0:T-1}$ has full row rank (i.e., if $U_{0:T-1}$ contains mT linearly independent experiments), then $N_{\min} \leq mT$. We stress that linear independence of the control experiments is normally satisfied when the experiments are generated randomly. Further, if the number of independent trials is smaller than mT but such that y_{f} belongs to the range space of Y_T, then the data-driven control still correctly steers the network output to y_{f} in T steps, even though it is not typically optimal. In the revised manuscript, we have largely rewritten Section II-B.1 of the main text (and part of Section III of the Supplement) to make these points clear.

[R1: 16] "8. In the "numerical and computational benefits of data-driven controls," the authors use ER networks with n nodes and m control nodes, where m is about $n/10$ or $n/100$. We know that the minimal number of driver nodes controlling an ER network with linear dynamics follows $\exp\{-\langle k \rangle/2\}$. In other words, if the network is too sparse, $n/10$ nodes may not be able to control the network. "

Thank you for this comment. The reviewer is correct that an ER network without self-loops and with few control nodes can be uncontrollable if it is too sparse. In our numerical simulations we always guarantee the output controllability of ER networks (a standing assumption in our paper) by ensuring that the networks are connected and by setting a threshold to the numerical rank of the output controllability matrix of the network. Specifically, we selected realizations of the ER model that are connected and sets of input/output nodes that make the smallest singular value of the output controllability matrix no smaller than 10^{-10}. We have clarified this implementation detail in the Methods section of the revised manuscript.

[R1: 17] "FURTHERMORE, THE CONTROLLABILITY ALSO DEPENDS ON HOW YOU SELECT THE DRIVER NODES. I SUGGEST THE AUTHOR WILL DISCUSS THESE ISSUES MORE."

The reviewer is correct that network controllability depends on the selection of the control nodes. However, following our replies to comments [R1: 13] and [R1: 14], controllability is a structural property, that is generically achieved for almost all choices of networks and choices of control nodes. More precisely, assuming that self-loops are allowed, if a network is connected and the weights are chosen randomly, then such a network will be controllable with probability one independently of the choice of control nodes. See for instance Refs. [22–24]. While the properties of the solutions to Eq. (2) depend on the location of control nodes and network weights, the feasibility of Eq. (2), and thus the existence of the control inputs that we compute and validate, is generically independent of the choice of control nodes. We have clarified these points in the revised manuscript (Section I of the Supplement).

Comments by Referee # 2

[R2: 1] "As we know, to solve the empirical problem of network science, identifying the network structure and dynamics details is a premise work. However, errors and limitations are unavoidable in this process, and obviously can influence the subsequent studies. Focus on the network controllability, this manuscript tries to overcome this problem by learning control strategy directly from data. Through simulations, the authors showed that their data-driven method are more accurate and computationally more efficient than model-based ones. This indicates that many real control problems might be solved directly and simply from the experimental data. I think that this is an important step for the research of network controllability, and therefore I recommends publication."

We thank the reviewer for their positive evaluation of our work. We are delighted to hear that the reviewer enjoyed our paper and found it suitable for publication in *Nature Communications*.

[R2: 2] "Besides, I have two questions. First, we know that real networks often have scale-free or small-world effects. Does this data-driven method work well for such networks?"

We thank the reviewer for this comment.

From a theoretical viewpoint, our data-driven method works (almost) independently of the properties of the underlying network. In fact, the only assumption that we make is that the desired target y_{f} is output controllable in T steps (see lines 82-83 in the revised manuscript), which requires the existence of an input that steers the output variables to y_{f} in T steps. Output controllability is implied by the classic controllability notion, which is in turn a generic property (see also our replies to comments [R1: 13] and [R1: 17]). We point out that if a network (with self-loops) is connected and the weights are chosen randomly, then such a network will be controllable with probability one independently of the choice of the control nodes [22–24]. Thus, loosely speaking, we can say that our methods also apply to most network systems. We have clarified this fact in the revised manuscript (Section I of the Supplement).

From a numerical viewpoint, we observed that our data-driven expressions typically work better than model-based ones when the network model is reconstructed from data. In particular, the gain in numerical accuracy is particularly significant for dense networks. This could be a consequence of the fact that the identification of the network introduces a number of numerical errors (which are subsequently propagated in the computation of the model-based control) whose magnitude is likely to be proportional to the number of to-be-identified edges. In the revised version of the paper, we have added the new numerical studies of Fig. R5 and Fig. R6, where we investigate the numerical accuracy of model-based and data-driven solutions for different random network models, including scale-free and small-world topologies (we also refer the reviewer to our reply to [R1: 2] for a related discussion).

[R2: 3] "Second, the authors give some figures to show the effects of the system size on the performance of their method, such as Fig.1 (c), Fig.2.(c), and Fig.3. In statistical physics, such a size-dependent property is often studied as finite-size scaling, which can be used to reveal or predict the property of the infinite system. My question is that can some scaling behaviors be extracted from these figures. We know that the real network often has large size. Can these results be used to predict the performance of a much large system?"

The reviewer raises an important point, which is linked to the control energy scaling in complex networks. First, we mention that in the study of minimum-energy (and, more generally, quadratically-optimal) control problems the "critical" parameter is not solely the network dimension n, but rather the ratio between n and the number of control nodes m. Indeed, the control energy and numerical performance of optimal controls

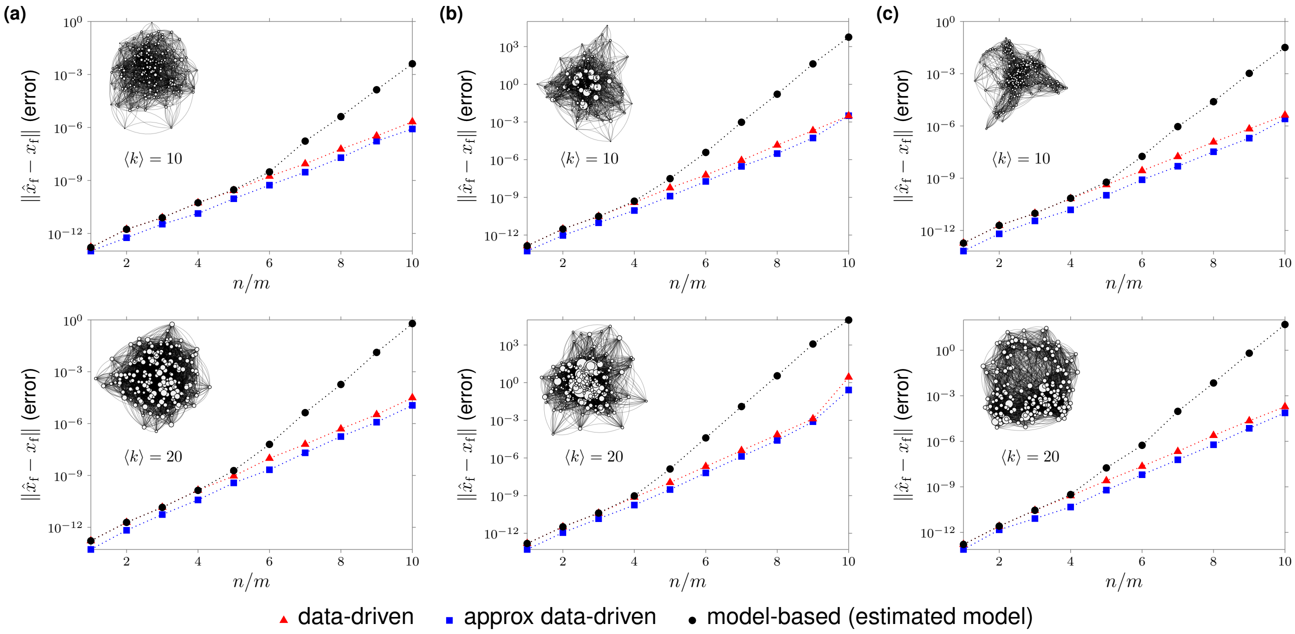

Figure R5: **Numerical accuracy of data-driven and model-based controls.** We compare the error in the final state generated by the data-driven control inputs (Equations (5) and (6) of the main text) and the model-based minimum-energy input for a fixed number of control nodes $m = 100$ and increasing dimension $n \in [100, 1000]$. The model-based control has been computed by first estimating matrices A and B from data according to the subspace-based method described in Methods, and then applying the model-based control expression. In Panel **(a)**, we consider an Erdös–Rényi network with average degree $\langle k \rangle = 10$ (top) and $\langle k \rangle = 20$ (bottom). In Panel **(b)**, we consider a Barabási–Albert network [5] with initial number of nodes $m_0 = 20$ and average degree $\langle k \rangle = 10$ (top) and $\langle k \rangle = 20$ (bottom). In Panel **(c)**, we consider a Watts–Strogatz network [6] with probability of rewiring $p_{\text{rew}} = 0.2$ and average degree $\langle k \rangle = 10$ (top) and $\langle k \rangle = 20$ (bottom). In all plots we use a control horizon $T = 15$ and a number of data $N = mT + 200$ generated using i.i.d. normal inputs. To limit the influence of eigenvalues in the computation of optimal controls across different network models, we normalize the adjacency matrix A by its norm $\|A\|$. The m control nodes and the final state x_f are randomly generated, with the only constraint that the resulting system is controllable (see Methods for further details). The curves represent the average over 100 realizations of networks, data, control nodes, and final states.

is approximately the same for networks generated via the same model and featuring the same ratio n/m. As n/m grows, the energy to control a network typically grows exponentially leading to a poor numerical performance of optimal controls, as shown in a series of papers, e.g., see [1–4] (we refer the reviewer also to our reply to comment to [R1: 2] for a similar discussion). More importantly, as pointed out in, e.g., Ref. [2], the control error may undergo a sharp transition as the ratio n/m increases. We numerically found that this transition occurs for both the model-based and data-driven expressions at nearly the same value of n/m (see Fig. R7). Because of such abrupt behavior, we think that extrapolating the performance of our expressions for large values n/m using a finite-size scaling analysis may not be appropriate nor provide meaningful insights to our analysis. We believe however that a related intriguing problem is to analyze how network properties, such as density and degree-heterogeneity, influence the aforementioned numerical transition for the general cost function in Eq. (2).[6] Our data-driven framework may prove useful to tackle

[6] We mention that this problem has been investigated for the model-based, minimum-energy case in Ref. [2].

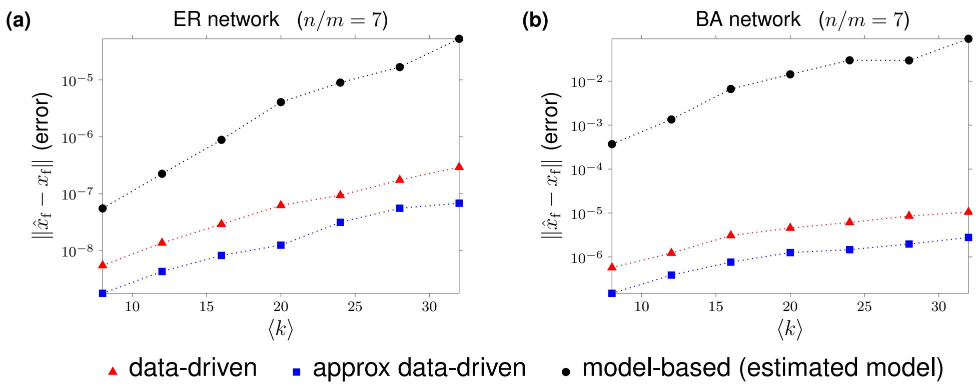

Figure R6: **Effect of average degree on numerical accuracy.** We compare the error in the final state generated by the data-driven minimum-energy control inputs (Equations (5) and (6) of the main text) and model-based expression as a function of the average degree $\langle k \rangle$ for Erdös–Rényi (panel **(a)**) and Barabási–Albert networks (panel **(b)**). We choose $n = 700$ and $m = 100$. The remaining parameters are as in Fig. R5. All curves represent the average over 100 realizations of networks, data, control nodes, and final states.

this and other related controllability problems due to the simplified expressions of optimal controls. We now mention this fact in the Discussion of the revised manuscript. However, we also believe that such an investigation would require substantially different techniques that would probably deviate considerably for the main objective of this paper, which is to show that it is possible to compute optimal control inputs relying on data and without using a detailed model of the network.

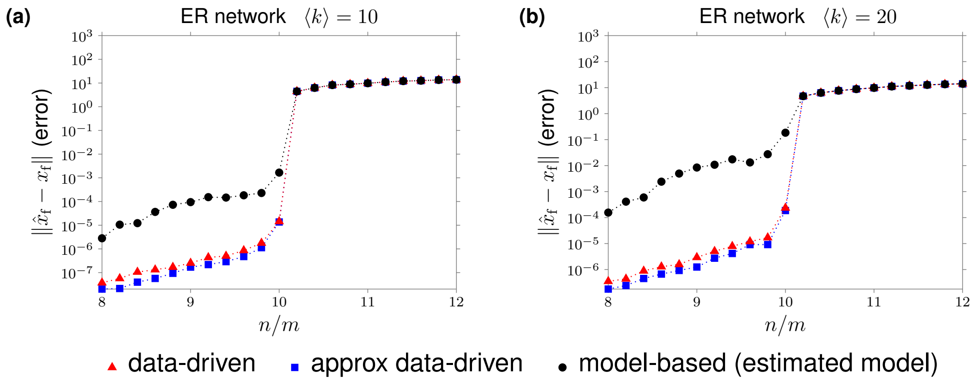

Figure R7: **Controllability transition in ER networks.** We compare the error in the final state generated by the data-driven control inputs (Equations (5) and (6) of the main text) and the model-based minimum-energy control nodes as a function of the ratio n/m for Erdös–Rényi networks with two different values of the average degree, namely $\langle k \rangle = 10$ (panel **(a)**) and $\langle k \rangle = 20$ (panel **(b)**). The remaining parameters are as in Fig. R5.

Comments by Referee # 3

[R3: 1] "This paper solves the discrete time, finite-time, open loop, linear-quadratic optimal control problem using a set of input-output data rather than the system matrices (A, B, C) directly. While there is much work on learning optimal controls for the discrete time LQR problem (which is solved in the infinite-time and feedback control framework) I was unable to find any previous work that addressed the model-free, finite-time and open loop case, so the main result of this paper appears to be novel."

We thank the reviewer for the positive evaluation of our work and for recognizing the novelty of our approach.

[R3: 2] "Determining controls, in particular optimal controls, that are based on data rather than detailed information about the underlying, possibly extremely complicated, network system, is a topic that should be of interest to a general audience. Nonetheless, I have the following concerns that should be addressed."

We thank the reviewer for their appreciation of our work and their insightful comments. In the itemized response below, we carefully address all of the concerns raised by the reviewer.

[R3: 3] "I recommend, for the general audience, to be specific about the necessary and sufficient conditions for a discrete time linear system, (A, B, C) and T, to be output controllable, rather than just reference the book by Kailath, as this assumption is integral in the application of the remaining results."

Thank you for bringing this up. In the revised manuscript we have added a new section to the Supplement (Section I) where we report the formal definition of (T-steps) output controllability of a discrete-time linear time-invariant (LTI) system, and we recall a necessary and sufficient condition for a discrete-time LTI system to be output controllable. We have also replaced the reference to Kailath's book with a more accurate one, namely Ref. [26]. Notice that our assumption of output controllability is implied by the classic notion of controllability (and reduces to it when $C = I$), but the converse is not true. This means that our assumption of output controllability is less restrictive than the common assumption of network controllability. In addition, since network controllability is a structural property that is generically satisfied (see also our replies to comments [R1: 13] and [R1: 17]), so is our definition of output controllability. Thus, we can say that our methods apply (at least theoretically) to most network systems.

[R3: 4] "The requirements on T is important and is an important difference from the continuous time case where controllability does not depend on the final time T."

Thanks. The reviewer is correct that T plays a key role in the controllability analysis of discrete-time systems, and this is in sharp contrast with the continuous-time case. We have highlighted this important difference in the revised version of the Supplement (Section I).

[R3: 5] "Fig. 1C, edge weight uncertainty is zero, but there is still an error present in the final state, why is this? shouldn't it be exact?"

Thank you for raising these questions. Although there is no uncertainty on the edge weights, the presence of an error in the final state is due to numerical errors in the computation of the optimal control sequence. Thus, although theoretically exact, the optimal control input is always affected by numerical uncertainty that is reflected in an error in the final state. We stress that this numerical error is typically significant for sufficiently large values of the network dimension n (assuming that the number of control nodes m is limited) because of the well-known ill-conditioning properties of the classic Gramian-based control formula [1, 2]. We added a sentence to clarify this fact in the revised version of the paper (caption of Fig. 1).

[R3: 6] "What is the difference between u^* and $\hat{u}$ in Eq. (2) and Eq. (4), isn't Eq. (4) suppose to be the solution of Eq. (2)? Reading on I see that $\hat{u}$ is the 'data-driven' input while u^* is the true optimal solution. This should be stated earlier."

The reviewer is correct. The symbol $u^\star_{0:T-1}$ denotes the optimal T-steps control sequence, whereas $\hat{u}_{0:T-1}$ denotes the data-driven control sequence. We use a different notation because, in general, $u^\star_{0:T-1} \neq \hat{u}_{0:T-1}$. A sufficient condition for the equality $u^\star_{0:T-1} = \hat{u}_{0:T-1}$ to hold true is that the input data matrix $U_{0:T-1}$ contains $N \geq mT$ independent columns. We have clarified this fact in Sec. II-B of the revised version of the paper after Eq. (4) (lines 129-131).

[R3: 7] "When deriving the Minimum-Energy Control Input, in section IV in the SI, it is stated that for $Q = O$ and $R = I$ one can replace $L = I$. But from section I in the SI I believe it should be that $L = U^T_{0:T-1} U_{0:T-1}$ where the input data could be constructed such that $L = I$ but for arbitrary inputs I do not believe this is the case."

Thank you for pointing out this mistake. As correctly stated by the Reviewer, the condition $L = I$ is incorrect. The correct condition is $L = ZU_{0:T-1}$, where $Z \in \mathbb{R}^{mT \times mT}$ is an arbitrary orthogonal matrix. In other words, L should satisfy the condition $L^\top L = U^\top_{0:T-1} U_{0:T-1}$. We have fixed this mistake in the revised version of the Supplement.

[R3: 8] "In Eq. (6) in the main text, an approximate data-driven input for the Minimum Energy problem is presented, but it reappears as an exact equality in Eq. (14) in the SI. Could the authors please explain this discrepancy?"

Thanks for pointing out this discrepancy. The equality in Eq. (14) should be intended in the limit of an infinite number of data (i.e., as $N \to \infty$). More precisely, the approximate control input sequence $U_{0:T-1} Y_T^\dagger y_\mathrm{f}$ tends almost surely to the data-driven control $\hat{u}_{0:T-1} = (Y_T U^\dagger_{0:T-1})^\dagger y_\mathrm{f}$ as the number of data grows to infinity, provided that the input $U_{0:T-1}$ has full row rank and the entries of $U_{0:T-1}$ are i.i.d. Gaussian random variables with zero mean and finite variance $\sigma^2 \neq 0$ (cf. Theorem VII.2 of the revised Supplement). Note also that, in this case, the data-driven control $\hat{u}_{0:T-1}$ coincides with optimal one $u^\star_{0:T-1}$. In the revised version of the paper, we have resolved the notational discrepancy by introducing a new symbol to denote the approximate control input $U_{0:T-1} Y_T^\dagger y_\mathrm{f}$, namely we now write $\tilde{u}_{0:T-1} = U_{0:T-1} Y_T^\dagger y_\mathrm{f}$.

[R3: 9] "In the application to the New England power grid, I have a few questions: (1) Is the data collected from the linearized model or the original continuous nonlinear model?"

The data are collected from the original nonlinear model (i.e., the swing dynamics in Eq. (14) of the main text), which has been discretized for numerical simulation. Specifically, we perturb the frequencies of the generators (our control variables) around the equilibrium using a small input and we record the corresponding output. To numerically simulate the continuous-time behavior of the nonlinear system we use a fixed-step forward-Euler discretization of the nonlinear dynamics (using a sufficiently small discretization step, namely $T_s = 2.5 \times 10^{-4}$s), as described in Methods. We have emphasized this fact in the revised version of the paper (lines 317 and 471). This example, together with our other examples, shows that our data-driven solution can also be applied to systems with nonlinear dynamics. While we do not expect this to be the case for all classes of nonlinear systems and operating conditions, it is reassuring to see that our analysis is robust to some dynamical nonlinearities. We have added an additional example in the revised manuscript to further validate the applicability of our methods for nonlinear networks (see also our reply to comment [R1: 5]).

[R3: 10] "(2) Is the simulation with the control input done with the linearized discrete model? Or is the discrete time control input applied the linearized model?"

The simulation is carried out using the real nonlinear model. While our methods are provably correct for linear networks, these results further validate the applicability of our methods to nonlinear systems, at least in certain regimes and operating conditions.

[R3: 11] "I ask these questions because linearizations are only valid close to the point about which the linearization is applied and I do not think this subtlety is addressed in the discussion about this application."

The reviewer is correct. The objective of our simulations is not to claim that linear controls are always effective for nonlinear systems. Rather, we aim to show that our methods, which are provably correct for linear networks, can also be used in certain nonlinear networks, possibly with approximate performance. Since the linearization around the equilibrium point is only valid in a region close to the equilibrium point, in our example, the data-driven control input does not steer the state of the network back to its original value, but to a point close to it (see the insets of Fig. 5(c) of the main text). This result shows that our data-driven input is only approximately correct, yet sufficient for restoring the correct behavior of the grid. We also stress that the matrix Q in the optimization problem given in Eq. (2) can be tuned to favor trajectories with a small deviation from the equilibrium, thus facilitating the applicability of our methods to the considered study (see also our reply to comment [R1: 10]). We have clarified these important considerations in Section II-C.1 of our revised manuscript (lines 322-327).

References

[1] F. Pasqualetti, S. Zampieri, and F. Bullo. Controllability metrics, limitations and algorithms for complex networks. *IEEE Transactions on Control of Network Systems*, 1(1):40–52, 2014.

[2] J. Sun and A. E. Motter. Controllability transition and nonlocality in network control. *Physical Review Letters*, 110(20):208701, 2013.

[3] G. Yan, G. Tsekenis, B. Barzel, J. J. Slotine, Y. Y. Liu, and A. L. Barabasi. Spectrum of controlling and observing complex networks. *Nature Physics*, 11(9):779–786, 2015.

[4] L.-Z. Wang, Y.-Z. Chen, W.-X. Wang, and Y.-C. Lai. Physical controllability of complex networks. *Scientific reports*, 7:40198, 2017.

[5] A. L. Barabási and R. Albert. Emergence of scaling in random networks. *Science*, 286(5439):509–512, 1999.

[6] D. J. Watts and S. H. Strogatz. Collective dynamics of 'small-world' networks. *Nature*, 393(6684):440–442, 1998.

[7] K. J. Åström and R. M. Murray. *Feedback systems: an introduction for scientists and engineers*. Princeton university press, 2010.

[8] S. Zhao and F. Pasqualetti. Networks with diagonal controllability gramians: Analysis, graphical conditions, and design algorithms. *Automatica*, 102:10–18, 2019.

[9] R. Hegselmann and U. Krause. Opinion dynamics and bounded confidence models, analysis, and simulations. *Journal of Artificial Societies and Social Simulation*, 5(3), 2002.

[10] P. M. DeMarzo, D. Vayanos, and J. Zwiebel. Persuasion bias, social influence, and unidimensional opinions. *The Quarterly Journal of Economics*, 118(3):909–968, 2003.

[11] A. Schnitzler and J. Gross. Normal and pathological oscillatory communication in the brain. *Nature Reviews Neuroscience*, 6(4):285, 2005.

[12] F. Dörfler, M. Chertkov, and F. Bullo. Synchronization in complex oscillator networks and smart grids. *Proceedings of the National Academy of Sciences*, 110(6):2005–2010, 2013.

[13] Y. Tang, F. Qian, H. Gao, and J. Kurths. Synchronization in complex networks and its application-a survey of recent advances and challenges. *Annual Reviews in Control*, 38(2):184–198, 2014.

[14] F. Dörfler and F. Bullo. Synchronization in complex networks of phase oscillators: A survey. *Automatica*, 50(6):1539–1564, 2014.

[15] D. A. Wiley, S. H. Strogatz, and M. Girvan. The size of the sync basin. *Chaos: An Interdisciplinary Journal of Nonlinear Science*, 16(1):015103, 2006.

[16] S. Gu, F. Pasqualetti, M. Cieslak, Q. K. Telesford, B. Y. Alfred, A. E. Kahn, J. D. Medaglia, J. M. Vettel, M. B. Miller, S. T. Grafton, and D. S. Bassett. Controllability of structural brain networks. *Nature Communications*, 6, 2015.

[17] A. Vinayagam, T. E. Gibson, H.-J. Lee, B. Yilmazel, C. Roesel, Y. Hu, Y. Kwon, A. Sharma, Y.-Y. Liu, N. Perrimon, and A. L. Barabási. Controllability analysis of the directed human protein interaction network identifies disease genes and drug targets. *Proceedings of the National Academy of Sciences*, 113(18):4976–4981, 2016.

[18] G. Yan, P. E. Vértes, E. K. Towlson, Y. L. Chew, D. S. Walker, W. R. Schafer, and A.-L. Barabási. Network control principles predict neuron function in the caenorhabditis elegans connectome. *Nature*, 550(7677):519, 2017.

[19] J. Z. Kim and D. S. Bassett. Linear dynamics and control of brain networks. In *Neural Engineering*, pages 497–518. Springer, 2020.

[20] W. J. Rugh. Analytical framework for gain scheduling. *IEEE Control Systems Magazine*, 11(1):79–84, 1991.

[21] G. F. Franklin, J. D. Powell, and A. Emami-Naeini. *Feedback control of dynamic systems*. Prentice hall Upper Saddle River, 1997.

[22] K. J. Reinschke. *Multivariable Control: A Graph-Theoretic Approach*. Springer, 1988.

[23] N. J. Cowan, E. J. Chastain, D. A. Vilhena, J. S. Freudenberg, and C. T. Bergstrom. Nodal dynamics, not degree distributions, determine the structural controllability of complex networks. *PLoS ONE*, 7(6):e38398, 2012.

[24] T. Menara, D. S. Bassett, and F. Pasqualetti. Structural controllability of symmetric networks. *IEEE Transactions on Automatic Control*, 64(9):3740–3747, 2019.

[25] I. Klickstein, A. Shirin, and F. Sorrentino. Energy scaling of targeted optimal control of complex networks. *Nature communications*, 8:15145, 2017.

[26] P. E. Sarachik and E. Kreindler. Controllability and observability of linear discrete-time systems. *International Journal of Control*, 1(5):419–432, 1965.

Reviewer #1 (Remarks to the Author):

The authors have addressed all my previous, and the current version is much better. However, I still have some concerns and comments on the newly produced results.

1. In the new version, the authors stated the focuses of this work is the input/output control. Especially in figure 1a, the authors added the outputs. For the input/output control, we have the state variable x and output y. Moreover, the desired final state should be the output y_f. Thus, in figure 1bc the final states should be y_f, but not x_f. Am I right? Furthermore, the authors used y-f in Figure 2, but the mixture of x_f and y_f in Figure 3. In the input/output system, system variable x is assumed to be unknown (or people are not interested in it). That is why you consider y as the measurement. Thus, it is unnecessary to compare the error of x_f.

2. I am trying to understand the advantage of data-driven control. The authors showed that the model-based is unreliable for a small number of driver nodes, and I can observe this from figure 3bcd. Does the model-based model require any data about the system's dynamics? From Eqs. (12-14), it seems that it does not. Then, why in many cases the model-based control is better than data-driven control? For example, the control cost and error in Figure 3 a and e.

3. In Figure 4, the authors use a very small size (10 nodes) and simple topology (circle) network to test the performance on the nonlinear model. I do not understand why the authors did not try it on some random networks (ER or SF) with more nodes.

Reviewer #2 (Remarks to the Author):

The authors give a direct answer to my comment. I agree with their views and response.

Reviewer #3 (Remarks to the Author):

The authors have adequately responded to my previous review for me to recommend publication of their paper.

My one suggestion (that I know may be prohibitively difficult to implement for this paper but that the authors may find useful) is to use high enough precision for numerical examples to demonstrate theoretical results when handling ill-conditioned matrices to ensure values that should theoretically be zero are zero (within the chosen precision). Sometimes numerical errors can lead to erroneous conclusions with regards to the theory. Since the authors use LAPACK, MLAPACK may be of interest.

Comments by Referee # 1

[R1: 1] "The authors have addressed all my previous, and the current version is much better. However, I still have some concerns and comments on the newly produced results."

We thank the reviewer for the positive evaluation of our work and for their insightful new comments, which we address below.

Please note that in the revised manuscript we highlight in blue the passages that have been changed in response to the reviewers' comments and in green the passages that have been changed in response to the editorial requests.

[R1: 2] "1. In the new version, the authors stated the focuses of this work is the input/output control. Especially in figure 1a, the authors added the outputs. For the input/output control, we have the state variable x and output y. Moreover, the desired final state should be the output y_{f}. Thus, in figure 1bc the final states should be y_{f}, but not x_{f}. Am I right? Furthermore, the authors used y_{f} in Figure 2, but the mixture of x_{f} and y_{f} in Figure 3. In the input/output system, system variable x is assumed to be unknown (or people are not interested in it). That is why you consider y as the measurement. Thus, it is unnecessary to compare the error of x_{f}."

The reviewer is correct that in the paper we focus on input/output control. The extensively studied input/state control problem, in which one is interested in steering the state of the system to a desired configuration x_{f}, is a special case of our framework. In Fig. 1 and 2, we consider the case of input/state control ($C = I$) and we used the notation x_{f} to make clear that we focused on this (particular, yet important) case. However, we agree with the reviewer that this may cause some confusion, thus in the revised version we decided to use notation y_{f} in every figure and we added a sentence in the caption of Fig. 1 and 2 to highlight that, in these figures, it holds that $y_{\mathrm{f}} = x_{\mathrm{f}}$.

[R1: 3] "2. I am trying to understand the advantage of data-driven control. The authors showed that the model-based is unreliable for a small number of driver nodes, and I can observe this from figure 3bcd. Does the model-based model require any data about the system's dynamics? From Eqs. (12-14), it seems that it does not. Then, why in many cases the model-based control is better than data-driven control? For example, the control cost and error in Figure 3 a and e."

Thank you for this comment. The reviewer is correct that to compute the model-based control we do not need any data generated by the system, if we have complete and exact knowledge of the system's dynamics (we use the model itself to compute controls in this case, and not data). In Fig. 3(a),(e), the model-based error is smaller than the data-driven one because here we are assuming that we know exactly and beforehand the system's dynamics (i.e., matrices A, B, C). In this case it is indeed expected that the model-based strategy performs better than the data-driven one,[1] since the latter does not exploit any prior information on the system's dynamics but just a set of data. In the more relevant case in which we do not know beforehand the matrices A, B, C but we only have data generated by the system, we can still apply the model-based control strategy by first computing an estimate of A, B, C and then using these estimates in the model-based control formula. In this case, as illustrated in Fig. 3(b),(c),(d), the data-driven strategy is typically more accurate than the model-based one.

A detailed comparison between model-based, model estimation plus control, and direct data-driven control is in fact an active field of research. In this work, we show that direct data-driven control has some numerical advantages with respect to model estimation plus control, for the problem of point-to-point control in linear

[1] It is interesting to note, however, that in these plots the vertical axis is logarithmic and the order of magnitude of the model-based and data-driven errors are approximately the same.

systems. However, we also acknowledge that additional studies are needed to fully settle the question of which control method is more reliable in all different settings (see [1, 2] for some recent literature on this problem). We have slightly modified some sentences of the Introduction and Discussion to clarify this fact.

[R1: 4] "3. In Figure 4, the authors use a very small size (10 nodes) and simple topology (circle) network to test the performance on the nonlinear model. I do not understand why the authors did not try it on some random networks (ER or SF) with more nodes."

Thank you for pointing this out. In the example of Fig. 4, our goal was to show that in a simple architecture of interconnected oscillators our data-driven strategy was able to generate a prescribed phase-locked synchronization pattern. It is certainly interesting to understand if our strategy is still effective for more complex, disordered topologies. However, in such cases a number of issues arise. In particular, while in the ring topology of our example all the possible phase-locked equilibria of the Kuramoto dynamics have been analytically characterized (see, e.g., [3]), for more complex topologies little is know about the existence, location, and stability of such equilibria from an analytical viewpoint (see, e.g., [4]). Thus, it becomes difficult to determine the initial and final states, that is, the initial and target equilibrium configurations, that specify the point-to-point control problem. While we expect our method to work also in those situations (provided that the final state is a stable equilibrium), lack of knowledge of the phase-locked equilibria introduces complications that are beyond the scope of this paper. We added a remark to clarify this issue in the revised paper (lines 293-297).

Comments by Referee # 2

[R2: 1] "The authors give a direct answer to my comment. I agree with their views and response."

We thank the reviewer for their efforts in reviewing our manuscript.

Please note that in the revised manuscript we highlight in blue the passages that have been changed in response to the reviewers' comments and in green the passages that have been changed in response to the editorial requests.

Comments by Referee # 3

[R3: 1] "The authors have adequately responded to my previous review for me to recommend publication of their paper. "

We would like to thank the reviewer for the detailed comments and for helping in improving our manuscript.

Please note that in the revised manuscript we highlight in blue the passages that have been changed in response to the reviewers' comments and in green the passages that have been changed in response to the editorial requests.

[R3: 2] "My one suggestion (that I know may be prohibitively difficult to implement for this paper but that the authors may find useful) is to use high enough precision for numerical examples to demonstrate theoretical results when handling ill-conditioned matrices to ensure values that should theoretically be zero are zero (within the chosen precision). Sometimes numerical errors can lead to erroneous conclusions with regards to the theory. Since the authors use LAPACK, MLAPACK may be of interest."

Thank you very much for this suggestion. We were not aware of the existence of the MLAPACK library, and we will certainly use it in future works. The reviewer is correct that increasing the numerical precision of the computations can reduce the error effects that we see in our plots. However, since we use the same routines to compare model-based and data-driven methods, we believe that the comparisons in the paper remain qualitatively valid (although possibly with different values) even using routines with higher precision. Further, LAPACK routines are commonly used in engineering and scientific computation, making our comparisons also relevant from a practical perspective. We have added a note in the Discussion of the revised paper on this issue, clarifying that higher precision routines can alleviate and possibly alter our numerical values (our theoretical results and the main messages of the paper remain valid independent of the numerical methods).

References

[1] M. Sznaier. Control oriented learning in the era of big data. *IEEE Control Systems Letters*, 2020.

[2] F. Dörfler, J. Coulson, and I. Markovsky. Bridging direct & indirect data-driven control formulations via regularizations and relaxations. *arXiv preprint arXiv:2101.01273*, 2021.

[3] D. A. Wiley, S. H. Strogatz, and M. Girvan. The size of the sync basin. *Chaos: An Interdisciplinary Journal of Nonlinear Science*, 16(1):015103, 2006.

[4] M. Dhagash, N. S. Daleo, F. Dörfler, and J. D. Hauenstein. Algebraic geometrization of the kuramoto model: Equilibria and stability analysis. *Chaos: An Interdisciplinary Journal of Nonlinear Science*, 25(5):053103, 2015.
